# A chiral analog of the bicyclic guanidine TBD: synthesis, structure and Brønsted base catalysis

**DOI:** 10.3762/bjoc.12.176

**Published:** 2016-08-19

**Authors:** Mariano Goldberg, Denis Sartakov, Jan W Bats, Michael Bolte, Michael W Göbel

**Affiliations:** 1 Institute for Organic Chemistry and Chemical Biology, Goethe University Frankfurt, Max-von-Laue-Straße 7, D-60438 Frankfurt am Main, Germany; 2 Institute for Inorganic and Analytical Chemistry, Goethe University Frankfurt, Max-von-Laue-Straße 7, D-60438 Frankfurt am Main, Germany

**Keywords:** absolute configuration, anthrone, cycloaddition, kinetic resolution, lipase

## Abstract

Starting from (*S*)-β-phenylalanine, easily accessible by lipase-catalyzed kinetic resolution, a chiral triamine was assembled by a reductive amination and finally cyclized to form the title compound **10**. In the crystals of the guanidinium benzoate salt the six membered rings of **10** adopt conformations close to an envelope with the phenyl substituents in pseudo-axial positions. The unprotonated guanidine **10** catalyzes Diels–Alder reactions of anthrones and maleimides (25–30% ee). It also promotes as a strong Brønsted base the retro-aldol reaction of some cycloadducts with kinetic resolution of the enantiomers. In three cases, the retro-aldol products (48–83% ee) could be recrystallized to high enantiopurity (≥95% ee). The absolute configuration of several compounds is supported by anomalous X-ray diffraction and by chemical correlation.

## Introduction

**Figure 1:**
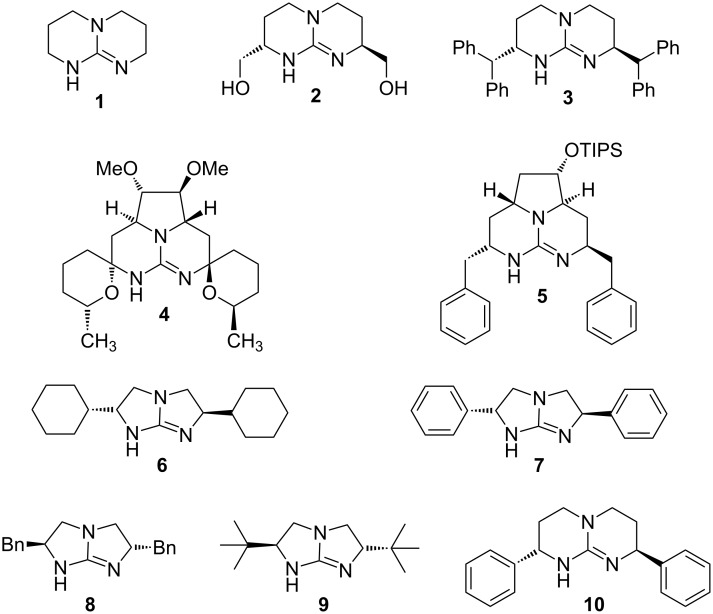
Structure of guanidines **1**–**10**.

In guanidinium ions charge delocalization is an important factor to stabilize the protonated form. As a result, guanidines are exceptionally strong nitrogen bases. As part of the amino acid arginine, they play an important role in biochemistry, mainly by forming ion pairs. In addition, numerous guanidine derivatives with complex cyclic structures can be found in natural products [1]. Simple guanidines such as tetramethylguanidine have been used as strong Brønsted bases in countless applications [2–3]. The bicylic guanidine 1,5,7-triazabicyclo[4.4.0]dec-5-ene (TBD, **1**, Figure 1) [4], another important Brønsted base in preparative chemistry, may also act as a powerful nucleophilic catalyst [3]. Substituted analogs of TBD [5], such as the chiral compound **2**, have become popular in the field of molecular recognition, however, without being tested as catalysts [5–10]. A first example of enantioselective Michael addition has been reported for guanidine **3**, albeit with low selectivity [11]. Compounds **4** and **5**, inspired by the structure of ptilomycalin A and related natural products, have been used as chiral phase-transfer catalysts [12–13].

Bicyclic guanidines with five-membered rings are also known from the alkaloid isoalchornein [14–15]. In subsequent years, synthetic compounds (**6**–**9**) of this structural type have been developed as chiral Brønsted bases [16–19] and used for highly enantioselective Strecker [17] and Diels–Alder reactions [19].

Compared to guanidine **7** ring expansion into structure **10** would shift the stereogenic phenyl groups into closer proximity to a hydrogen-bonded guest molecule and thereby might improve the enantioselective control exerted by **10** when used as a catalyst. In this article we describe the synthesis of guanidine **10** together with some initial applications as a chiral Brønsted base.

## Results and Discussion

**Scheme 1:**
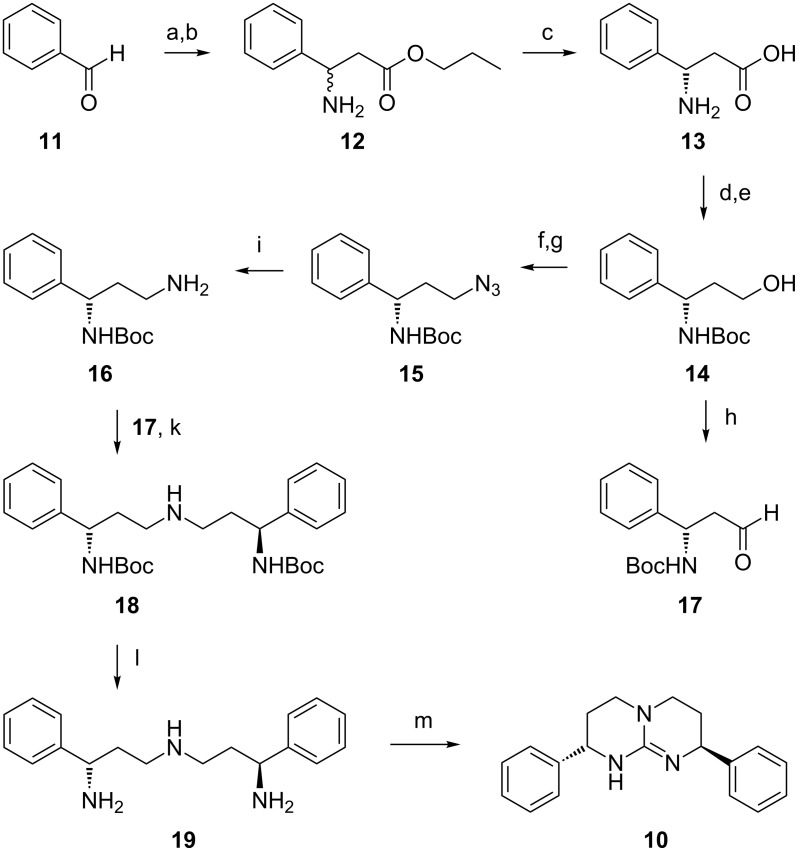
Synthesis of guanidine **10**. Conditions: (a) 1 equiv HOOC-CH_2_-COOH, 2 equiv NH_4_OAc, EtOH, 78 °C, 5 h, 38%; (b) 10 equiv *n-*PrOH, 1.5 equiv H_2_SO_4_, 97 °C, 4 h, 82%; (c) Amano-Lipase PS (from *Burkholderia cepacia*), aqueous Na_2_HPO_4_ buffer, pH 7.00, 50 °C, 1 h, methyl *tert-*butyl ether, 50 °C, 24 h, 90% (45% based on *rac-***12**), >99% ee; (d) 2.5 equiv NaBH_4_, 1.2 equiv I_2_, THF, 66 °C, 18 h, 86%; (e) 1 equiv Boc_2_O, 1.2 equiv triethylamine, CH_2_Cl_2_, 0 °C, 3 h, 100%; (f) 1.1 equiv MsCl, 1.1 equiv triethylamine, CH_2_Cl_2_, 0 °C, 3 h, 79%; (g) 3 equiv NaN_3_, DMF, 24 °C, 120 h, 96%; (h) 2 equiv SO_3_*Py, 2.3 equiv pyridine, 4.1 equiv triethylamine, DMSO, CH_2_Cl_2_, 0 °C, 10 min, 24 °C, 2 h, 87%; (i) H_2_, Pd/C, MeOH, overnight, 84%; (k) 1 equiv **17**, THF, 48 h, 2 equiv NaBH_4_; MeOH, 96 h, 58%; (l) 10 equiv TFA, CH_2_Cl_2_, 40 °C, 24 h, 100%; (m) 1.3 equiv dimethyl trithiocarbonate, MeNO_2_, 101 °C, 2 h, 4 equiv AcOH, 2 equiv MeI, 101 °C, 3 h, 67% as iodide salt **10a**.

Previous syntheses of bicylic guanidines **2** and **6**–**9** started from enantiomerically pure α-amino acids [6,8,10,16–17] or from their reduction products, chiral α-amino alcohols [9,18]. In contrast, our approach used the racemic ester *rac-***12** of β-phenylalanine, easily accessible in a Knoevenagel-type condensation of benzaldehyde **11**, ammonium acetate and malonic acid, followed by esterification (Scheme 1) [20–21]. A kinetic resolution of the enantiomers was achieved by enzymatic hydrolysis with Amano lipase PS from *Burkholderia cepacia* [22–23], a method already optimized for technical use [24]. The best results were obtained with methyl *tert-*butyl ether as a cosolvent [24]. By simple precipitation, batches larger than 15 g of the *S-*configurated acid **13** could be isolated in 90% yield (45% based on *rac*-**12**). In a two-step procedure **13** was converted into amino alcohol **14** without recrystallization in order to keep the enantiomeric excess unchanged. It was determined at this stage to be better than 99%. The *S*-configuration was assigned to **13** in accord with published data [22–24]. Intermediate **14** was converted into amine **16** by mesylation (79%), reaction with NaN_3_ (96%) and hydrogenation (84%). Aldehyde **17**, also accessible from **14** by oxidation (87%), then could be coupled with amine **16** in a reductive amination to form **18** (58%). After removal of the Boc protecting group (quant.), triamine **19** was reacted with dimethyl trithiocarbonate in refluxing nitromethane. The thiourea intermediate was activated in situ by *S*-alkylation with MeI. Upon further heating the final cyclization occured forming the iodide salt **10a** of *S,S*-configurated guanidine **10** (67%). The free base could be isolated by extraction with CH_2_Cl_2_ from 20 M aqueous NaOH (95%). For experimental details see Supporting Information File 1.

**Figure 2:**
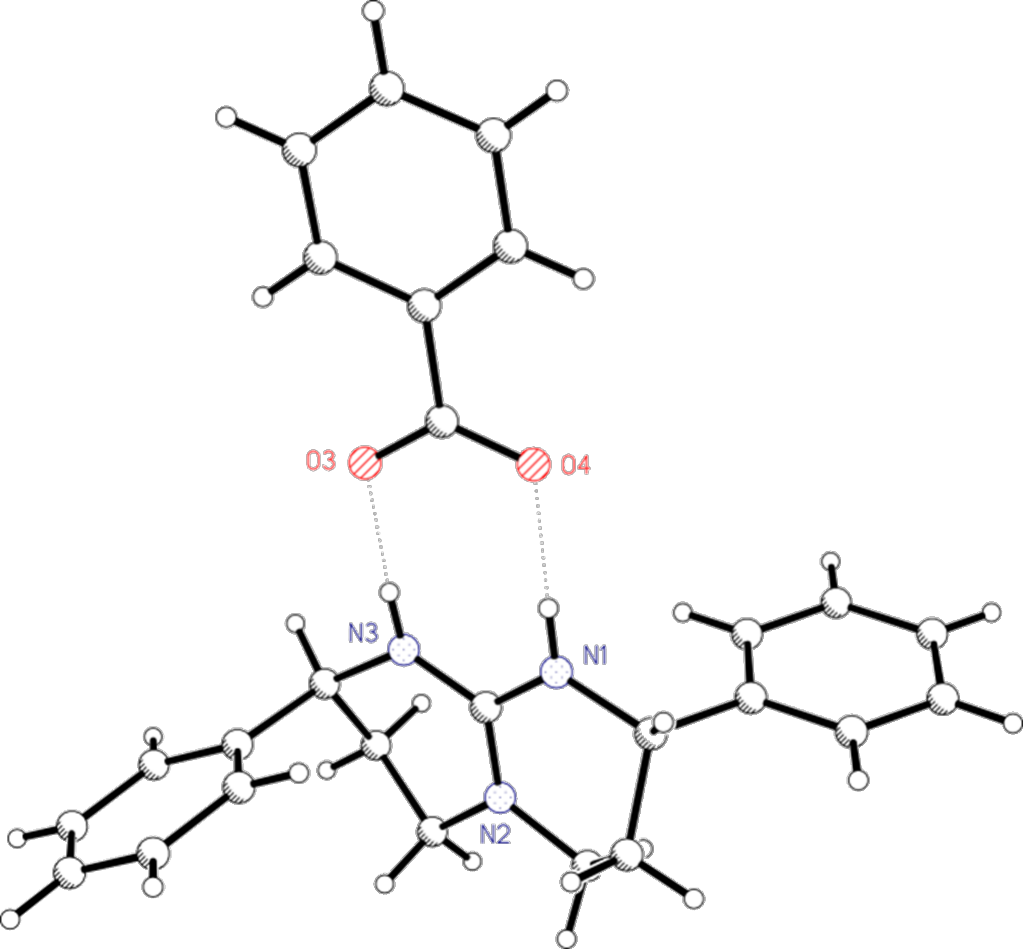
Crystal structure of guanidine **10** as a benzoate salt. Only one of the ion pairs is shown for the sake of clarity.

A crystal structure could be obtained from the benzoate salt of **10** (Figure 2). The asymmetric unit contains two cations, two benzoate anions and ethyl acetate as a solvate molecule. Each cation is connected by two N–H···O hydrogen bonds to benzoate ions. The rings of cation **10** adopt a conformation close to an envelope with the phenyl substituents in pseudo-axial positions. The ion pairs and the solvate molecule are also connected by a number of very weak intermolecular C–H···π (phenyl) and C–H···O contacts (see Supporting Information File 2).

**Scheme 2:**
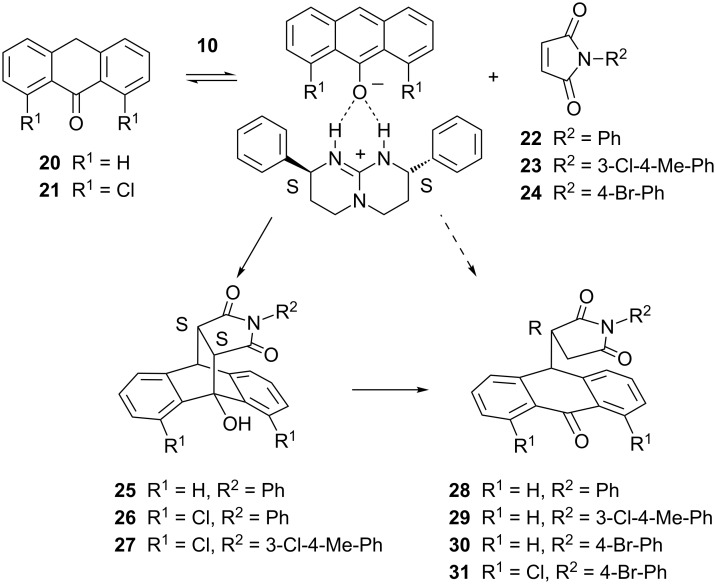
Reaction of anthrones and *N*-arylmaleimides catalyzed by guanidine **10**. The guanidine deprotonates anthrones **20** or **21** and forms chiral ion pairs. Primary products of the ion pair complexes are the Diels–Alder adducts that may be further transformed in a base catalyzed retro-aldol reaction.

The chiral guanidine **10** was tested as a Brønsted base catalyst to promote the reaction of anthrones **20** or **21** with maleimides **22**–**24** (Scheme 2) [19,25–28]. Depending on the substituents and the strength of the Brønsted base either Diels–Alder adducts or Michael products may be formed. Weaker bases such as ion-free bisoxazolines [28] or triethylamine are known to induce Diels–Alder adducts selectively whereas strongly basic guanidines such as **8** may also form the Michael products [19] which are the dominant products in polar solvents [25]. Previous mechanistic studies by Koerner and Rickborn [25] have collected strong arguments for a fast concerted [4 + 2]-cycloaddition of the deprotonated anthrone. Michael products were shown to be secondary products of a base-catalyzed retro-aldol reaction and they are not converted backwards into Diels–Alder adducts under such conditions [19,25–26]. Our results shown below give further support to this view. Riant, Kagan and Ricard have demonstrated for the first time that deprotonated anthrones may coordinate to chiral counterions in less polar solvents. Up to 61% ee could be obtained using cinchona alkaloids as catalysts [26]. The subsequent work of Tan and co-workers with guanidine catalyst **8** achieved enantioselectivities as high as 99% ee [19]. In recent years functionalized chiral amines have been successfully used as catalysts for anthrone maleimide cycloadditions [29–35]. In the presence of **10**, dichloroanthrone **21** reacted with maleimides **22** and **23** to produce exclusively cycloadducts **26** and **27**. In contrast, a mixture of **25** and **28** resulted from the reaction of anthrone **20** and *N*-phenylmaleimide (**22**). For the remaining combinations (**20** + **23**; **20** + **24**; **21** + **24**) only the Michael products **29**–**31** could be observed (0.1 equiv of **10**, CH_2_Cl_2_, −15 °C).

To determine the enantioselectivity of guanidine **10** we started with the structurally simplest case, the reaction of anthrone **20** and *N*-phenylmaleimide (**22**) which finally turned out to be the most complex one (Table 1). The best but still low ee values for cycloadduct **25** were obtained in CHCl_3_ (37% ee) and CH_2_Cl_2_ (34% ee). Much better enantioselectivities of the Michael product **28** were found for reactions in solvents of increased polarity. Assuming that **28** is formed from **25** in a retro-aldol step, one might expect similar ee values for both compounds. This is clearly not the case. In THF the 83% ee for compound **28** strongly deviates from the low value for **25** (6% ee). Obviously, in the reaction of **20** and **22**, a Diels–Alder step with low to moderate stereoselectivity is superimposed with a kinetic resolution of enantiomers in the subsequent retro-aldol reaction. Both are catalyzed by the chiral guanidine **10**. As a result, the numbers shown in Table 1 are not constant but depend on the relative turnover of each reaction.

**Table 1:** Reaction of anthrone **20** and *N*-phenylmaleimide (**22**) in different solvents.^a^

solvent	Diels–Alder product **25**			Michael product **28**	
		
	yield^b^	ee^c^		yield^b^	ee^c^

toluene	36%	22%		51%	47%
(CH_2_Cl)_2_	88%	6%		8%	35%
CHCl_3_	3%	37%		91%	25%
CH_2_Cl_2_	28%	34%		70%	41%
THF	51%	6%		42%	83%
CH_3_CN	15%	1%		53%	74%

^a^All reactions were carried out with 0.1 equiv of guanidine **10** for 64 h at −15 °C. ^b^Isolated yields after chromatographic separation. ^c^Determined by HPLC on a chiral column.

**Figure 3:**
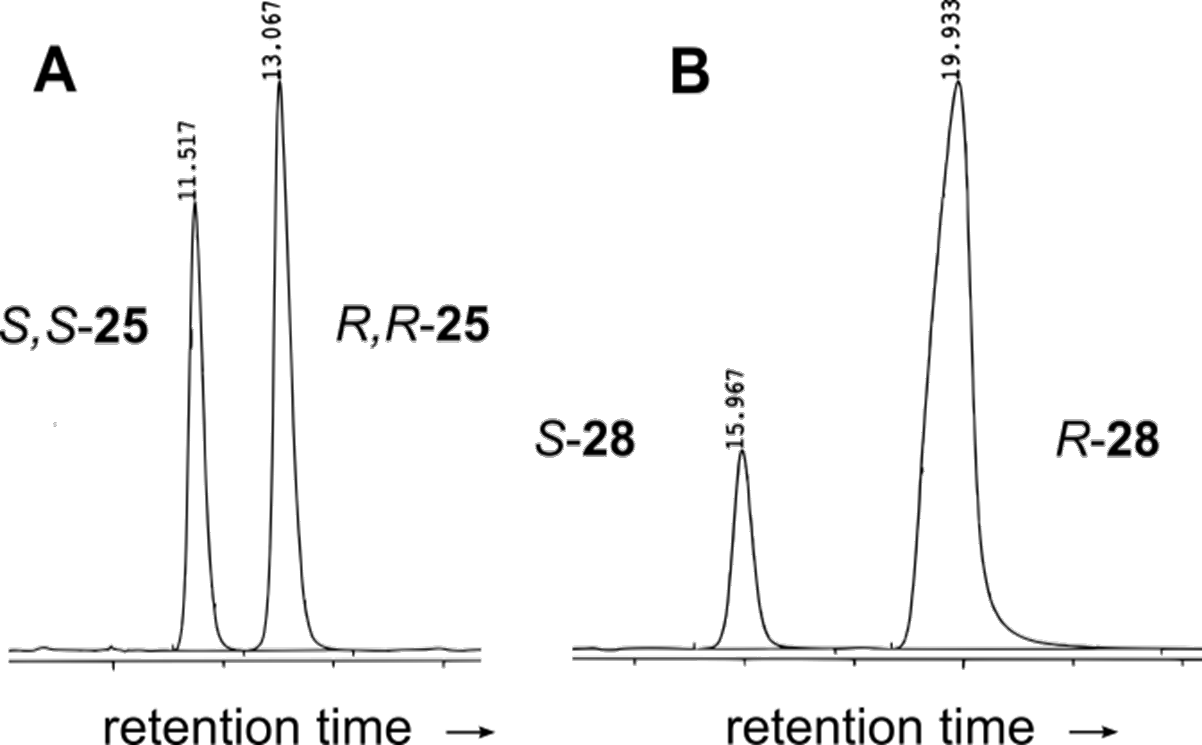
A) Chromatogram of *rac-***25** after incubation with 0.1 equiv of **10** in THF at −15 °C for 64 h. The faster running isomer shows increased conversion into **28**. B) The slower running isomer (*R*) of **28** is formed preferentially. The faster running isomer of **25**, therefore, must be *S,S* configurated.

When *rac-***25** reacted with 0.1 equiv of **10** in THF at −15 °C, at 22% conversion ee values of 18% and 70% were found for compounds **25** and **28**. The dominant isomer of Michael product **28** is shown below to be *R* configurated which corresponds to the slower running peak in Figure 3B. The *R* isomer of **28** is formed from the *S,S* isomer of **25** with retention of configuration (Scheme 2). The change from *S* to *R* is caused by a change in the CIP priorities of the substituents. Thus the faster running smaller peak in Figure 3A must correspond to the *S,S* enantiomer of **25**. Assuming independent first order rate laws for the opening of the *S,S* and *R,R* enantiomers of **25**, the best numerical fit is obtained for *k*_S,S_/*k*_R,R_ ≈ 6.5. When both steps, Diels–Alder and Michael reaction, are catalyzed by guanidine **10** in THF (see Table 1), at 45% conversion the *R* isomer of **28** and the *R,R* isomer of **25** are the dominant species. However, due to the faster reaction of *S,S-***25** the numerical simulation shows that not the *R,R* but the *S,S* isomer is the preferred product of the Diels–Alder step with approximately 33% ee. This value comes close to the numbers obtained for the production of **26** (25% ee; 30% ee in THF) and **27** (28% ee) in CH_2_Cl_2_ when no secondary conversion occurs. In contrast, when compound **21** reacts with **24** ring opening is so fast that no Diels–Alder product accumulates. Here the ee value of the Michael product **31** (21%) must correspond to the stereoselectivity of the Diels–Alder step. Due to low stereoselectivities and fast retro-aldol reactions guanidine **10** is not optimal for the preparation of Diels–Alder adducts.

We therefore focussed our efforts on the Michael products: Anthrone **20** and maleimide **22** reacted with 0.1 equiv of **10** in THF at −15 °C for 64 h. Chromatographic separation then yielded 51% of **25** and 42% of Michael product **28** (83% ee). Recrystallization afforded 32% of **28** with 99% ee. Thus, 12.5 mg of catalyst **10** produced 51 mg of almost pure *R-***28**. Analogous treatment of **20** and **23** yielded 21% of **29** (61% ee). After recrystallization 15% of **29** with 98% ee were obtained. Finally, Michael product **30**, obtained from **20** and **24** (42%, 48% ee) yielded 26% of material with 95% ee.

**Scheme 3:**
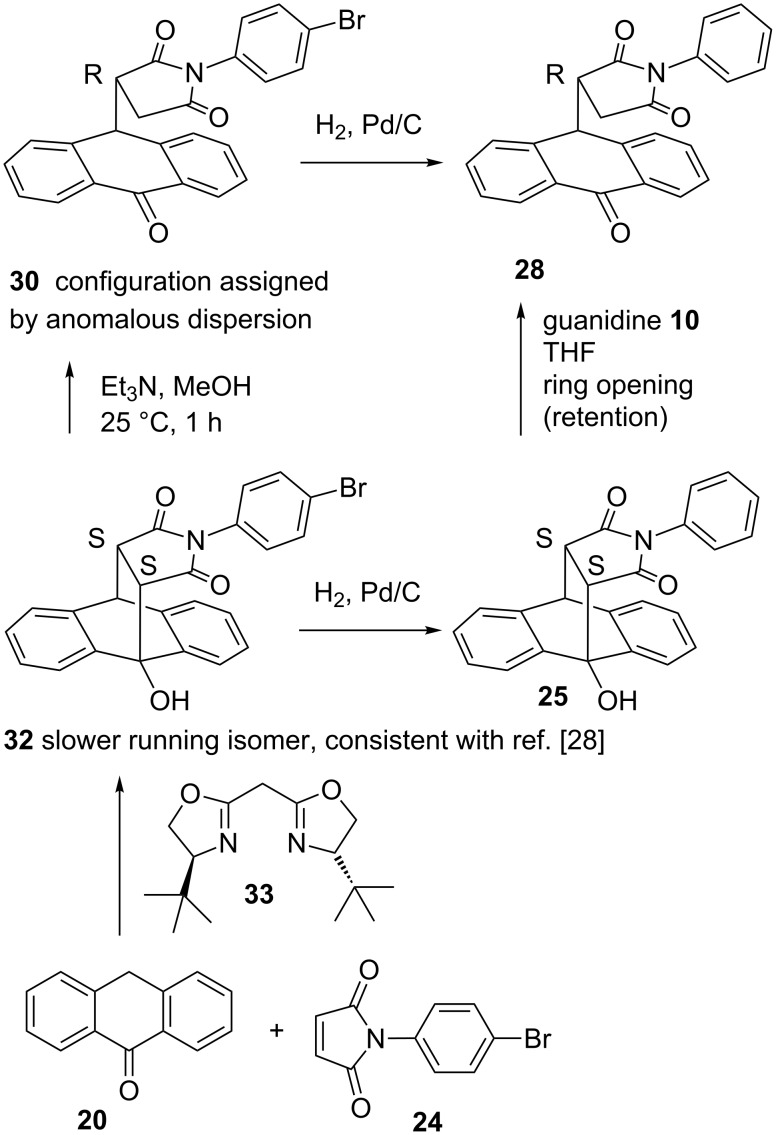
Assignment of the absolute configurations by chemical correlation. The *R* configuration of compound **30** is derived from the crystal structure.

**Assignment of absolute configurations** (Scheme 3): Well grown crystals of enantiopure Michael product **30** suitable for X-ray structural analysis could be obtained by a second recrystallization from CH_2_Cl_2_/cyclohexane. The 4-bromophenyl residue allowed us to assign the *R* configuration by anomalous dispersion (Supporting Information File 3). This isomer corresponds to the slower running isomer on a Chiralpak IA column. By catalytic hydrogenation with Pd on charcoal the bromo residue of enantiopure **30** was replaced with hydrogen thus converting *R* configurated **30** into *R* configurated Michael product **28** (slower running isomer on Chiralpak IA and preferred product under catalysis with **10**). Both compounds must result from the *S,S* configurated Diels–Alder adducts. In the case of **25** this is the faster running isomer on Chiralpak IA. In an earlier study [28] with bisoxazoline catalyst **33**, we have assigned the *S*,*S* configuration to the major isomer of product **32**. By hydrogenolysis, we could now convert such a sample (40% ee) into **25**. The product *S*,*S-***25** was formed as the dominant product (35% ee) thus confirming our previous assignment. Under mild basic conditions, *S*,*S-***32** finally could be opened into *R* configurated Michael product **30** [25–26] (31% ee) to generate a stereochemically consistent view.

## Conclusion

(*S*)-β-Phenylalanine (**13**), readily accessible by enantioselective hydrolysis of ester *rac-***12**, is the starting material for an efficient synthesis of the chiral TBD analog **10**. The cyclization steps in particular, which convert the triamine **19** into the final guanidine **10**, give considerably higher yields compared to guanidines with five-membered rings such as compounds **6**–**9**. Apart from the better synthetic accessibility the formal ring expansion to the six-membered structure of **10** does not increase the stereoselectivity when it is used as a catalyst. We could not observe the near-perfect selectivities of the Tan catalyst **8** in the base-induced reaction of anthrones and maleimides. Nevertheless ee values of up to 83% for the formal Michael products allowed us to isolate 3 compounds in almost enantiopure form after a single recrystallization. The Tan guanidine **8** [19], the bisoxazoline **33** [28], and a stereochemically related bisamidine [27] consistently favor the *S*,*S* isomer of Diels–Alder adduct **25**. Although guanidine **10** has the opposite configuration, it also forms *S*,*S*-**25** preferentially. Accordingly, transition-state structures in the reactions of guanidines **8** and **10** must differ from each other.

## Supporting Information

File 1 Synthetic procedures, characterization data, copies of chromatograms on chiral columns and of ^1^H and ^13^C NMR spectra.

File 2 X-ray data of guanidine **10** as benzoate salt (CCDC-1482611).

File 3 X-ray data of compound **29** (CCDC-1482612).

File 4 X-ray data of compound **30** (CCDC-1482613).
