# Supplementary material for: A chiral analog of the bicyclic guanidine TBD: synthesis, structure and Brønsted base catalysis
Source: Beilstein J Org Chem. 2016 Aug 19;12:1870–6. doi: 10.3762/bjoc.12.176 (PMC5082680; doi:10.3762/bjoc.12.176)

Supporting Information

for

A chiral analog of the bicyclic guanidine TBD: synthesis, structure and Brønsted base catalysis

Mariano Goldberg[1], Denis Sartakov[1], Jan W. Bats[1], Michael Bolte[2] and Michael W. Göbel*[1]

Address: [1]Institute for Organic Chemistry and Chemical Biology, Goethe University Frankfurt, Max-von-Laue-Straße 7, D-60438 Frankfurt am Main, Germany and [2]Institute for Inorganic and Analytical Chemistry, Goethe University Frankfurt, Max-von-Laue-Straße 7, D-60438 Frankfurt am Main, Germany

Email: Michael W. Göbel - m.goebel@chemie.uni-frankfurt.de

* Corresponding author

Synthetic procedures, characterization data, copies of chromatograms on chiral columns and of ^{1}H and ^{13}C NMR spectra

Table of contents:

General information..S2
Synthesis of guanidine **10**..S2
Base-catalyzed reactions of anthrones and maleimides..S9
Determination of absolute configurations by chemical correlation..S12
Chromatograms on chiral columns..S13
X-ray data of compounds **10**, **29**, and **30**..S28
Copies of ^{1}H and ^{13}C NMR spectra of new compounds..S33

General information:

Anhydrous dichloromethane and tetrahydrofuran were purchased from Acros Organics and stored over molecular sieves. Anthrone and maleimides were purchased from Alfa Aesar and used without further purification. Flash column chromatography: silica gel (60 Å pore size, 0.04–0.063 mm particle size, Macherey-Nagel). Analytical thin-layer chromatography: aluminum plates pre-coated with silica gel (60 Å pore size, 0.2 mm, Macherey-Nagel) impregnated with a fluorescent indicator (254 nm). TLC plates were visualized by exposure to ultraviolet light (UV). Proton nuclear magnetic resonance (^{1}H NMR) and carbon nuclear magnetic resonance spectra (^{13}C NMR) were recorded at 300 K with a Bruker AM 250 (^{1}H: 250 MHz; ^{13}C: 63 MHz) or a Bruker AV 500 (^{1}H: 500 MHz; ^{13}C: 126 MHz) NMR spectrometers. Chemical shifts for protons are reported in parts per million (δ scale) and internally referenced to the proton resonances of the solvent ($CDCl_3$: δ 7.26, DMSO-d_6: δ 2.50, D_2O: δ 4.75). Chemical shifts for carbon are reported in parts per million (*δ* scale) and referenced to the carbon resonances of the solvent ($CDCl_3$: δ 77.00, DMSO-d_6: δ 39.51). Data are represented as follows: chemical shift, multiplicity (s = singlet, bs = broad singlet, d = doublet, t = triplet, m = multiplet, dd = double doublet, dt = double triplet, td = triple doublet, tt = triple triplet), coupling constants in Hz, and integration. ESI-MS spectra were obtained on a Fisons VG Plattform II. HRMS spectra were recorded on a MALDI LTQ Orbitrap mass spectrometer from Thermo Scientific. Enantiomeric excess values were determined by chiral HPLC on a Millipore Waters Model 590 with a Model 440 Absorbance Detector. Chiralpak IA was used as a column and Knauer's software Eurochrom 2000 Integration Package for the evaluation. Conditions, if not stated otherwise: *n*-Hexane/isopropanol 10:3 + 20% CH_2Cl_2; 0.7 mL/min, detection at 254 nm.

Optical rotations were recorded on a Perkin Elmer Polarimeter 241 with the thermostat Haake G and Haake D8. Melting points were determined on a Schorpp apparatus MPM-H2 and are uncorrected. Elemental analyses were recorded on an Elementar vario MICRO cube.

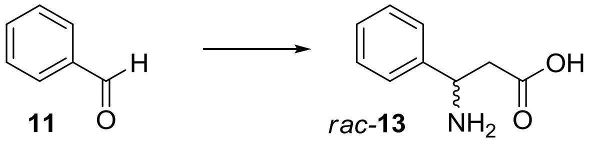

(*R*,*S*)-3-Amino-3-phenylpropanoic acid (*rac*-13): To a solution of malonic acid (80.0 g, 0.77 mol, 1.00 equiv) and benzaldehyde (78.0 mL, 0.77 mol, 1.00 equiv) in 95% ethanol (450 mL), ammonium acetate (118.70 g, 1.54 mol, 2.00 equiv) was added. The reaction mixture was refluxed for 5 h and then allowed to cool to room temperature. After cooling to −20 °C the resulting precipitate was filtered off, washed with cold ethanol and dried in vacuo to give *rac*-**13** as a colourless solid (47.6 g, 0.29 mol, 38%). R_f = 0.33 (MeOH/CH_2Cl_2 2:1). ^{1}H-NMR (500 MHz, D_2O): *δ* = 7.46-7.39 (m, 5 H), 4.60 (dd, *J* = 8.0, 6.5 Hz, 1 H), 2.82 (dd, *J* = 16.2,

8.1 Hz, 1 H), 2.73 (dd, J = 16.2, 6.6 Hz, 1 H) ppm. ^{13}C-NMR (126 MHz, D_2O): δ = 177.2, 135.9, 129.23, 129.20, 126.9, 52.7, 40.4 ppm. MS (ESI): m/z (%) = 166.8 (100) [M + H^+]. HRMS (MALDI): calcd. for $C_9H_{12}NO_2$ [M + H^+]: 166.08626; found 166.08638.

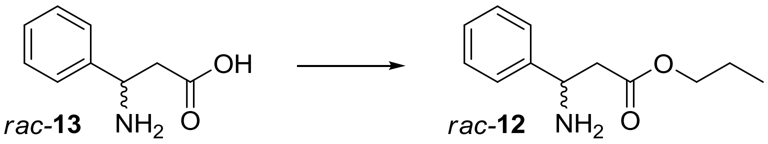

(*R*,*S*)-Propyl 3-amino-3-phenylpropanoate (*rac*-12): To a suspension of 3-amino-3-phenylpropanoic acid (*rac*-**13**) (46.2 g, 0.28 mol, 1.00 equiv.) in *n*-propyl alcohol (210 mL, 2.80 mol, 10.0 equiv) conc. sulfuric acid (22.4 mL, 0.42 mol, 1.50 equiv) was added. The clear solution was refluxed for 4 h and then cooled to room temperature. The reaction mixture was concentrated under reduced pressure and then 6 M aqueous sodium hydroxide solution was added until pH 8.5 was reached. Then, 100 mL of ethyl acetate and 100 mL of water were added. The phases were separated and the aqueous layer was extracted with ethyl acetate. The combined organic layers were dried over $MgSO_4$ and evaporated under reduced pressure to give (*rac*-**12**) as a colourless oil (47.89 g, 0.23 mol, 82%). R_f = 0.30 (*c*-hexane/EtOAc 1:1). ^{1}H-NMR (500 MHz, $CDCl_3$): δ = 7.29-7.22 (m, 4 H), 7.16 (tt, J = 7.1, 1.7 Hz, 1 H), 4.32 (t, J = 6.9 Hz, 1 H), 3.95 (t, J = 6.8 Hz, 2 H), 2.59 (d, J = 6.6 Hz, 2 H), 1.99 (bs, 2 H), 1.54 (sextet, J = 7.3 Hz, 2 H), 0.83 (t, J = 7.5 Hz, 3 H) ppm. ^{13}C-NMR (126 MHz, $CDCl_3$): δ = 171.7, 144.2, 128.2, 127.0, 125.9, 65.7, 52.3, 43.7, 21.6, 10.0 ppm. MS (ESI): m/z (%) = 208.8 (100) [M + H^+]. HRMS (MALDI): calcd. for $C_{12}H_{18}NO_2$ [M + H^+]: 208.13321; found 208.13309.

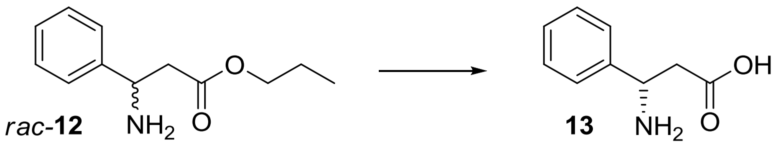

(*S*)-3-Amino-3-phenylpropanoic acid (13): To 115 mL of a 50 mM aqueous solution of disodium hydrogen phosphate dodecahydrate with a pH of 7.00 was added 2.30 g of lipase (Amano Lipase PS; available from Aldrich) and stirred for 1 h at 50 °C. The clear solution was poured into a solution of ester *rac*-**12** (46.25 g, 0.22 mol) in methyl *tert*-butyl ether (115 mL) and stirred for 24 h at 50 °C. After cooling to room temperature 190 mL acetone was added and the reaction mixture was stirred for 1 h at 0 °C. The resulting precipitate was filtered off and dried in vacuo to give **13** as a colourless solid (16.30 g, 0.10 mol, 45%, 90% based on *S*-**12**). $[\alpha]_D^{20}$ = -8.00 ° (c: 0.03, H_2O), Lit.: $[\alpha]_D^{25}$ = -8 ° (H_2O) [1]. R_f = 0.33 ($MeOH/CH_2Cl_2$ 2:1). ^{1}H-NMR and ^{13}C-NMR were identical to the spectra of *rac*-**12**. MS

(ESI): *m/z* (%) = 166.0 (100) [M + H^+]. HRMS (MALDI): calcd. for $C_9H_{12}NO_2$ [M + H^+]: 166.08626; found 166.08619.

[1] G. Tasnádi, E. Forró, F. Fülöp, *Tetrahedron: Asymmetry* **2008**, *19*, 2072-2077.

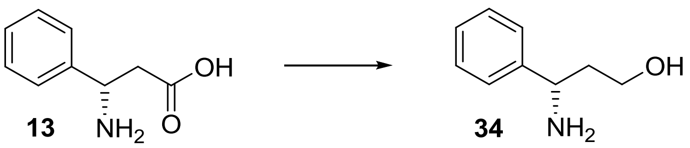

(*S*)-3-Amino-3-phenylpropan-1-ol (34): To a suspension of (*S*)-3-amino-3-phenylpropanoic acid (**13**, 16.22 g, 0.098 mol, 1.00 equiv) and $NaBH_4$ (9.46 g, 0.25 mol, 2.55 equiv) in dry THF (350 mL) at 0 °C and under an argon atmosphere was added very slowly a solution of I_2 (30.46 g, 0.12 mol, 1.20 equiv) in dry THF (95 mL). The reaction mixture was refluxed for 18 h and then cooled to room temperature. Methanol was added drop by drop until the solution became clear. After stirring for 1 h at room temperature the solvent was removed under reduced pressure. To the residue was added 20% aqueous KOH (300 mL) and the mixture stirred for 4 h at room temperature. The aqueous phase was extracted with CH_2Cl_2 and washed with water. The combined organic layers were dried over $MgSO_4$ and evaporated under reduced pressure to give **34** as a colorless oil (12.78 g, 0.085 mol, 86%). R_f = 0.42 ($MeOH/CH_2Cl_2$ 2:1). ^{1}H-NMR (500 MHz, $CDCl_3$): δ = 7.32 (t, *J* = 7.3 Hz, 2 H), 7.27-7.21 (m, 3 H), 4.10 (dd, *J* = 8.5, 4.6 Hz, 1 H), 3.82-3.74 (m, 2 H), 2.67 (bs, 3 H, exchangeable with D_2O), 1.92-1.81 (m, 2 H) ppm. ^{13}C-NMR (126 MHz, $CDCl_3$): δ = 146.1, 128.7, 127.1, 125.7, 62.3, 56.6, 39.5 ppm. MS (ESI): *m/z* (%) = 152.0 (110) [M + H^+]. HRMS (MALDI): calcd. for $C_9H_{14}NO$ [M + H^+]: 152.10699; found 152.10678.

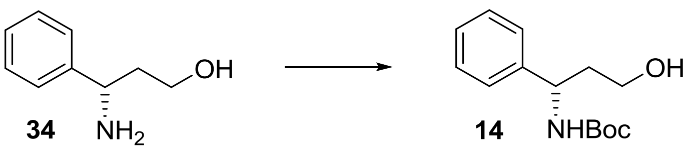

(*S*)-*tert*-Butyl (3-hydroxy-1-phenylpropyl)carbamate (14): To a solution of (*S*)-3-amino-3-phenylpropan-1-ol (**34**, 4.45 g, 29.43 mmol, 1.00 equiv) and triethylamine (4.90 mL, 35.35 mmol, 1.20 equiv) in CH_2Cl_2 (50 mL) at 0 °C was added slowly a solution of di-*tert*-butyl dicarbonate (6.42 g, 29.42 mmol, 1.00 equiv) in CH_2Cl_2 (15 mL) and stirred for 3 h at 0 °C. After stirring the solution for 2 days at room temperature the amount of solvent was reduced to 50 %, 1 M HCl (50 mL) was added and stirred for 10 min. at room temperature. The aqueous phase was extracted with CH_2Cl_2 and washed with water. The combined organic layers were dried over $MgSO_4$ and evaporated under reduced pressure. Purification by silica gel chromatography (*c*-hexane/EtOAc 3:1) gave **14** as a colourless solid (7.38 g, 29.36 mmol,

100%). R_f = 0.60 (*c*-hexane/EtOAc 1:1). ^{1}H-NMR (250 MHz, $CDCl_3$): δ = 7.34-7.19 (m, 5 H), 5.05 (broad, 1 H), 4.85 (broad, 1 H), 3.66 (dd, J = 7.4, 4.0 Hz, 2 H), 2.93 (broad, 1 H), 2.10-1.97 (m, 1 H), 1.86-1.74 (m, 1 H), 1.40 (s, 9 H) ppm. ^{13}C-NMR (63 MHz, $CDCl_3$): δ = 155.9, 142.1, 128.2, 126.8, 126.0, 79.3, 58.7, 51.7, 38.9, 28.0 ppm. MS (ESI): m/z (%) = 252.0 (110) [M + H^+]. HRMS (MALDI): calcd. for $C_{14}H_{22}NO_3$ [M + H^+]: 252.15942; found 252.15953. At this stage, an enantiomeric excess ≥ 99 % was determined by HPLC.

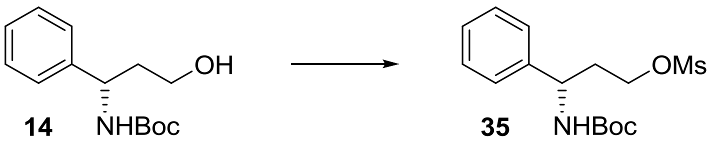

(*S*)-3-((*tert*-Butoxycarbonyl)amino)-3-phenylpropyl methanesulfonate (35): To a solution of alcohol **14** (4.16 g, 16.55 mmol, 1.00 equiv) and methanesulfonyl chloride (1.41 mL, 18.22 mmol, 1.10 equiv) in CH_2Cl_2 (50 mL) at 0 °C was added triethylamine (2.52 mL, 18.18 mmol, 1.10 equiv) over a period of 30 minutes. After stirring for 2 h at 0 °C and over night at room temperature, the solvent was evaporated and EtOAc (70 mL) was added. The reaction mixture was washed with 0.5 M H_2SO_4 (20 mL), water (2 × 50 mL) and brine (2 × 50 mL), dried over $MgSO_4$ and concentrated in vacuo. Purification by silica gel chromatography (*c*-hexane/EtOAc 7:3) gave **35** as a colourless solid (4.33 g, 13.14 mmol, 79%). R_f = 0.70 (*c*-hexane/EtOAc 1:1). ^{1}H-NMR (500 MHz, $CDCl_3$): δ = 7.28 (t, J = 7.3 Hz, 2 H), 7.23-7.19 (m, 3 H), 4.82 (broad, 1 H), 4.76 (broad, 1 H), 4.21-4.10 (m, 2 H), 2.92 (s, 3 H), 2.20-2.08 (m, 2 H), 1.34 (s, 9 H) ppm. ^{13}C-NMR (126 MHz, $CDCl_3$): δ = 155.2, 141.1, 128.9, 127.8, 126.3, 79.9, 66.9, 51.5, 37.3, 35.9, 28.3 ppm. MS (ESI): m/z (%) = 330.6 (100) [M + H^+]. HRMS (MALDI): calcd. for $C_{15}H_{23}NO_5SK$ [M + K^+]: 368.09285; found 368.09299.

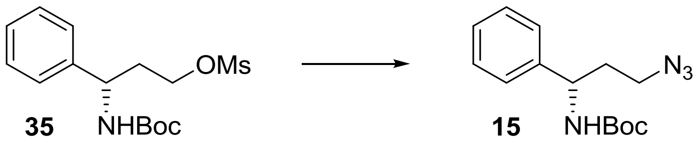

(*S*)-*tert*-Butyl (3-azido-1-phenylpropyl)carbamate (15): To a solution of mesylate **35** (4.14 g, 12.57 mmol, 1.00 equiv) in DMF (100 mL) was added carefully NaN_3 (2.45 g, 37.69 mmol, 3.00 equiv) and stirred for 5 days at room temperature. The solvent was evaporated under reduced pressure and CH_2Cl_2 (100 mL) was added to the residue. The clear solution was washed with brine (2 × 50 mL), dried over $MgSO_4$ and concentrated in vacuo. Purification by silica gel chromatography (*c*-hexane/EtOAc 6:1) gave **15** as a colourless solid (3.34 g, 12.09 mmol, 96%). R_f = 0.68 (*c*-hexane/EtOAc 3:1). ^{1}H-NMR (500 MHz, $CDCl_3$): δ = 7.28 (t, J = 7.6 Hz, 2 H), 7.23-7.19 (m, 3 H), 4.86 (broad, 1 H), 4.70 (broad, 1 H), 3.28-3.19 (m, 2 H),

1.99-1.94 (m, 2 H), 1.35 (s, 9 H) ppm. ^{13}C-NMR (126 MHz, $CDCl_3$): δ = 155.1, 141.5, 128.8, 127.6, 126.3, 79.7, 52.6, 48.5, 35.8, 28.3 ppm. Anal. calcd. for $C_{14}H_{20}N_4O_2$: C, 60.85; H, 7.30; N, 20.28; found: C, 60.85; H, 7.16; N, 20.53.

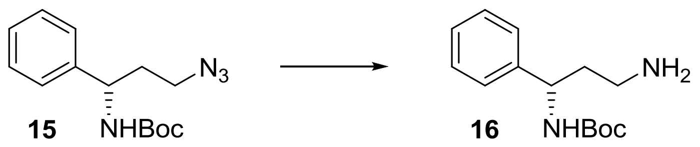

(*S*)-*tert*-Butyl (3-amino-1-phenylpropyl)carbamate (16): A solution of azide **15** (4.80 g, 17.4 mmol) in dry methanol (100 mL) was treated with palladium (0.74 g, 10% on charcoal) and stirred under a balloon of H_2 overnight. The black suspension was filtered through Celite to obtain a clear solution that was concentrated under reduced pressure. Purification by silica gel chromatography ($MeOH/CH_2Cl_2$ 2:1) gave amine **16** as a light yellow solid (3,65 g, 14,6 mmol, 84%). R_f = 0.13 ($MeOH/CH_2Cl_2$ 2:1). ^{1}H-NMR (500 MHz, $CDCl_3$): δ = 7.27 (t, J = 7.6 Hz, 2 H), 7.23-7.17 (m, 3 H), 5.43 (broad, 1 H, exchangeable with D_2O), 4.73 (broad, 1 H), 2.73-2.63 (m, 2 H), 1.90-1.75 (m, 2 H), 1.36 (s, 9 H), 1.31 (s, 2 H, exchangeable with D_2O) ppm. ^{13}C-NMR (126 MHz, $CDCl_3$): δ = 155.4, 142.7, 128.6, 127.1, 126.2, 79.3, 53.0, 40.0, 38.9, 28.4 ppm. MS (ESI): *m/z* (%) = 250.95 (100) [M + H^+]. HRMS (MALDI): calcd. for $C_{14}H_{23}N_2O_2$ [M + H^+]: 251.17540; found 251.17568.

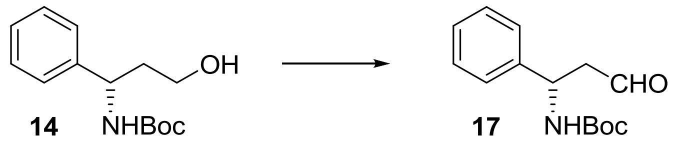

(*S*)-*tert*-Butyl (3-oxo-1-phenylpropyl)carbamate (17): To a solution of alcohol **14** (1.94 g, 7.72 mmol, 1.00 equiv) and triethylamine (4.39 mL, 31.67 mmol, 4.10 equiv) in dry CH_2Cl_2 (12.50 mL) and dry DMSO (3.13 mL) at 0 °C and under an argon atmosphere was added a suspension of the sulfur trioxide pyridine complex (2.46 g, 15.46 mmol, 2.00 equiv) and dry pyridine (1.43 mL, 17.75 mmol, 2.30 equiv) in dry DMSO (3.13 mL). After 10 minutes at 0 °C the stirring was continued for 2 h at room temperature. The reaction mixture was cooled to 0 °C and water (50 mL) was added. The aqueous phase was extracted with CH_2Cl_2 and washed with brine (2 × 50 mL). The combined organic layers were dried over $MgSO_4$ and evaporated under reduced pressure. Purification by silica gel chromatography (*c*-hexane/EtOAc 7:3) gave aldehyde **17** as a colourless solid (1.68 g, 6.74 mmol, 87%). R_f = 0.43 (*c*-hexane/EtOAc 3:1). ^{1}H-NMR (500 MHz, $CDCl_3$): δ = 9.68 (t, J = 1.6 Hz, 1 H), 7.28 (t, J = 7.5 Hz, 2 H), 7.24-7.20 (m, 3 H), 5.14 (broad, 1 H), 5.05 (broad, 1 H), 2.95-2.82 (m, 2 H), 1.35 (s, 9 H) ppm. ^{13}C-NMR (126 MHz, $CDCl_3$): δ = 200.2, 155.0, 140.9, 128.9, 127.8,

126.3, 80.0, 50.1, 49.9, 28.3 ppm. MS (ESI): *m/z* (%) = 250.6 (100) [M + H^+]. HRMS (MALDI): calcd. for $C_{14}H_{20}NO_3$ [M + H^+]: 250.14377; found 250.14386. Anal. calcd. for $C_{14}H_{19}NO_3$: C, 67.45; H, 7.68; N, 5.62; found: C, 67.30; H, 7.33; N, 5.37.

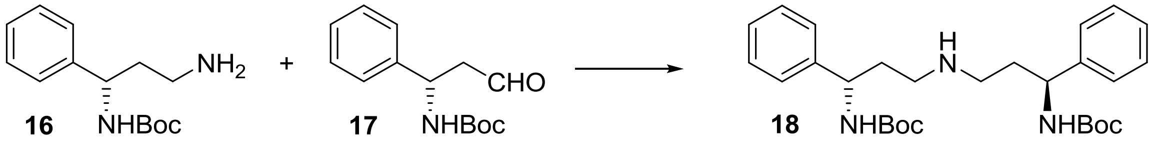

Di-*tert*-butyl ((1*S*,1'*S*)-azanediylbis(1-phenylpropane-3,1-diyl))dicarbamate (18): A solution of amine **16** (1.38 g, 5.51 mmol, 1.00 equiv) and aldehyde **17** (1.37 g, 5.50 mmol, 1.00 equiv) in dry THF (50 mL) was stirred for 2 days under an argon atmosphere at room temperature. The solvent was evaporated under reduced pressure and methanol (50 mL) was added to the residue. After stirring for 1 h at room temperature $NaBH_4$ (0.42 g, 11.10 mmol, 2.01 equiv) was added and the reaction mixture stirred at room temperature for 4 days. The solvent was removed under reduced pressure and an aqueous solution of 20% KOH (40 mL) was added to the residue. After stirring for 2 h at room temperature the aqueous phase was extracted with CH_2Cl_2 (5 × 50 mL), the combined organic layers were dried over $MgSO_4$ and evaporated under reduced pressure. Purification by silica gel chromatography (*c*-hexane/EtOAc 1:3) gave amine **18** as a colourless foam (1.55 g, 3.20 mmol, 58%). R_f = 0-0.18 (*c*-hexane/EtOAc 1:3). ^{1}H-NMR (500 MHz, $CDCl_3$): δ = 7.27 (t, *J* = 7.6 Hz, 4 H), 7.23-7.18 (m, 6 H), 5.68 (broad, 2 H), 4.71 (broad, 2 H), 2.55-2.47 (m, 4 H), 1.94-1.79 (m, 4 H), 1.71 (broad, 1 H), 1.37 (s, 18 H) ppm. ^{13}C-NMR (126 MHz, $CDCl_3$): δ = 155.4, 142.6, 128.5, 127.1, 126.2, 79.3, 53.4, 46.4, 36.4, 28.4 ppm. MS (ESI): *m/z* (%) = 484.1 (110) [M + H^+]. HRMS (MALDI): calcd. for $C_{28}H_{42}N_3O_4$ [M + H^+]: 484.31698; found 484.31618.

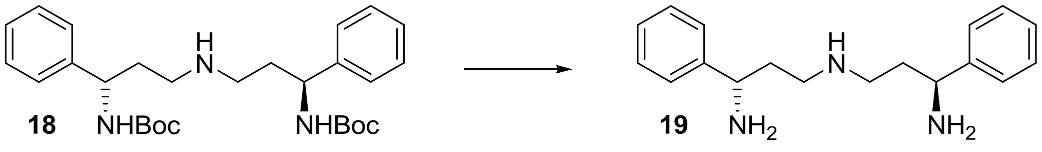

(*S*)-*N*^{1}-((*S*)-3-Amino-3-phenylpropyl)-3-phenylpropan-1,3-diamine (19): To a solution of Boc-derivative **18** (1.49 g, 3.08 mmol, 1.00 equiv) in CH_2Cl_2 (80 mL) was added TFA (2.37 mL, 30.76 mmol, 10.00 equiv) and stirred for 4 days at room temperature. The reaction mixture was heated to reflux for 23 h and then cooled to room temperature. The solution was washed with 8 M KOH (50 mL) and extracted with CH_2Cl_2 (5 × 50 mL). The combined organic layers were dried over $MgSO_4$ and evaporated under reduced pressure to give triamine **19** as a yellow oil (0.87 g, 3.07 mmol, 100%). R_f = 0-0.15 ($MeOH/CH_2Cl_2$ 2:1). ^{1}H-NMR (500 MHz, $CDCl_3$): δ = 7.29-7.22 (m, 8 H), 7.20-7.17 (m, 2 H), 3.95 (t, *J* = 6.9 Hz, 2

H), 2.67-2.59 (m, 4 H), 2.50 (broad, 5 H), 1.83 (q, *J* = 6.7 Hz, 4 H) ppm. ^{13}C-NMR (126 MHz, $CDCl_3$): δ = 145.8, 128.5, 127.0, 126.0, 54.9, 47.2, 37.8 ppm. MS (ESI): *m/z* (%) = 283.9 (110) [M + H^+]. HRMS (MALDI): calcd. for $C_{18}H_{26}N_3$ [M + H^+]: 284.21212; found 284.21235.

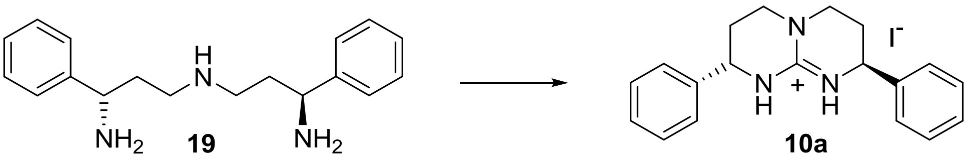

Hydroiodide salt of (2*S*,8*S*)-2,8-Diphenyl-2,3,4,6,7,8-hexahydro-1*H*-pyrimido[1,2-a]pyrimidine (10a): To a solution of triamine **19** (0.83 g, 2.93 mmol, 1.00 equiv) in nitromethane (20 mL) under an argon atmosphere was added dimethyl trithiocarbonate (0.42 mL, 3.81 mmol, 1.30 equiv) as a solution in nitromethane (3 mL) dropwise over a period of 1 h. The yellow solution was heated to reflux for 2 h and then allowed to cool to room temperature. Acetic acid (0.67 mL, 11.72 mmol, 4.00 equiv) and methyl iodide (0.37 mL, 5.86 mmol, 2.00 equiv) were added. The reaction mixture was heated to reflux for 3 h and then left stirring at room temperature under argon overnight. The solvent was evaporated under reduced pressure, H_2O (20 mL) was added to the residue and then extracted witch CH_2Cl_2 (5 × 40 mL). The combined organic layers were led through silica and were eluted with (*c*-hexane/EtOAc 1:1) to remove an excess of reagents. Elution with (MeOH/CH_2Cl_2 2:1) gave the hydroiodide **10a** as light red solid (0.82 g, 1.96 mmol, 67%). ^{1}H-NMR (500 MHz, DMSO-d_6): δ = 7.89 (s, 2 H, exchangeable with D_2O, NH), 7.44 (t, *J* = 7.6 Hz, 4 H), 7.38-7.34 (m, 6 H), 4.70 (t, *J* = 5.0 Hz, 2 H), 3.47 (m, 2 H), 3.24 (m, 2 H), 2.26 (m, 2 H), 2.00 (m, 2 H) ppm. ^{13}C-NMR (126 MHz, DMSO-d_6): δ = 150.9, 141.2, 128.7, 127.9, 126.2, 51.7, 44.6, 28.5 ppm. MS (ESI): *m/z* (%) = 292.1 (36000000) [M + H^+]. HRMS (MALDI): calcd. for $C_{19}H_{22}N_3$ [M + H^+]: 292.18082; found 292.18073.

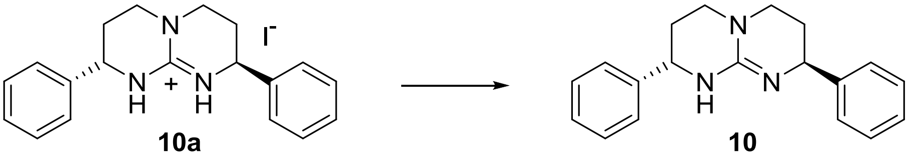

Generation of the free guanidine 10: A solution of the hydroiodide **10a** (0.41 g, 0.98 mmol, 1.00 equiv.) in CH_2Cl_2 (50 mL) was shaken with aqueous NaOH (20 M, 70 mL) and then washed with water. The combined organic layers were dried over $MgSO_4$ and then concentrated under reduced pressure to give the free guanidine **10** as a colourless foam (0.27 g, 0.93 mmol, 95%). $[\alpha]_D^{20}$ = +6.21 ° (c: 0.07, MeOH). R_f = 0.80 (MeOH/CH_2Cl_2 2:1). ^{1}H-NMR (500 MHz, DMSO-d_6): δ = 10.13 (broad, 1 H, NH), 7.37 (t, *J* = 7.6 Hz, 4 H), 7.32-7.29

(m, 6 H), 4.48 (t, J = 4.7 Hz, 2 H), 3.24 (m, 2 H), 3.03 (m, 2 H), 2.14 (m, 2 H), 1.94 (m, 2 H) ppm. ^{13}C-NMR (126 MHz, DMSO-d_6): δ = 150.7, 141.9, 128.4, 127.3, 126.1, 50.6, 43.4, 28.1 ppm.

General procedures for the reaction of anthrone derivatives 20 or 21 with *N*-substituted maleimides 22–24. All reactions were run in closed 50 mL polyethylene vessels. To a solution of anthrone (0.15–0.60 mmol) in CH_2Cl_2 or other solvents (abs., 8 mL) at −40 °C, the catalyst **10** (0.1 equiv) was added. After stirring for 15 minutes, maleimide was added to the mixture in one portion. The solution was allowed to warm up to −15 °C overnight and stirred for two more days at this temperature. After warming up to room temperature the reaction mixture was purified by flash column chromatography (*c*-hexane/EtOAc 10:1) to afford the different products as crystalline solids.

The racemic Diels–Alder products *rac*-**25**–*rac*-**27** were prepared with triethylamine (1.0 equiv) instead of guanidine **10**. After stirring for 24 hours at room temperature the reaction mixture was also purified by flash column chromatography (*c*-hexane/EtOAc 10:1).

The racemic retro-aldol products *rac*-**28**–*rac*-**31** were prepared with triethylamine (1.0 equiv.) instead of guanidine in the presence of a small amount of $LiClO_4$ acting as Lewis acid. After stirring for 7 days at room temperature the reaction mixture was also purified by flash column chromatography (*c*-hexane/EtOAc 10:1).

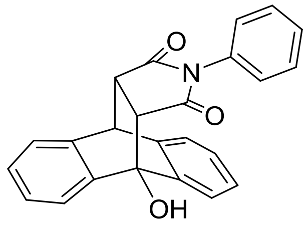

25

rac-**25**: R_f = 0.24 (*c*-hexane/EtOAc 4:1). ^{1}H-NMR (500 MHz, $CDCl_3$): δ = 7.75 (d, J = 7.3 Hz, 1 H), 7.58 (d, J = 7.3 Hz, 1 H), 7.43 (d, J = 7.3 Hz, 1 H), 7.36-7.30 (m, 6 H), 7.26-7.23 (m, 2 H), 6.51-6.48 (m, 2 H), 4.85 (d, J = 3.6 Hz, 1 H), 4.56 (s, 1 H), 3.51 (dd, J = 8.6, 3.5 Hz, 1 H), 3.29 (d, J = 8.6 Hz, 1 H) ppm. MS (ESI): m/z (%) = 368.15 (40000) [M + H^+]. HRMS (MALDI): calcd. for $C_{24}H_{18}NO_3$ [M + H^+]: 368.12812; found 368.12834.

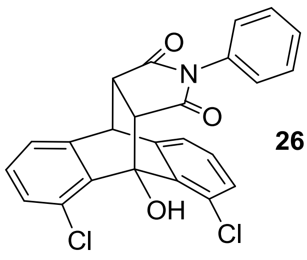

26

rac-**26**: R_f = 0.17 (*c*-hexane/EtOAc 4:1). ^{1}H-NMR (500 MHz, $CDCl_3$): δ = 7.37-7.28 (m, 6 H), 7.24 (dd, *J* = 7.3, 1.1 Hz, 1 H), 7.19-7.15 (m, 2 H), 6.63-6.59 (m, 2 H), 5.09 (s, 1 H), 4.77 (d, *J* = 3.4 Hz, 1 H), 3.52 (d, *J* = 8.9 Hz, 1 H), 3.45 (dd, *J* = 8.9, 3.4 Hz, 1 H) ppm. MS (ESI): *m/z* (%) = 436.02 (70000) [M + H^+]. HRMS (MALDI): calcd. for $C_{24}H_{15}Cl_2NO_3K$ [M + K^+]: 474.00606; found 474.00618.

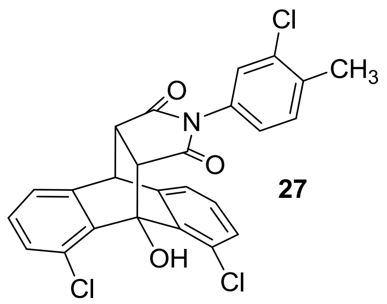

27

rac-**27**: R_f = 0.24 (*c*-hexane/EtOAc 4:1). ^{1}H-NMR (500 MHz, $CDCl_3$): δ = 7.31-7.28 (m, 3 H), 7.24 (dd, *J* = 7.6, 1.1 Hz, 1 H), 7.20-7.15 (m, 3 H), 6.60 (d, *J* = 2.0 Hz, 1 H), 6.42 (dd, *J* = 8.2, 2.0 Hz, 1 H), 5.06 (s, 1 H), 4.76 (d, *J* = 3.4 Hz, 1 H), 3.51 (d, *J* = 8.8 Hz, 1 H), 3.44 (dd, *J* = 8.8, 3.4 Hz, 1 H), 2.33 (s, 3 H) ppm. MS (ESI): *m/z* (%) = 486.00 (32000) [M + H^+]. HRMS (MALDI): calcd. for $C_{25}H_{16}Cl_3NO_3K$ [M + K^+]: 521.92873; found 521.98331.

28

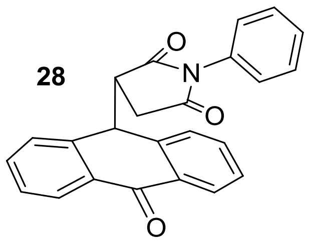

rac-**28**: R_f = 0.13 (*c*-hexane/EtOAc 4:1). ^{1}H-NMR (500 MHz, $CDCl_3$): δ = 8.39 (dd, *J* = 7.7, 1.2 Hz, 1 H), 8.35 (dd, *J* = 7.7, 1.2 Hz, 1 H), 7.71 (td, *J* = 7.6, 1.3 Hz, 1 H), 7.67 (d, *J* = 7.0 Hz, 1 H), 7.61-7.52 (m, 4 H), 7.49-7.46 (m, 2 H), 7.43-7.40 (m, 1 H), 7.11-7.09 (m, 2 H), 5.28 (d, *J* = 3.1 Hz, 1 H), 3.66-3.62 (m, 1 H), 2.42 (dd, *J* = 18.7, 9.5 Hz, 1 H), 2.09 (dd, *J* = 18.7, 4.9 Hz, 1 H) ppm. MS (ESI): *m/z* (%) = 368.15 (55000) [M + H^+]. HRMS (MALDI): calcd. for $C_{24}H_{18}NO_3$ [M + H^+]: 368.12812; found 368.12833.

29

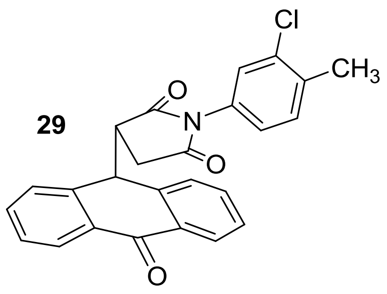

rac-**29**: R_f = 0.13 (*c*-hexane/EtOAc 4:1). ^{1}H-NMR (500 MHz, $CDCl_3$): δ = 8.38 (dd, *J* = 7.3, 1.5 Hz, 1 H), 8.34 (dd, *J* = 7.9, 1.5 Hz, 1 H), 7.70 (td, *J* = 7.7, 1.3 Hz, 1 H), 7.65 (d, *J* = 7.7 Hz, 1 H), 7.61-7.52 (m, 3 H), 7.49 (dd, *J* = 7.3, 1.6 Hz, 1 H), 7.31 (d, *J* = 8.0 Hz, 1 H), 7.10 (d, *J* = 2.1 Hz, 1 H), 6.90 (dd, *J* = 8.0, 2.1 Hz, 1 H), 5.24 (d, *J* = 3.1 Hz, 1 H), 3.64-3.60 (m, 1 H), 2.43-2.38 (m, 1 H), 2.40 (s, 3 H), 2.10-2.04 (m, 1 H) ppm. MS (ESI): *m/z* (%) = 414.03 (7500) [M - H^+]. HRMS (MALDI): calcd. for $C_{25}H_{18}ClNO_3K$ [M + K^+]: 454.06068; found 454.06100.

30

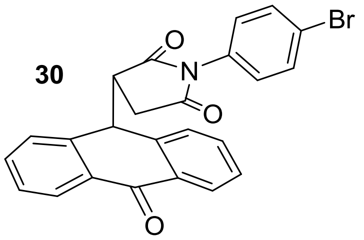

rac-**30**: R_f = 0.18 (*c*-hexane/EtOAc 4:1). ^{1}H-NMR (500 MHz, $CDCl_3$): δ = 8.39-8.36 (m, 1 H), 8.34 (dd, *J* = 7.9, 1.2 Hz, 1 H), 7.70 (td, *J* = 7.8, 1.3 Hz, 1 H), 7.65 (d, *J* = 7.8 Hz, 1 H), 7.60-7.53 (m, 5 H), 7.49-7.46 (m, 1 H), 6.99 (dt, *J* = 8.7, 2.4 Hz, 2 H), 5.25 (d, *J* = 3.1 Hz, 1 H), 3.64-3.61 (m, 1 H), 2.42 (dd, *J* = 18.8, 9.4 Hz, 1 H), 2.08 (dd, *J* = 18.7, 4.9 Hz, 1 H) ppm. MS (ESI): *m/z* (%) = 445.98 (19000) [M + H^+]. HRMS (MALDI): calcd. for $C_{24}H_{16}BrNO_3K$ [M + K^+]: 483.99451; found 483.99513.

31

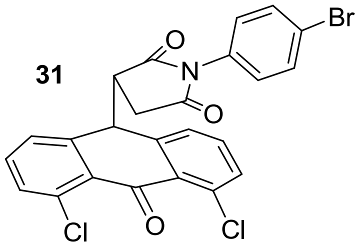

rac-**31**: R_f = 0.07 (*c*-hexane/EtOAc 4:1). ^{1}H-NMR (500 MHz, $CDCl_3$): δ = 7.58 (dt, *J* = 8.7, 2.4 Hz, 2 H), 7.55 (dd, *J* = 8.0, 1.2 Hz, 1 H), 7.53-7.49 (m, 3 H), 7.41 (t, *J* = 8.0 Hz, 1 H), 7.36 (dd, *J* = 8.0, 0.7 Hz, 1 H), 7.01 (dt, *J* = 8.7, 2.4 Hz, 2 H), 5.15 (d, *J* = 3.2 Hz, 1 H), 3.60-3.56 (m, 1 H), 2.58 (dd, *J* = 18.5, 9.4 Hz, 1 H), 2.28 (dd, *J* = 18.6, 5.8 Hz, 1 H) ppm. MS (ESI): *m/z* (%) = 515.94 (12000) [M + H^+]. HRMS (MALDI): calcd. for $C_{24}H_{14}BrCl_2NO_3K$ [M + K^+]: 551.91657; found 551.91693.

Determination of absolute configurations by chemical correlation

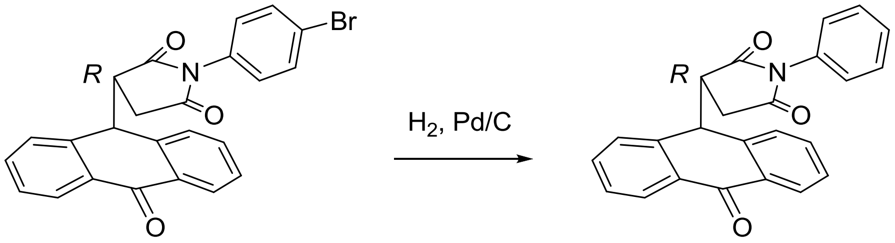

30 28

A solution of bromo compound **30** (14 mg, 0.03 mmol, 1.00 equiv, *R* isomer dominates with 95% ee) in dry methanol (20 mL) was treated with a small amount of palladium (10% on charcoal) and stirred under a balloon of H_2 for 1 hour. The black suspension was filtered through Celite to obtain a clear solution that was concentrated under reduced pressure. The raw material was used without further purification for analysis by chiral HPLC: *R* isomer dominates with 90% ee.

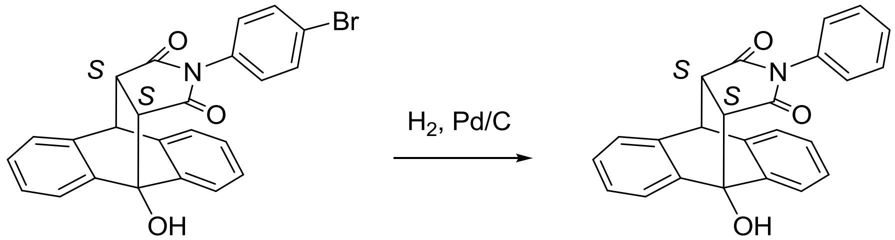

32 25

A solution of bromo compound **32** (23 mg, 0.05 mmol, 1.00 equiv, *S,S* isomer dominates with 40% ee) in dry methanol (10 mL) was treated with a small amount of palladium (10% on charcoal) and stirred under a balloon of H_2 for 22 hours. The black suspension was filtered through Celite to obtain a clear solution that was concentrated under reduced pressure. The raw material was used without further purification for analysis by chiral HPLC: *S,S* isomer dominates with 35% ee.

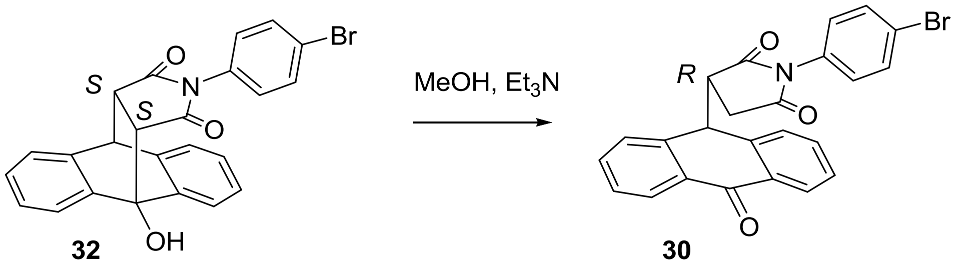

32 30

To a solution of bromo compound **32** (48 mg, 0.11 mmol, 1.00 equiv, *S,S* isomer dominates with 40% ee) in methanol (20 mL) was added triethylamine (0.01 mL, 0.07 mmol, 0.64 equiv) and stirred for 1 hour at room temperature. The clear solution was concentrated under reduced pressure and filtered through silica gel (EtOAc) in order to remove triethylamine. The raw material was used without further purification for analysis by chiral HPLC: *R* isomer dominates with 31% ee. Prolonged reaction times may lead to complete racemization of **30**.

Chromatogram of racemic *N*-Boc-β-phenylalaninol *rac*-**14**

(Chiralpak IA; *n*-hexane/iPrOH 10/1; 0.8 mL/min)

Racemic:

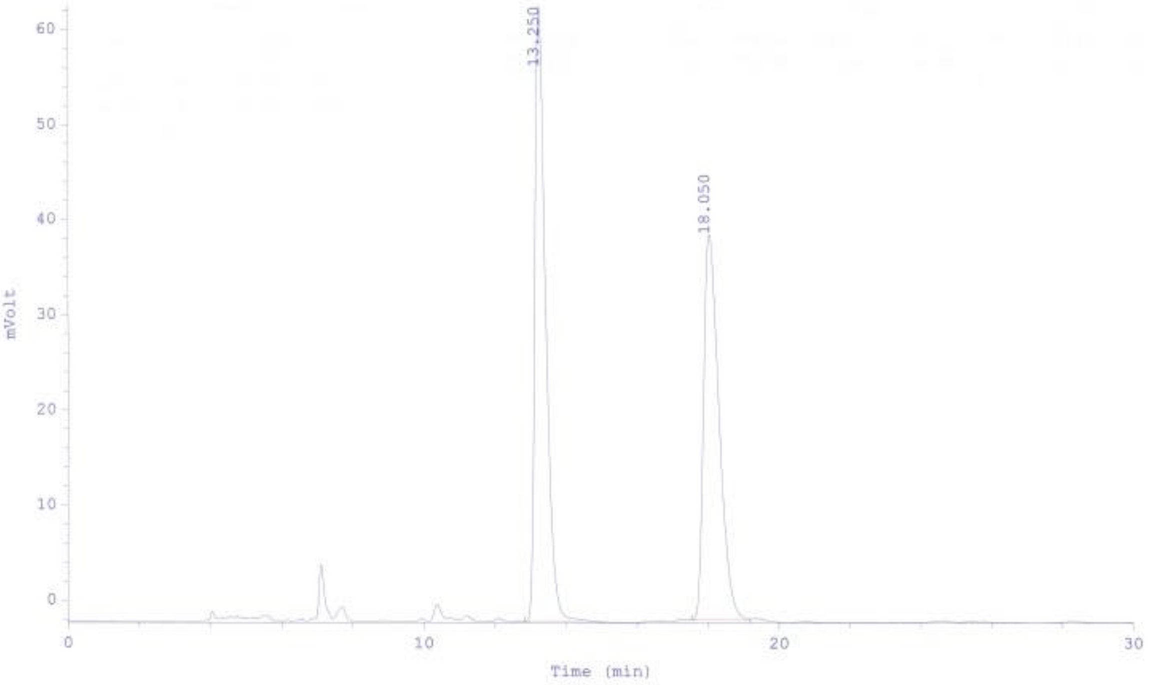

Chromatogram of *N*-Boc-β-phenylalaninol **14** (ee > 99) (conditions as shown above):

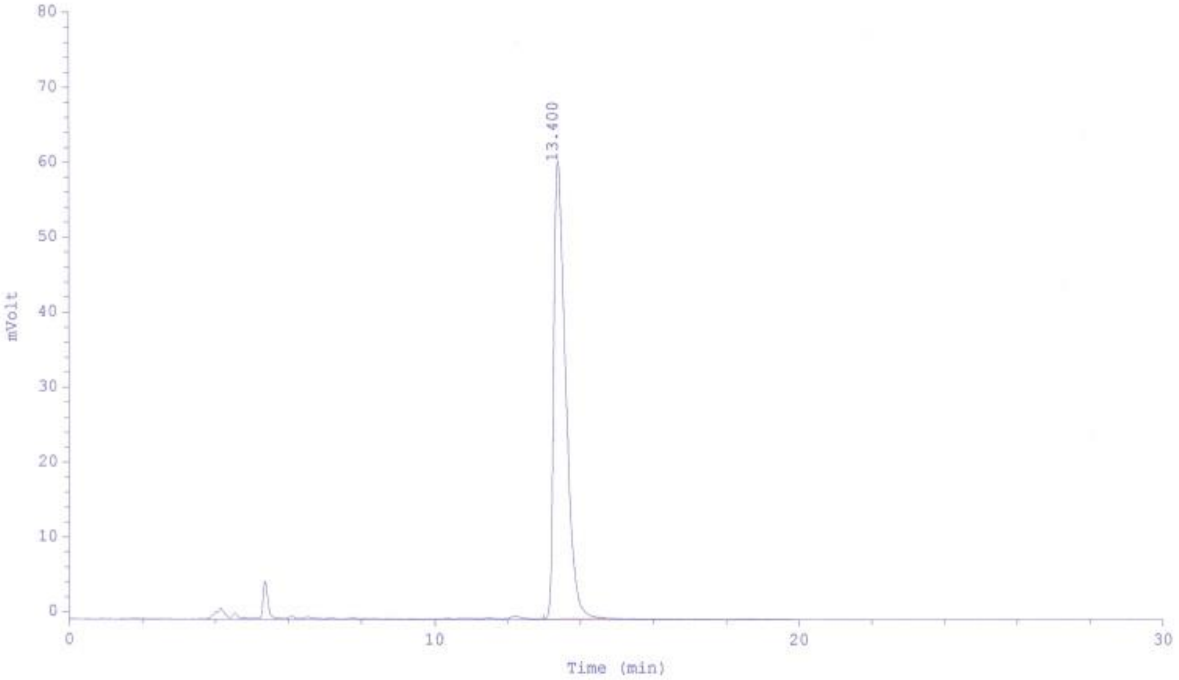

Chromatogram of racemic Diels–Alder adduct *rac*-**25** (anthrone **20** + *N*-phenyl maleimide **22**) (Chiralpak IA; *n*-hexane/iPrOH 10/3 + 20% CH_2Cl_2; 0.7 mL/min)

Racemic:

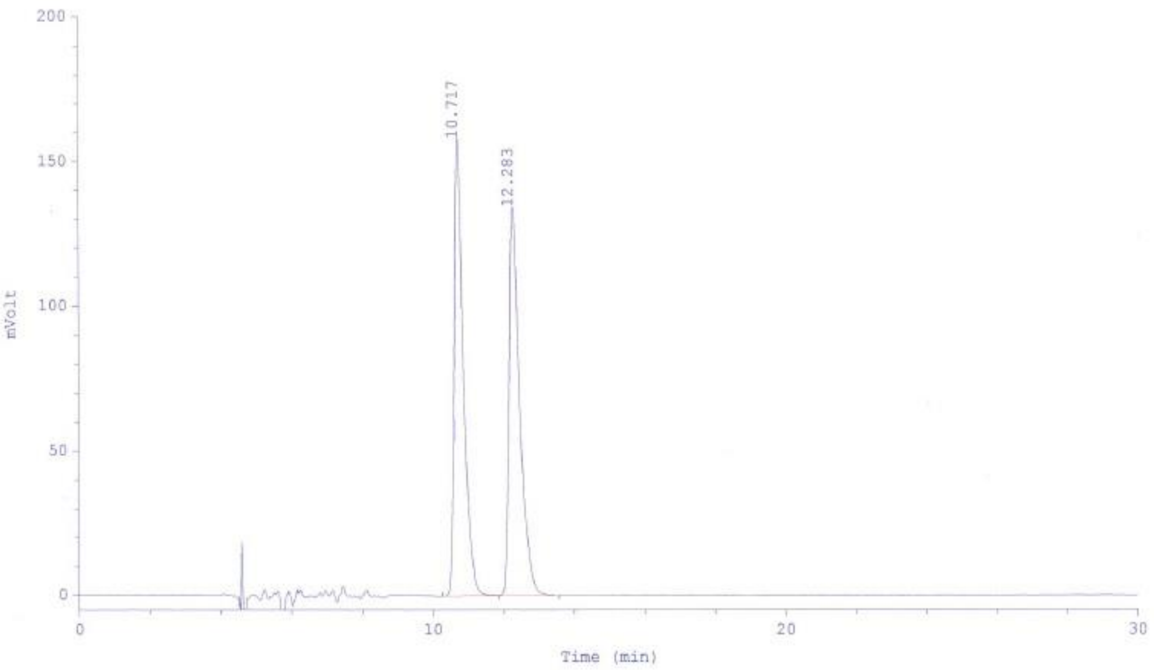

*Catalyzed by guanidine **10**:*

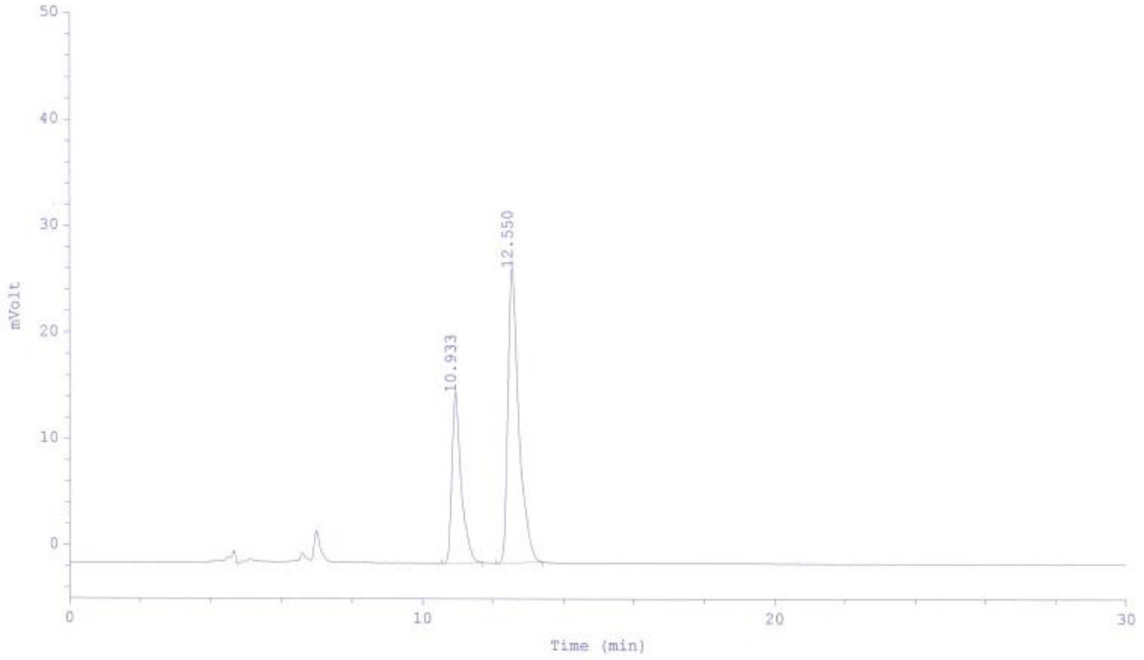

Chromatogram of racemic Diels–Alder adduct *rac*-**26** (chloro anthrone **21** + *N*-phenyl maleimide **22**) (Chiralpak IA; *n*-hexane/iPrOH 10/3 + 20% CH_2Cl_2; 0.7 mL/min)

Racemic:

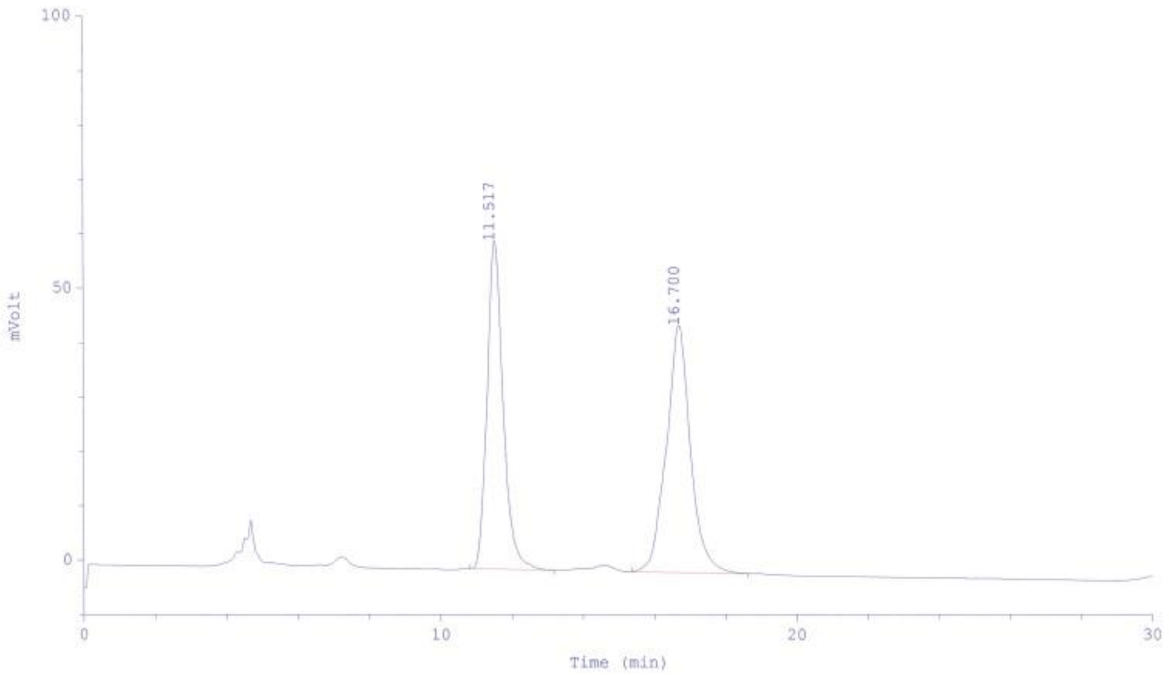

Catalyzed by guanidine ***10****:*

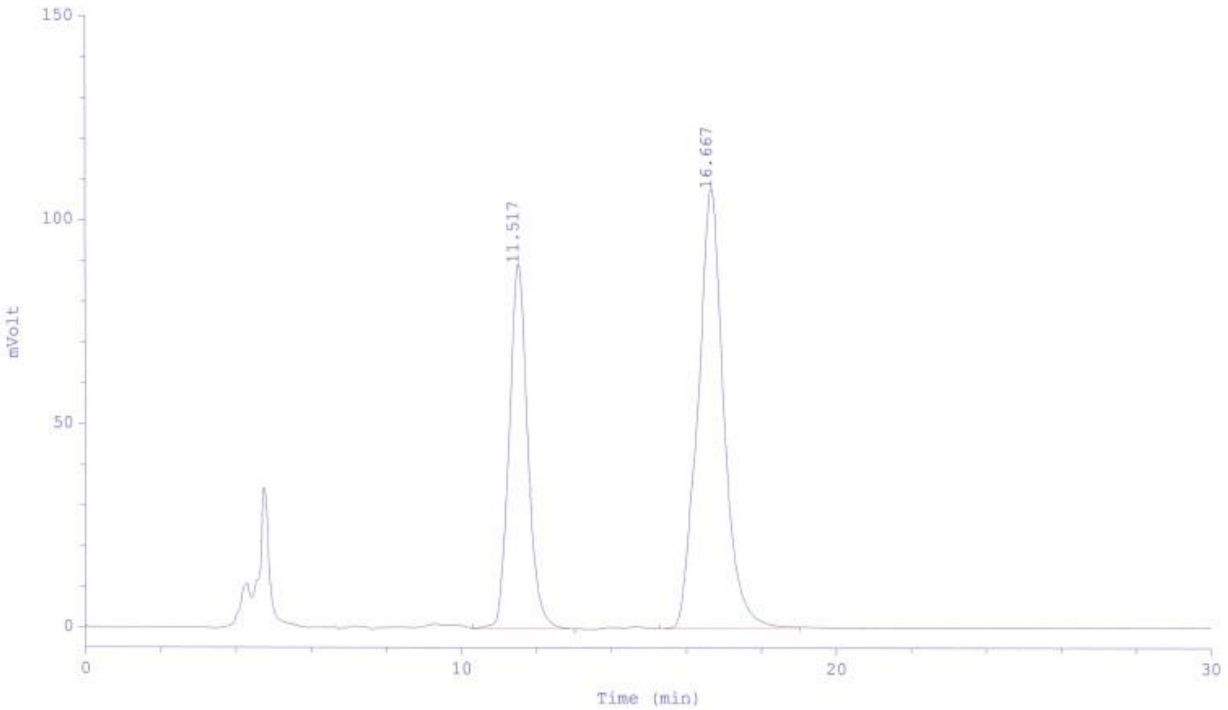

Chromatogram of racemic Diels–Alder adduct *rac*-**27** (chloro anthrone **21** + *N*-3-chloro,4-methylphenyl maleimide **23**) (Chiralpak IA; *n*-hexane/iPrOH 10/3 + 20% CH_2Cl_2; 0.7 mL/min)

Racemic:

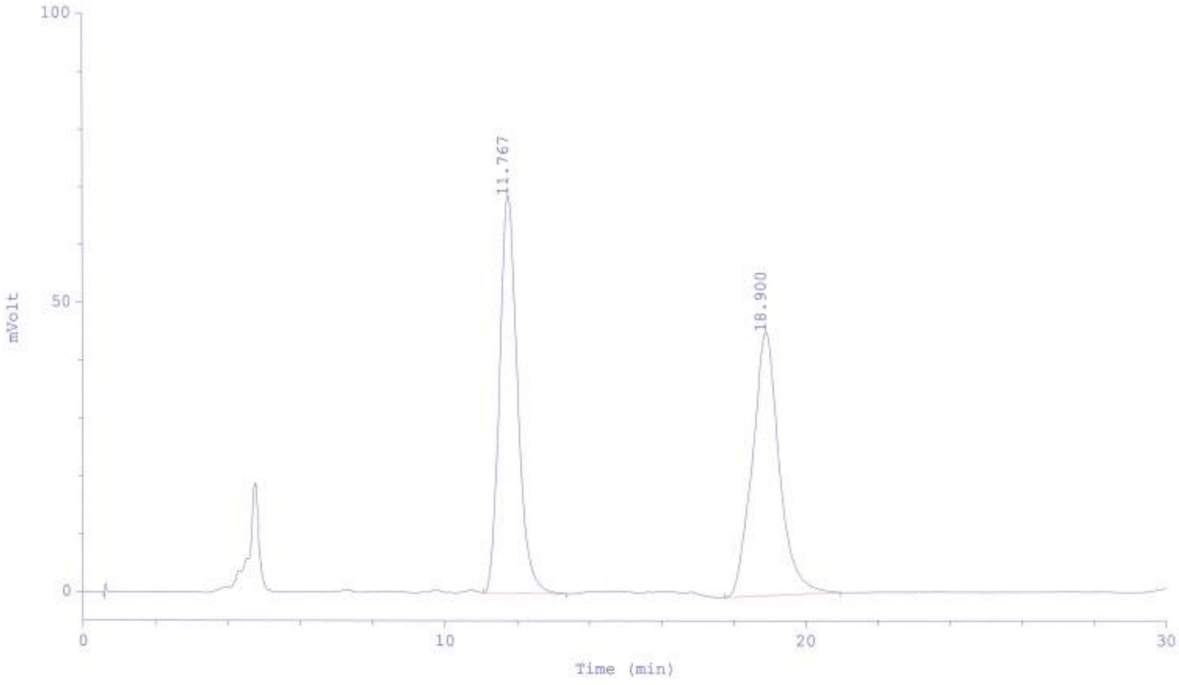

*Catalyzed by guanidine **10**:*

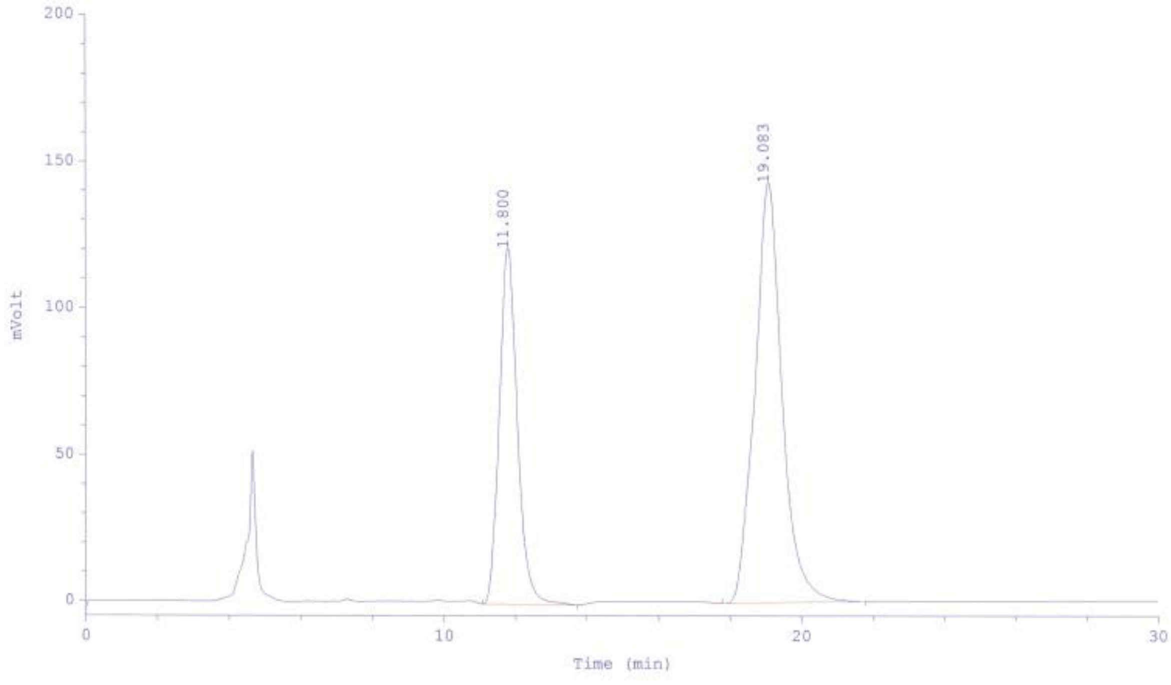

Chromatogram of racemic retro-aldol product *rac*-**28** (anthrone **20** + *N*-phenyl maleimide **22**) (Chiralpak IA; *n*-hexane/iPrOH 10/3 + 20% CH_2Cl_2; 0.7 mL/min)

Racemic:

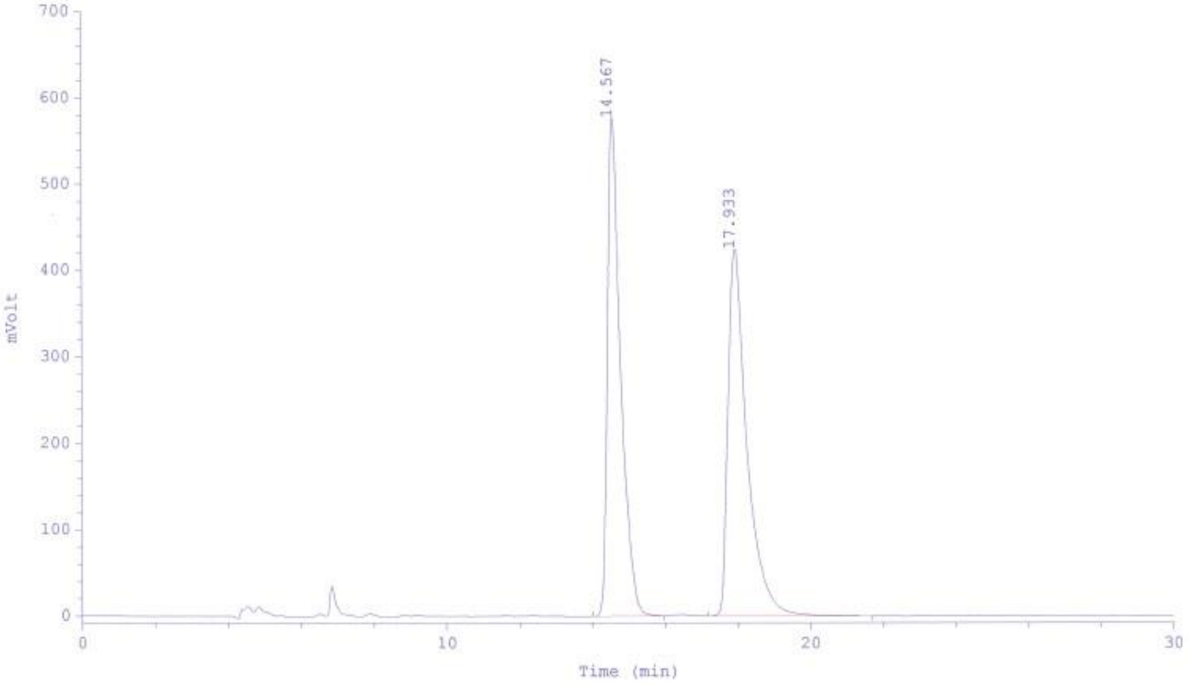

*Catalyzed by guanidine **10**:*

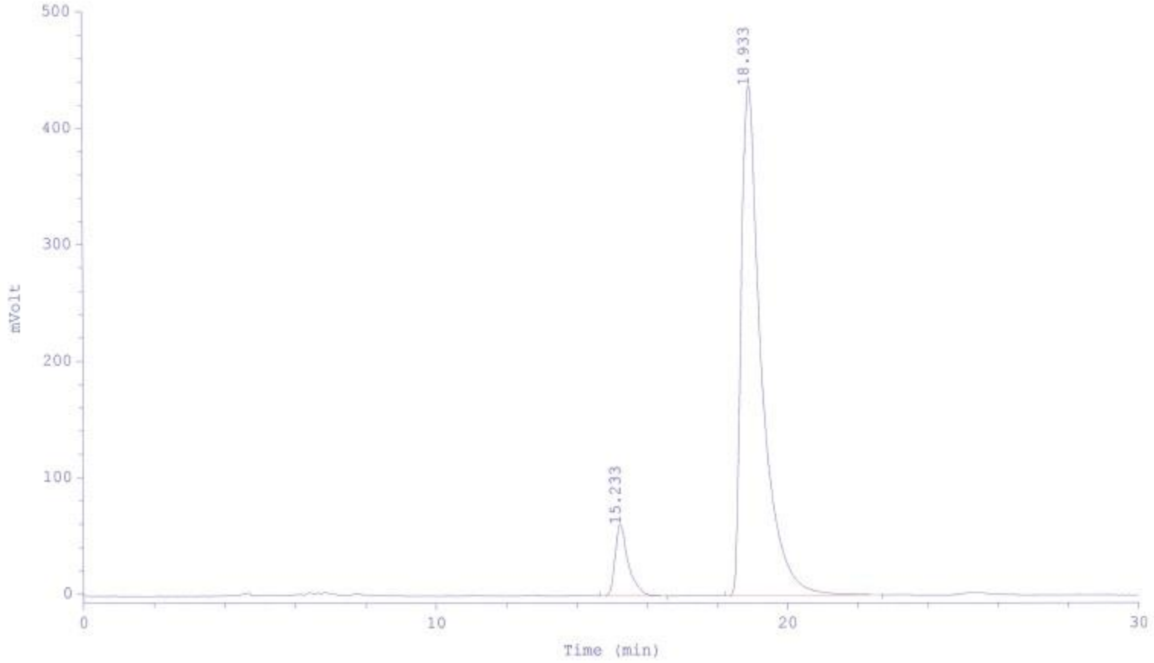

28 *recrystallized:*

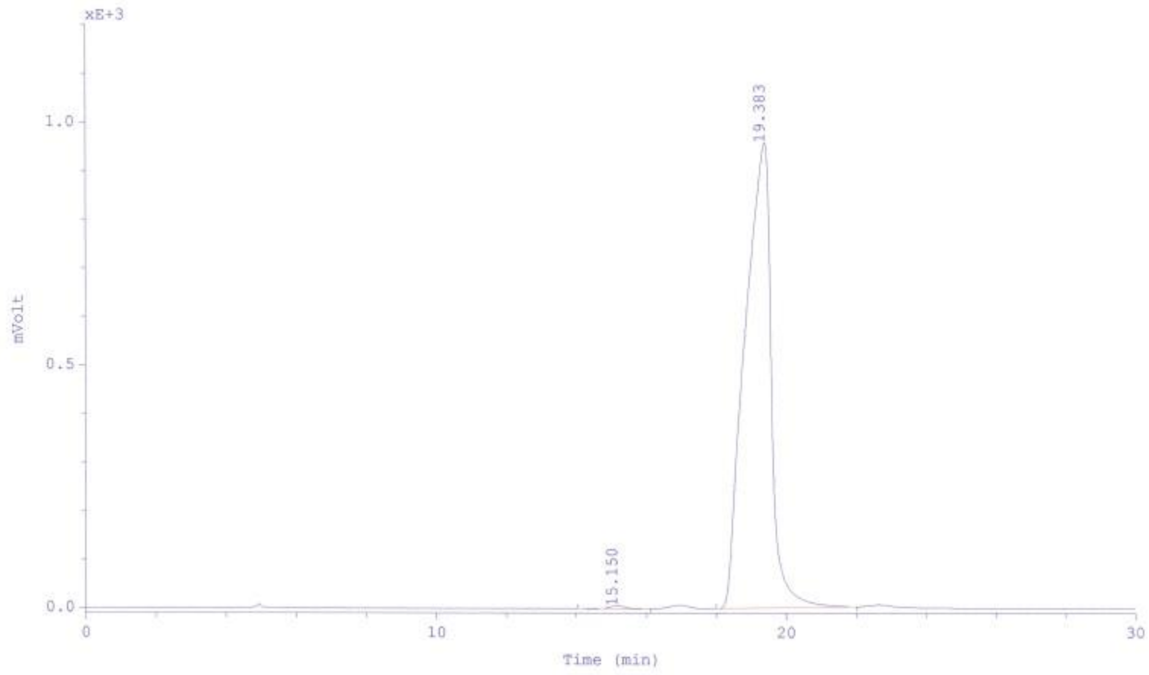

Chromatogram of racemic retro-aldol product *rac*-**29** (anthrone **20** + *N*-3-chloro,4-methylphenyl maleimide **23**) (Chiralpak IA; *n*-hexane/iPrOH 10/3 + 20% CH_2Cl_2; 0.7 mL/min)

Racemic:

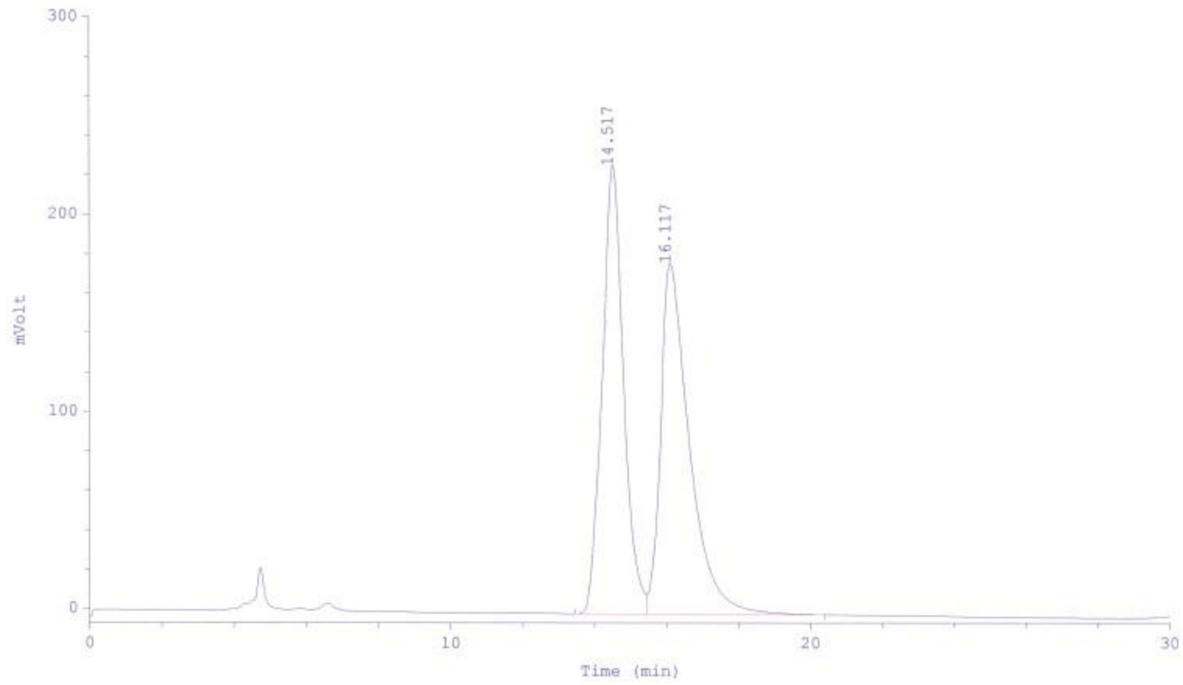

*Catalyzed by guanidine **10**:*

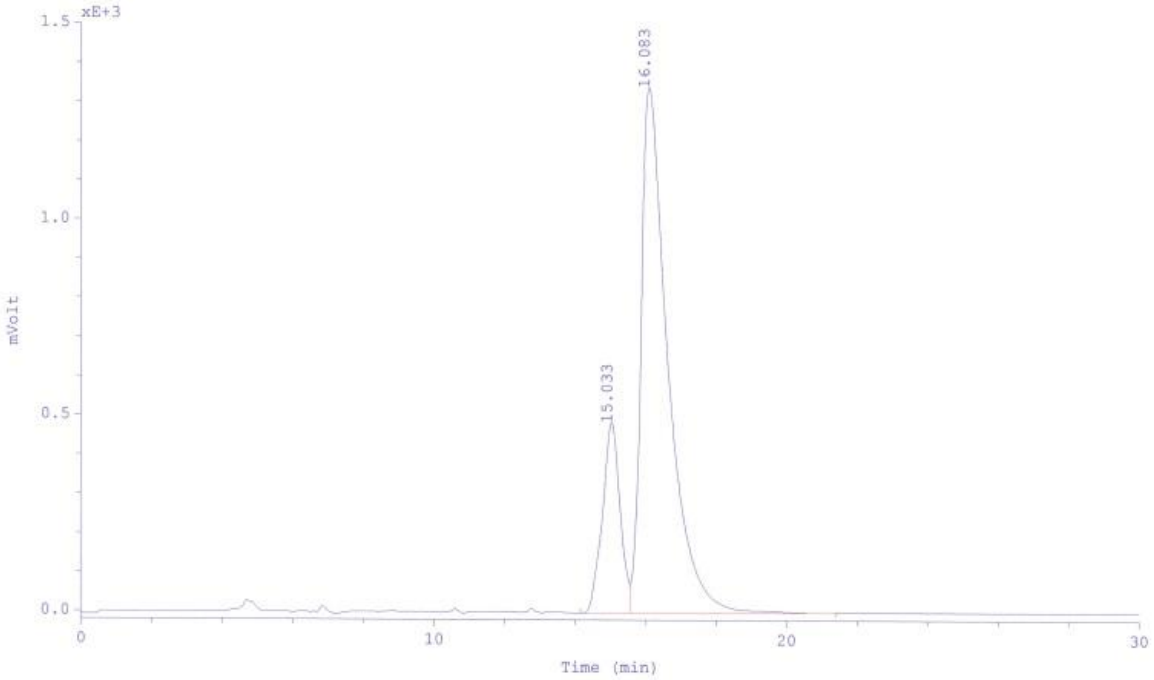

***29** recrystallized:*

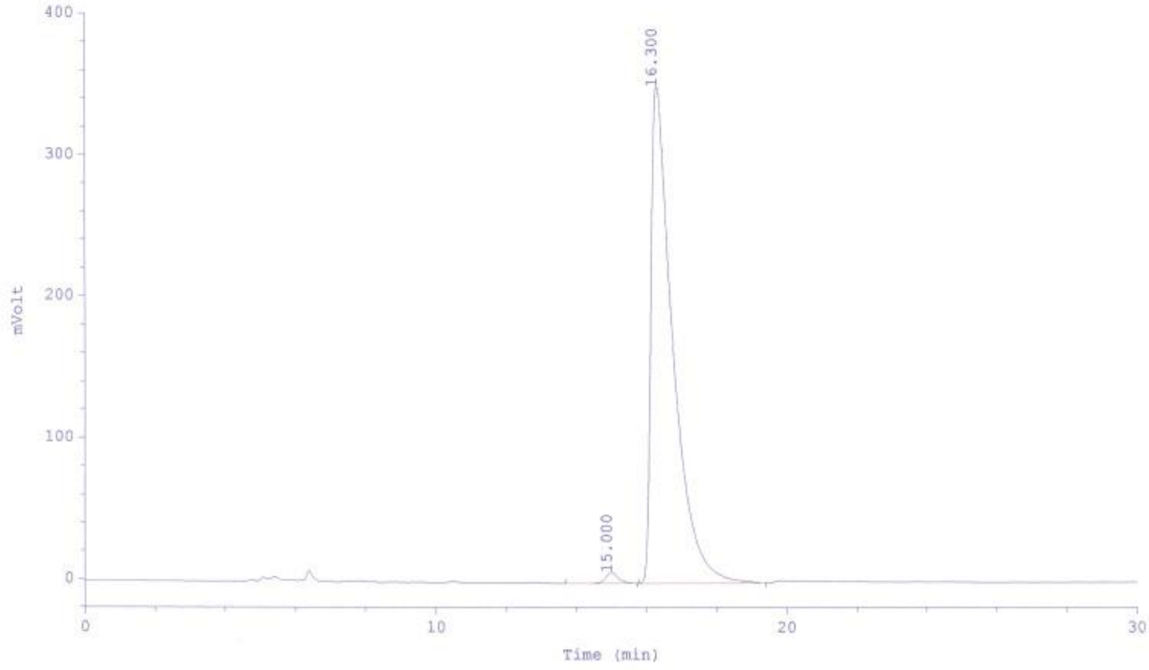

Chromatogram of racemic retro-aldol product *rac*-**30** (anthrone **20** + *N*-4-bromophenyl maleimide **24**) (Chiralpak IA; *n*-hexane/iPrOH 10/3 + 20% CH_2Cl_2; 0.7 mL/min)

Racemic:

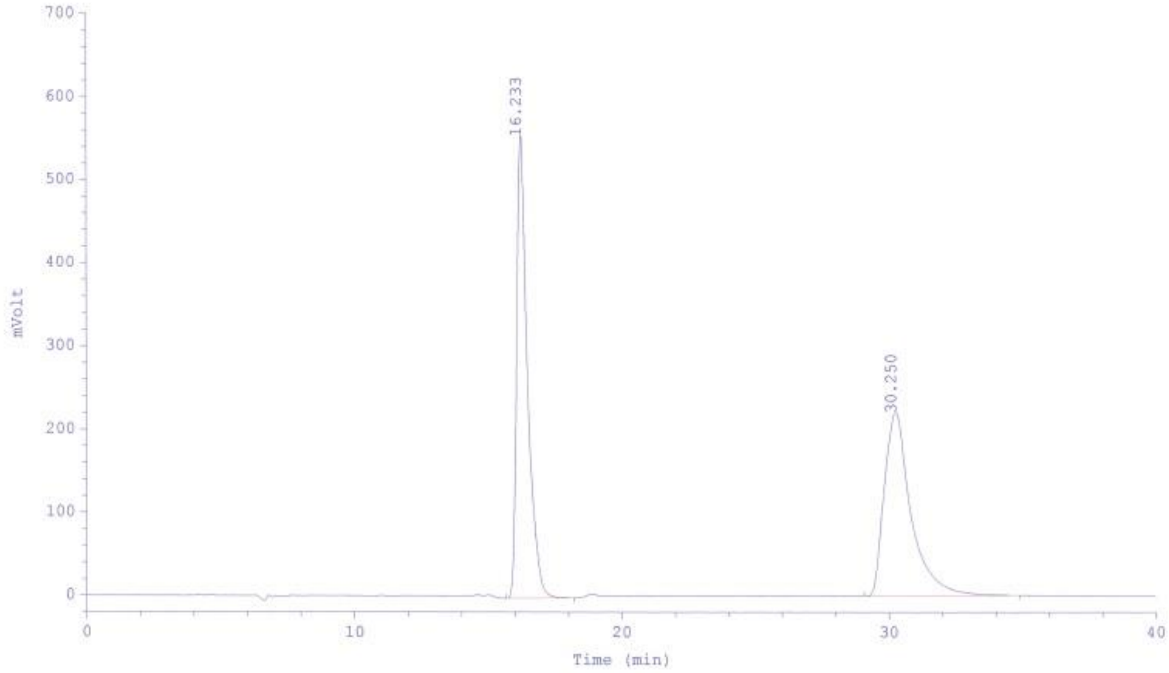

*Catalyzed by guanidine **10**:*

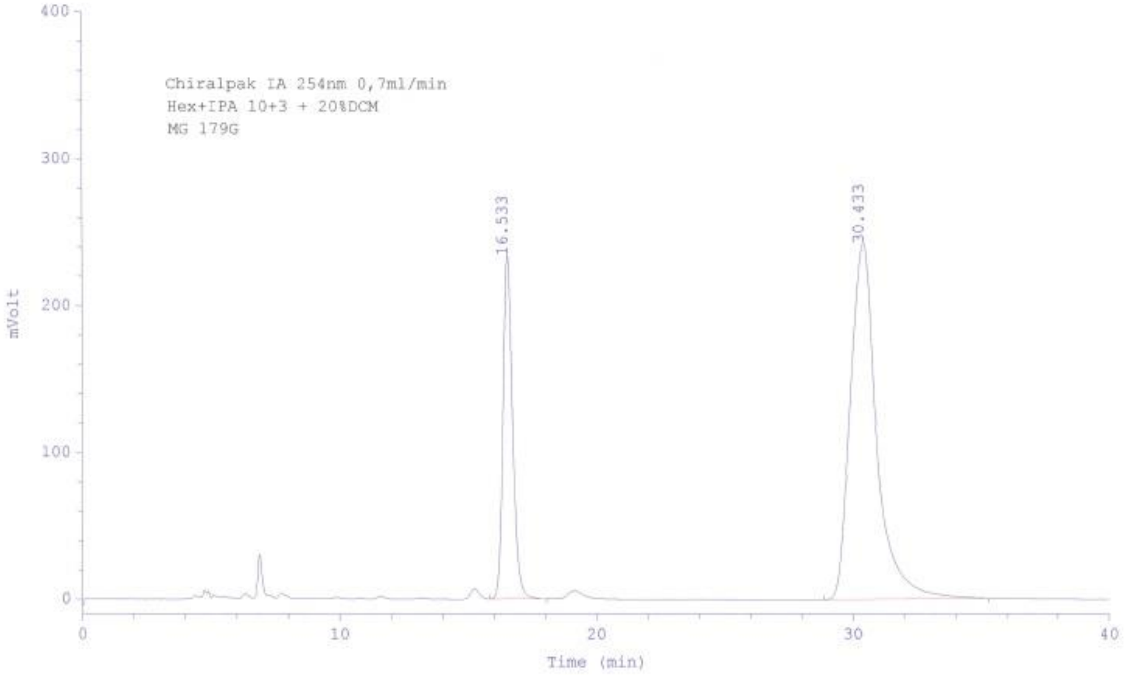

30 *recrystallized:*

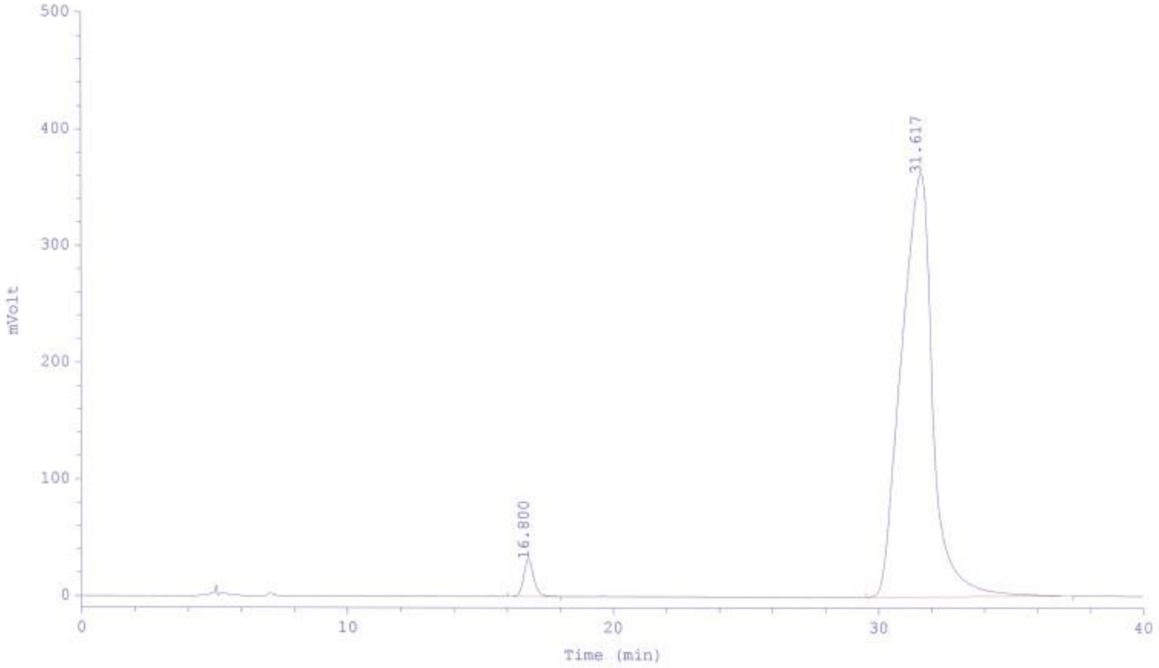

Chromatogram of racemic retro-aldol product *rac*-**31** (chloro anthrone **21** + *N*-4-bromophenyl maleimide **24**) (Chiralpak IA; *n*-hexane/iPrOH 10/3 + 20% CH_2Cl_2; 0.7 mL/min)

Racemic:

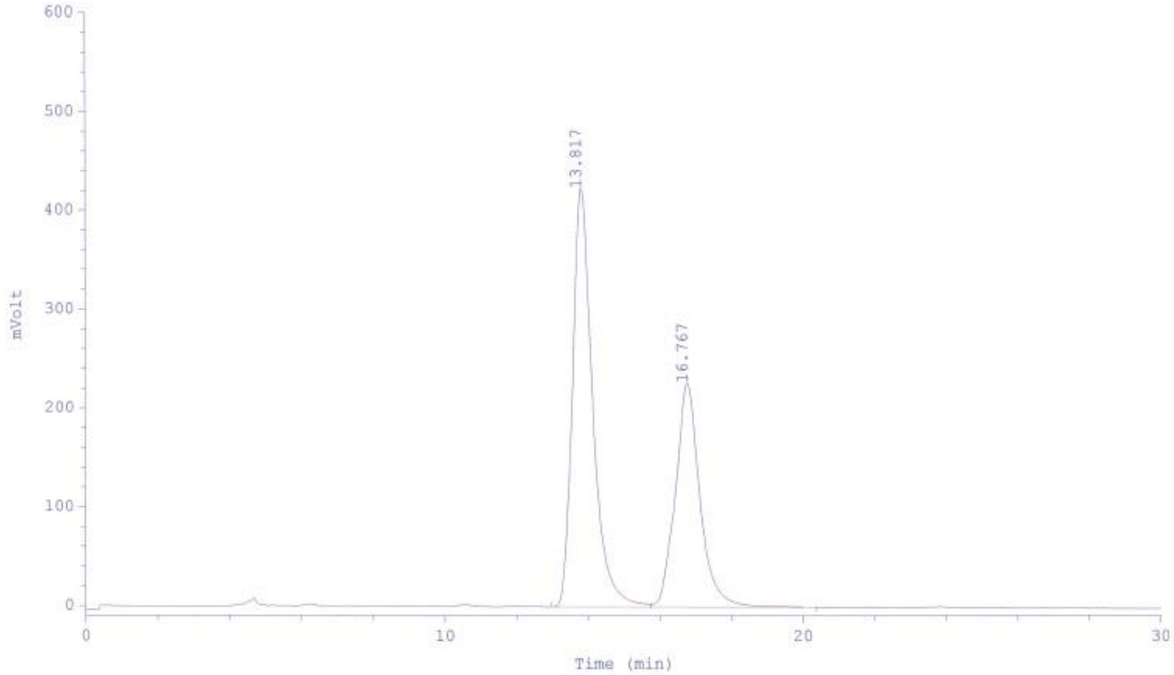

*Catalyzed by guanidine **10**:*

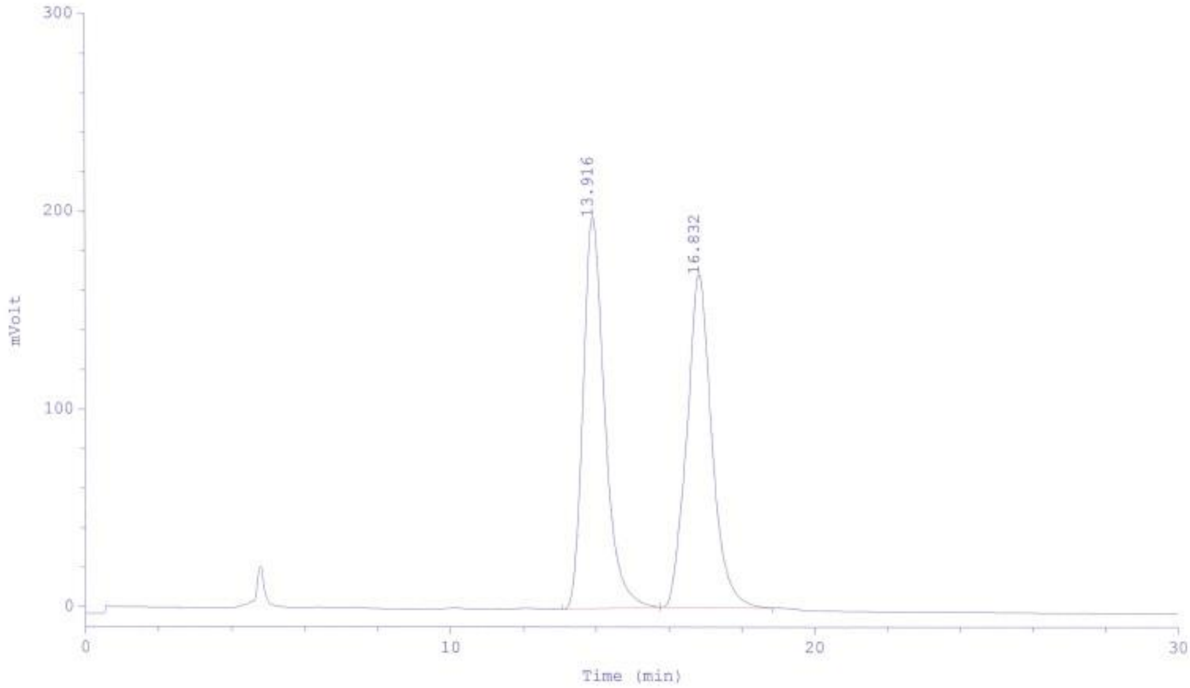

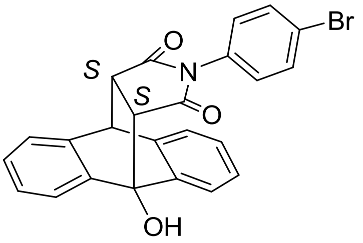

32

Chromatogram of Diels-Alder adduct **32** (Chiralpak IA; *n*-hexane/iPrOH/CH_2Cl_2 64/19/17; 0.7 mL/min)

*Catalyzed by bisoxazoline **33**:*

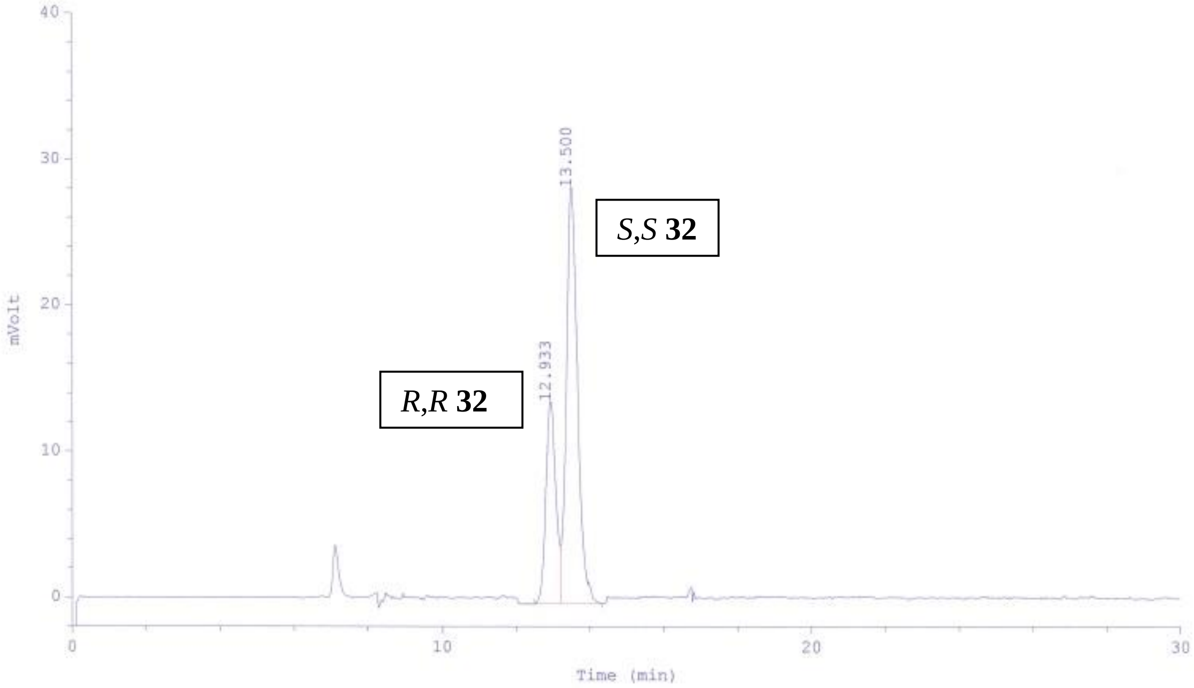

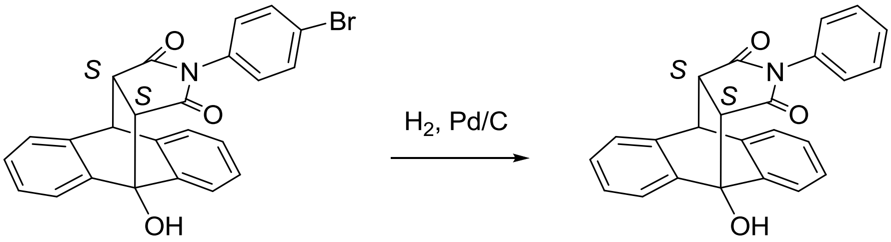

32 **25**

Chromatogram of Diels–Alder adduct **25** (*S*,*S* isomer prevails) obtained by hydrogenation of **32** (Chiralpak IA; *n*-hexane/iPrOH 10/3 + 20% CH_2Cl_2; 0.7 mL/min) → major isomer of **32** (see above) has *S*,*S* configuration

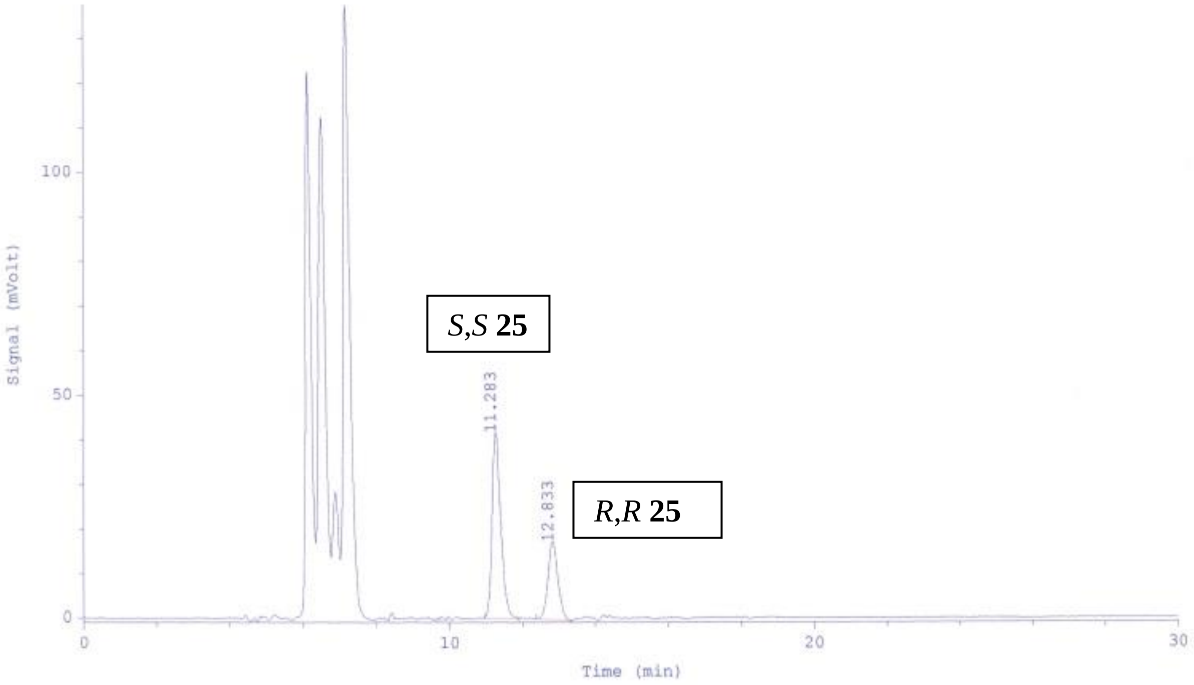

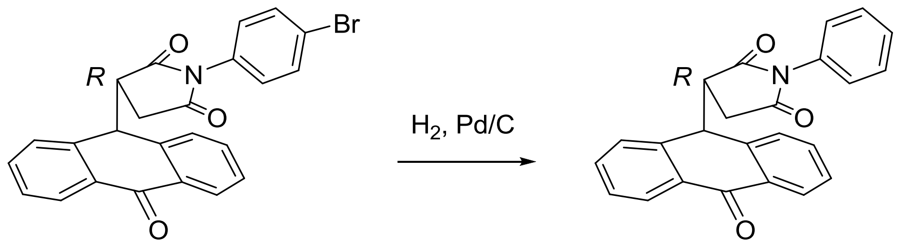

30 28

Chromatogram of retro-aldol product **28** obtained by hydrogenation of *R*-**30** (configuration confirmed by crystal structure determination) (Chiralpak IA; *n*-hexane/iPrOH 10/3 + 20% CH_2Cl_2; 0.7 mL/min)

→ major isomer of **28** has *R* configuration

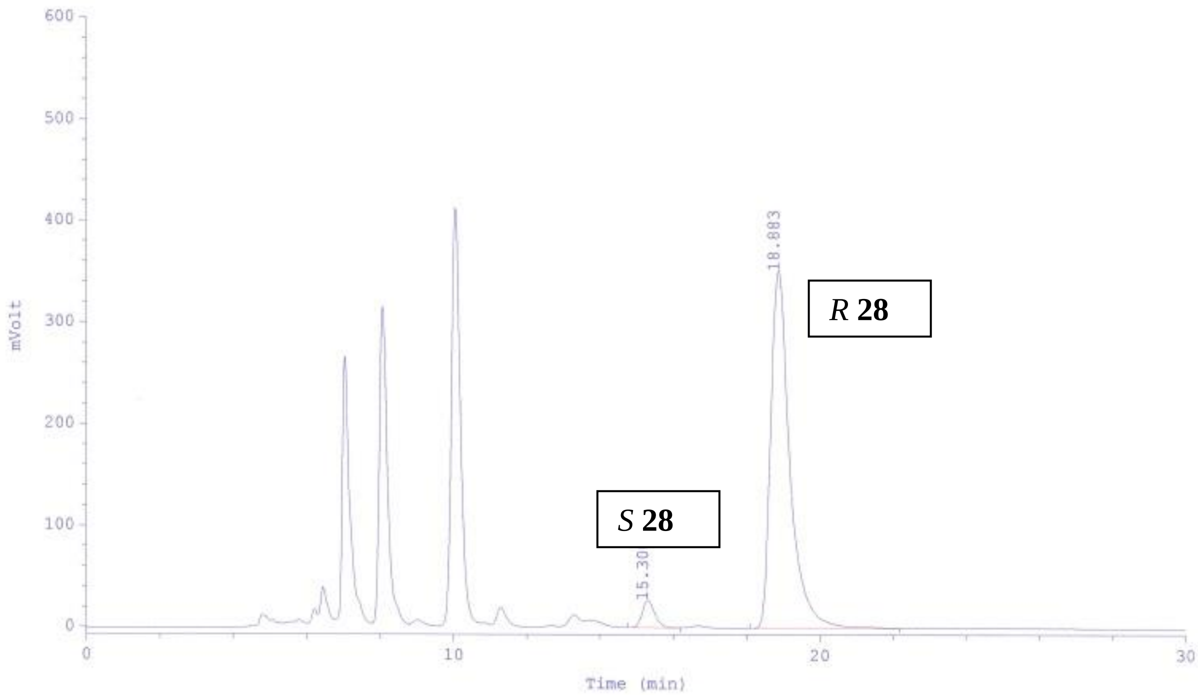

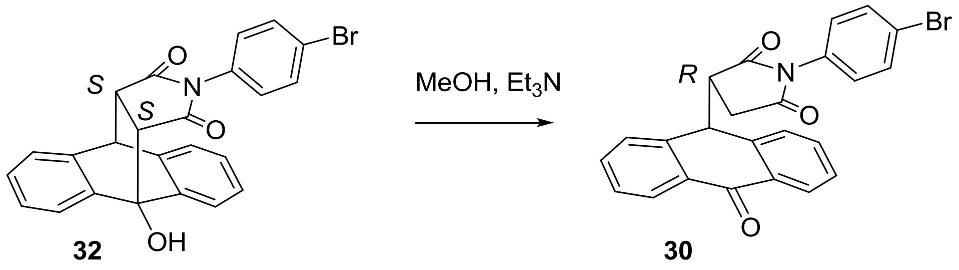

Chromatogram of retro-aldol product **30** (31% *ee*, *R* configuration prevails) obtained by ring opening of **32** (40% *ee*) (Chiralpak IA; *n*-hexane/iPrOH 10/3 + 20% CH_2Cl_2; 0.7 mL/min)

→ major isomer of **32** has *S,S* configuration

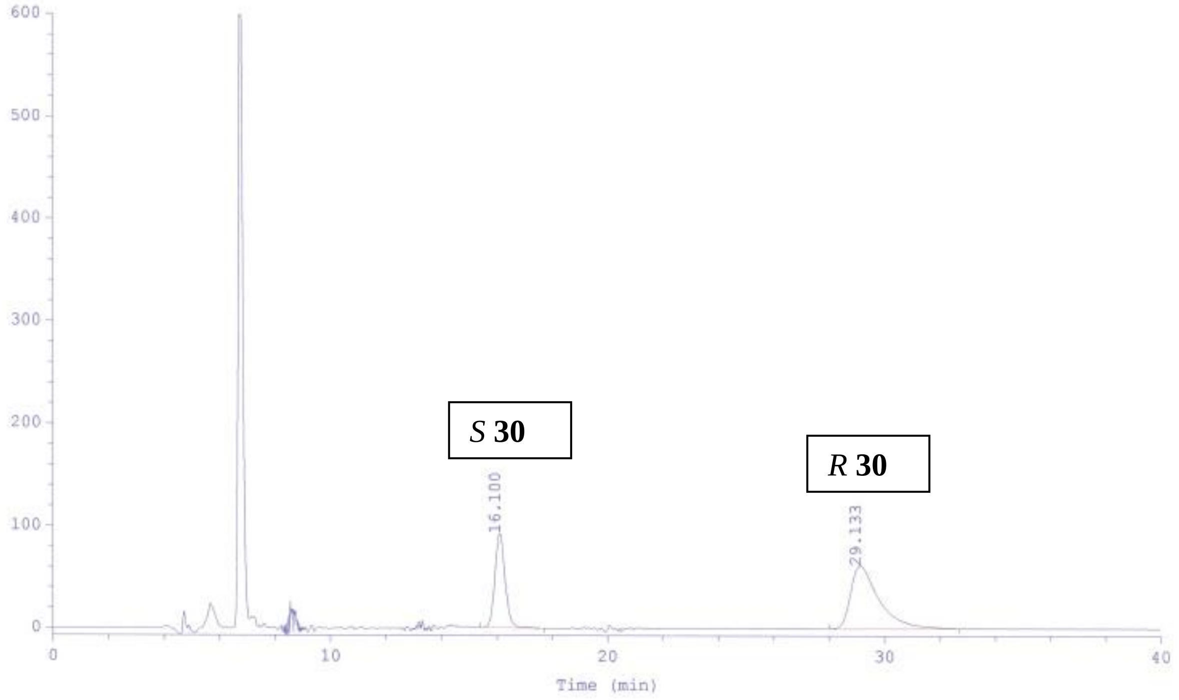

X-ray data of guanidine 10, crystallized as salt with benzoic acid.

A single crystal (colorless block with dimensions 0.26 × 0.36 × 0.40 mm) was measured on a SIEMENS SMART diffractometer at a temperature of about −88 °C. Repeatedly measured reflections remained stable. An empirical absorption correction with program SADABS (Sheldrick, 2000) gave a correction factor between 0.934 and 1.000. Equivalent reflections, including Friedel opposites, were averaged. R(I)internal = 0.127. The structure was determined by direct methods using program SHELXS. The C-bound H atoms were geometrically positioned and were constrained. The N-bound H atoms were taken from a difference synthesis and were refined with a N-H distance constraint of 0.88(2) Å. The structure was refined on F^2 values using program SHELXL-97. The final difference density was between −0.21 and +0.17 e/Å^3.

The asymmetric unit contains two cations of guanidine **10**, two benzoate anions and an ethyl acetate solvate molecule. Each cation is connected by two N-H···O hydrogen bonds to an anion. Each pyrimidine ring approximately has a C5-envelope conformation with the phenyl substituent in a pseudo-axial position. The cation-anion pairs and the ethylacetate solvate groups are connected by a number of very weak intermolecular C-H···π(phenyl) and C-H···O contacts.

Empirical formula	C_{56} H_{62} N_6 O_6	
Formula weight	915.12	
Temperature	185(2) K	
Wavelength	0.71073 Å	
Crystal system, space group	monoclinic, $P2_1$	
Unit cell dimensions	a = 15.2019(12) Å	α = 90 deg.
	b = 9.6219(8) Å	β = 101.0070(10) deg.
	c = 17.1451(14) Å	γ = 90 deg.
Volume	2461.7(3) Å^3	
Z, Calculated density	2, 1.235 Mg/m^3	
Absorption coefficient	0.081 mm^{-1}	
F(000)	976	
Crystal size	0.40 x 0.36 x 0.26 mm^3	
Theta range for data collection	1.64 to 26.00 deg.	
Limiting indices	$-18 \le h \le 18$, $-11 \le k \le 11$, $-21 \le l \le 21$	
Reflections collected / unique	25966 / 5087 [R(int) = 0.1274]	
Completeness to theta = 26.00	99.1 %	
Absorption correction	Semi-empirical from equivalents	

Max. and min. transmission	1.000 and 0.934
Refinement method	Full-matrix least-squares on F^2
Data / restraints / parameters	5087 / 5 / 626
Goodness-of-fit on F^2	1.133
Final R indices [I>2σ(I)]	R1 = 0.0943, wR2 = 0.0972
R indices (all data)	R1 = 0.1706, wR2 = 0.1135
Largest diff. peak and hole	0.172 and -0.211 e/Å^3
Deposition number	CCDC - 1482611

Compound **10** as benzoate salt:

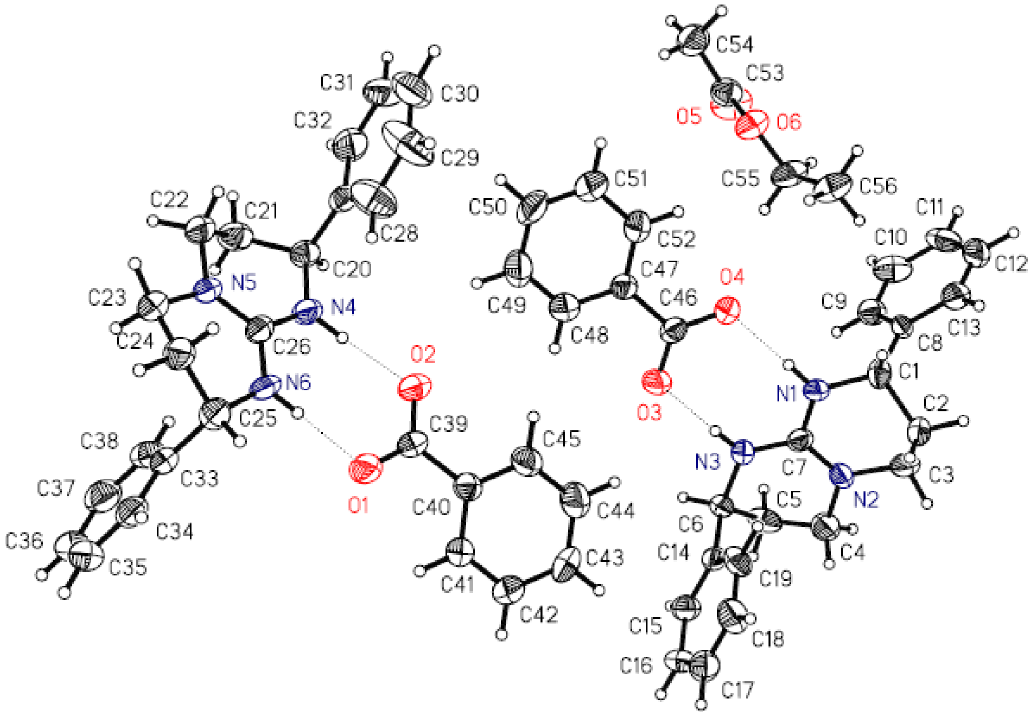

X-ray data of compound 29.

Data for compounds **29** and **30** were collected on a STOE IPDS II two-circle diffractometer with a Genix Microfocus tube with mirror optics using MoK_α radiation (λ = 0.71073 Å) and were scaled using the frame scaling procedure in the *X-AREA* program system (Stoe & Cie, 2002). The structures were solved by direct methods using the program *SHELXS* (Sheldrick, 2008) and refined against F^2 with full-matrix least-squares techniques using the program *SHELXL-97* (Sheldrick, 2008).

Empirical formula	$C_{25}H_{18}ClNO_3$
Formula weight	415.85
Temperature	173(2) K
Wavelength	0.71073 Å

Crystal system	Orthorhombic	
Space group	$P2_12_12_1$	
Unit cell dimensions	a = 6.2818(11) Å	α= 90°.
	b = 18.682(5) Å	β= 90°.
	c = 33.725(5) Å	γ= 90°.
Volume	3957.9(14) $Å^3$	
Z	8	
Density (calculated)	1.396 Mg/m^3	
Absorption coefficient	0.221 mm^{-1}	
F(000)	1728	
Crystal size	0.290 x 0.020 x 0.020 mm^3	
Theta range for data collection	2.262 to 26.261°.	
Index ranges	$-7 \leq h \leq 7$, $-22 \leq k \leq 23$, $-41 \leq l \leq 39$	
Reflections collected	34726	
Independent reflections	7819 [R(int) = 0.4418]	
Completeness to theta = 25.000°	99.9 %	
Absorption correction	Semi-empirical from equivalents	
Max. and min. transmission	1.000 and 0.147	
Refinement method	Full-matrix least-squares on F^2	
Data / restraints / parameters	7819 / 510 / 543	
Goodness-of-fit on F2	0.978	
Final R indices [I>2σ(I)]	R1 = 0.1530, wR2 = 0.2038	
R indices (all data)	R1 = 0.2976, wR2 = 0.2666	
Absolute structure parameter	0.4(4)	
Largest diff. peak and hole	0.341 and -0.382 e/$Å^3$	
Deposition number	CCDC - 1482612	

compound **29**:

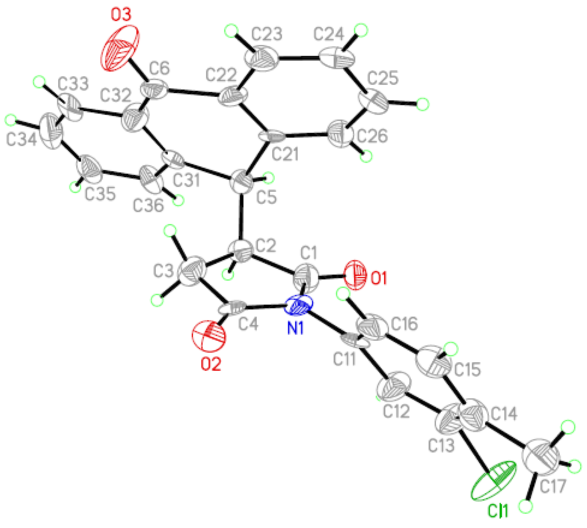

X-ray data of compound 30.

Empirical formula	$C_{24}H_{16}BrNO_3$	
Formula weight	446.29	
Temperature	173(2) K	
Wavelength	0.71073 Å	
Crystal system	Orthorhombic	
Space group	$P2_12_12_1$	
Unit cell dimensions	a = 5.7034(5) Å	α= 90°.
	b = 11.3752(11) Å	β= 90°.
	c = 29.276(2) Å	γ = 90°.
Volume	1899.3(3) $Å^3$	
Z	4	
Density (calculated)	1.561 Mg/m^3	
Absorption coefficient	2.191 mm^{-1}	
F(000)	904	
Crystal size	0.260 x 0.040 x 0.010 mm^3	
Theta range for data collection	1.921 to 24.998°.	
Index ranges	$-5 \leq h \leq 6$, $-13 \leq k \leq 13$, $-34 \leq l \leq 32$	
Reflections collected	9377	
Independent reflections	3341 [R(int) = 0.1359]	
Completeness to theta = 25.000°	99.8 %	
Absorption correction	Semi-empirical from equivalents	
Max. and min. transmission	1.000 and 0.382	
Refinement method	Full-matrix least-squares on F^2	
Data / restraints / parameters	3341 / 0 / 262	
Goodness-of-fit on F2	0.811	
Final R indices [I>2σ(I)]	R1 = 0.0547, wR2 = 0.0886	
R indices (all data)	R1 = 0.1305, wR2 = 0.1099	
Absolute structure parameter	-0.01(3)	
Largest diff. peak and hole	0.276 and -0.289 e/$Å^3$	
Deposition number	CCDC - 1482613	

The absolute configuration of compound **30** is *R*.

Compound **30**:

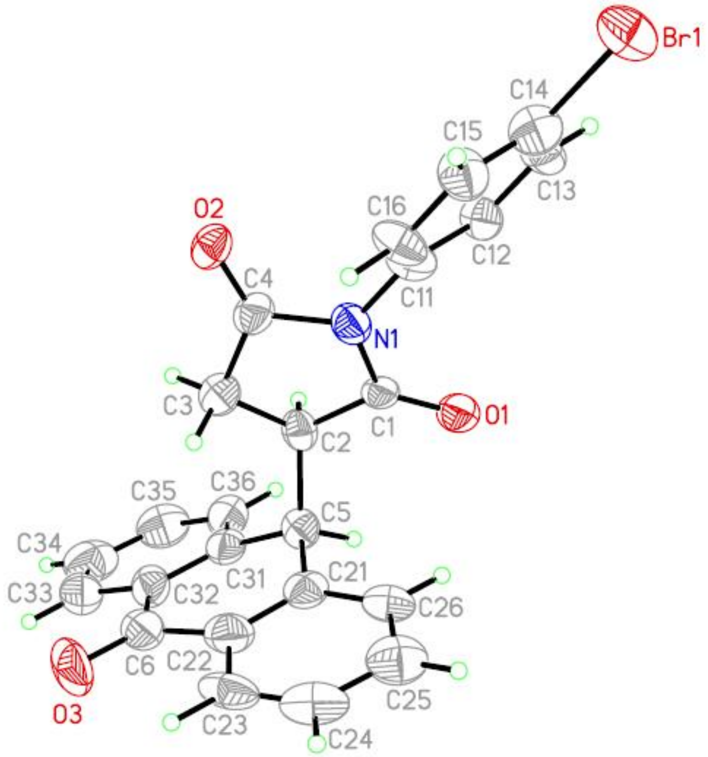
Br1
C14
C15
C13
C16
C12
O2
C11
C4
N1
C3
C1
O1
C2
C35
C36
C5
C34
C31
C21
C33
C32
C26
C6
C22
O3
C25
C23
C24

Copies of ^{1}H and ^{13}C NMR spectra of new compounds

Compound *rac*-**13**:

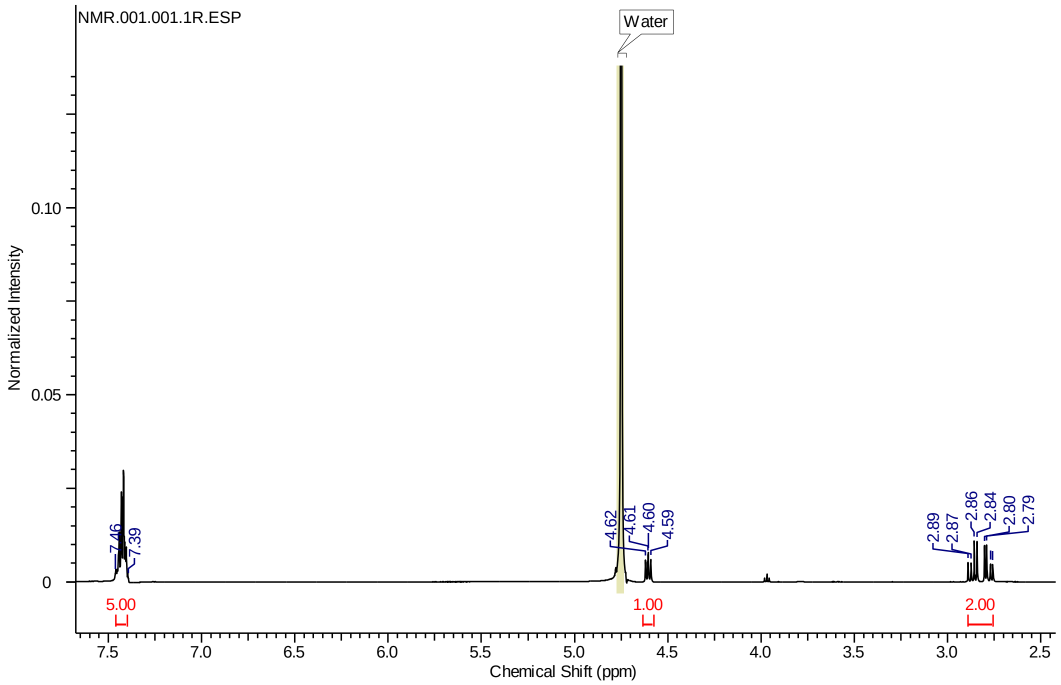

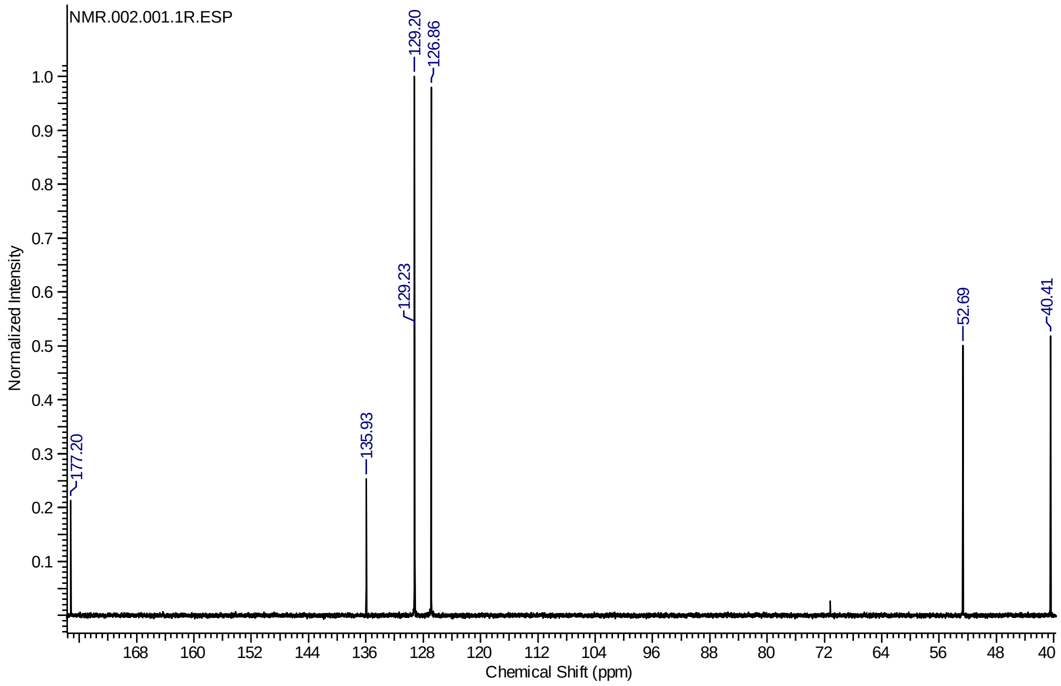

Compound *rac*-**12**:

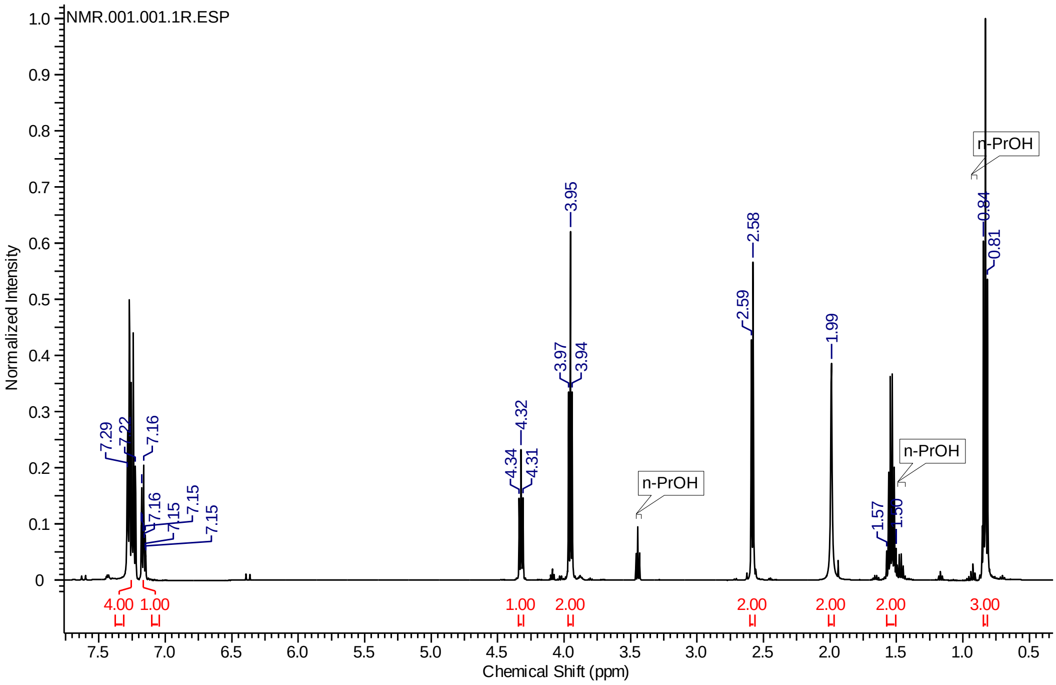

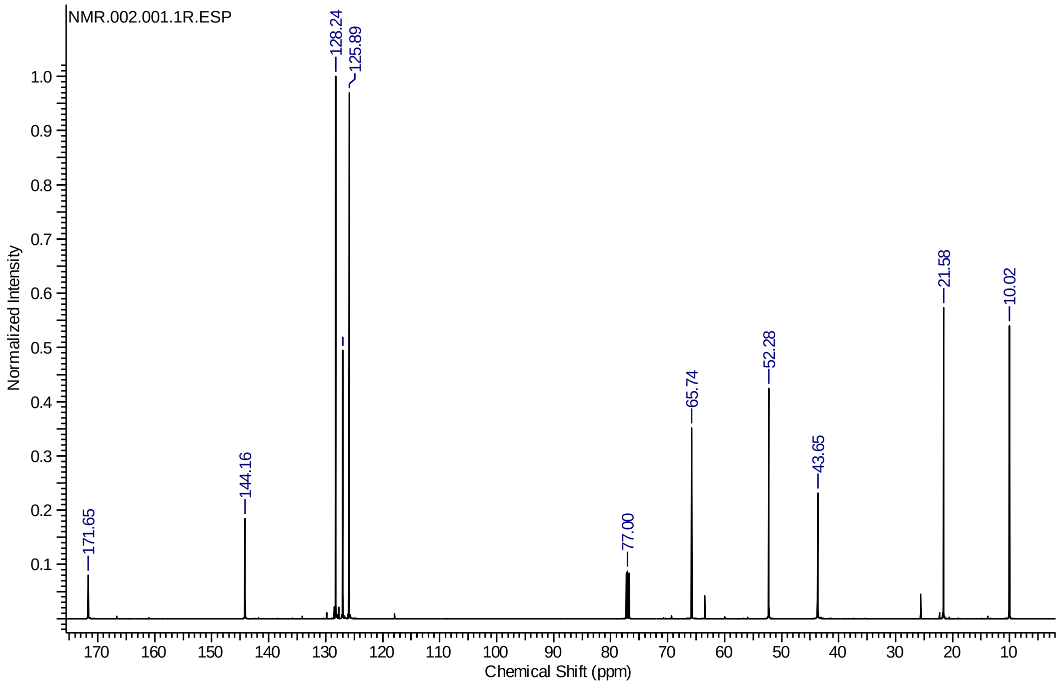

Compound **13**:

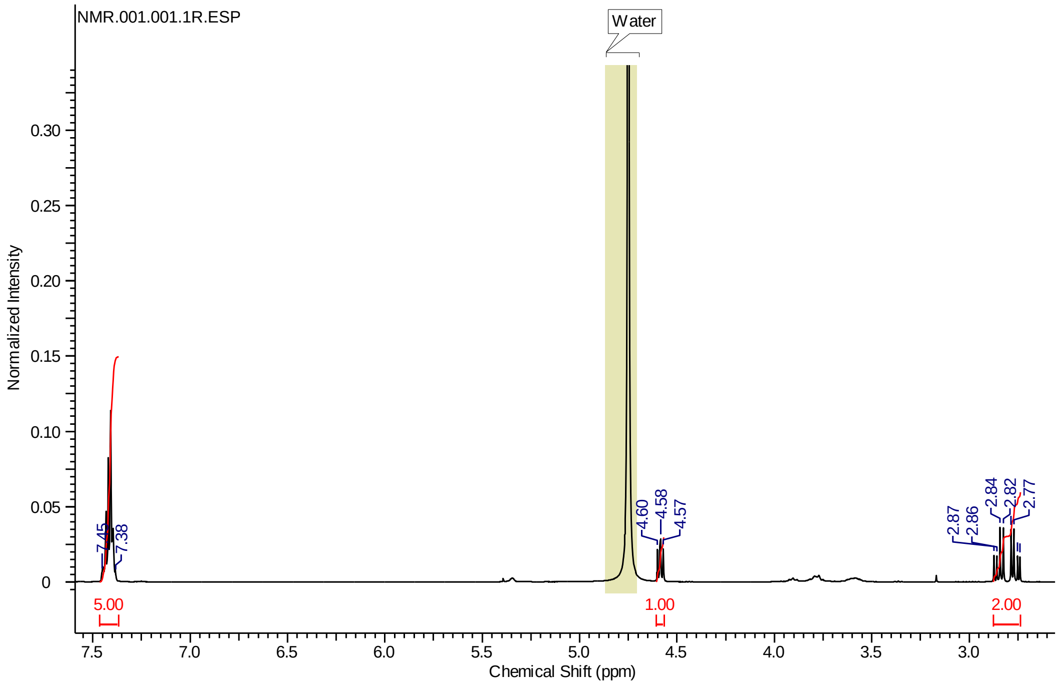

NMR.001.001.1R.ESP
Water
Normalized Intensity
Chemical Shift (ppm)
7.45
7.38
4.60
4.58
4.57
2.87
2.86
2.84
2.82
2.77
5.00
1.00
2.00

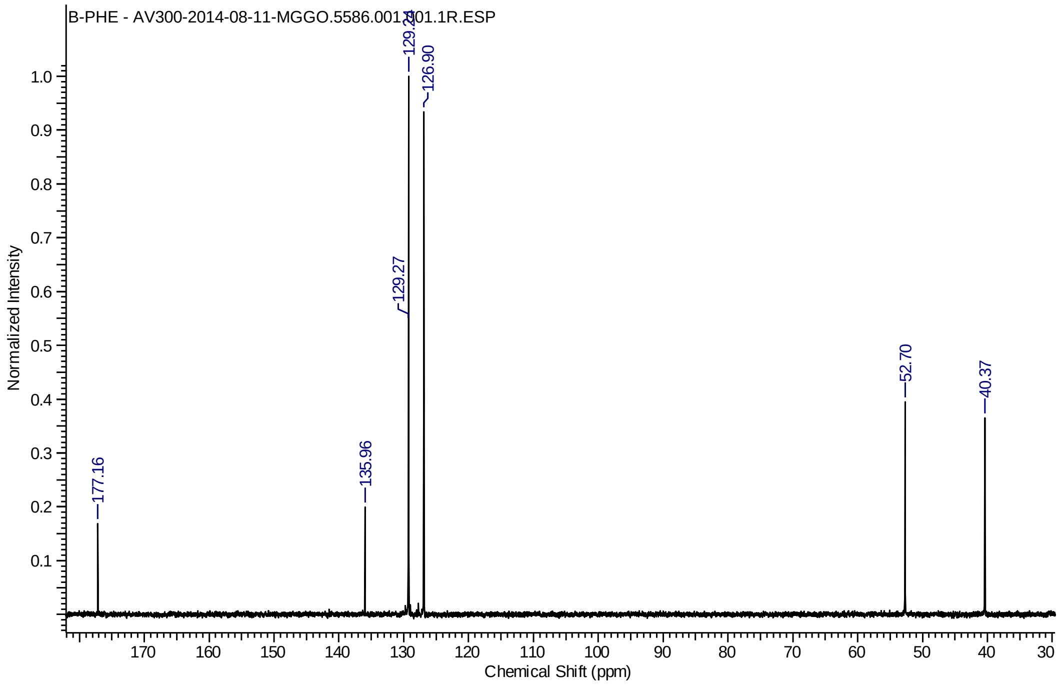

B-PHE - AV300-2014-08-11-MGGO.5586.001.001.1R.ESP
Normalized Intensity
Chemical Shift (ppm)
177.16
135.96
129.27
126.90
52.70
40.37

Compound **34**:

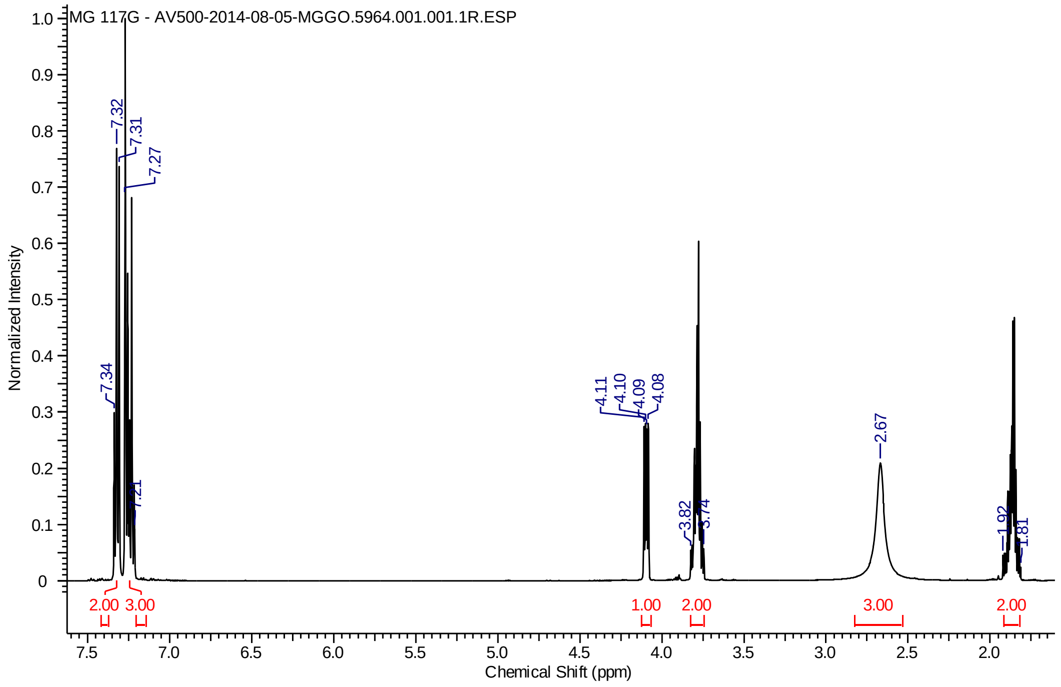

MG 117G - AV500-2014-08-05-MGGO.5964.001.001.1R.ESP
Normalized Intensity
Chemical Shift (ppm)
7.34
7.32
7.31
7.27
7.21
4.11
4.10
4.09
4.08
3.82
3.74
2.67
1.92
1.81
2.00
3.00
1.00
2.00
3.00
2.00

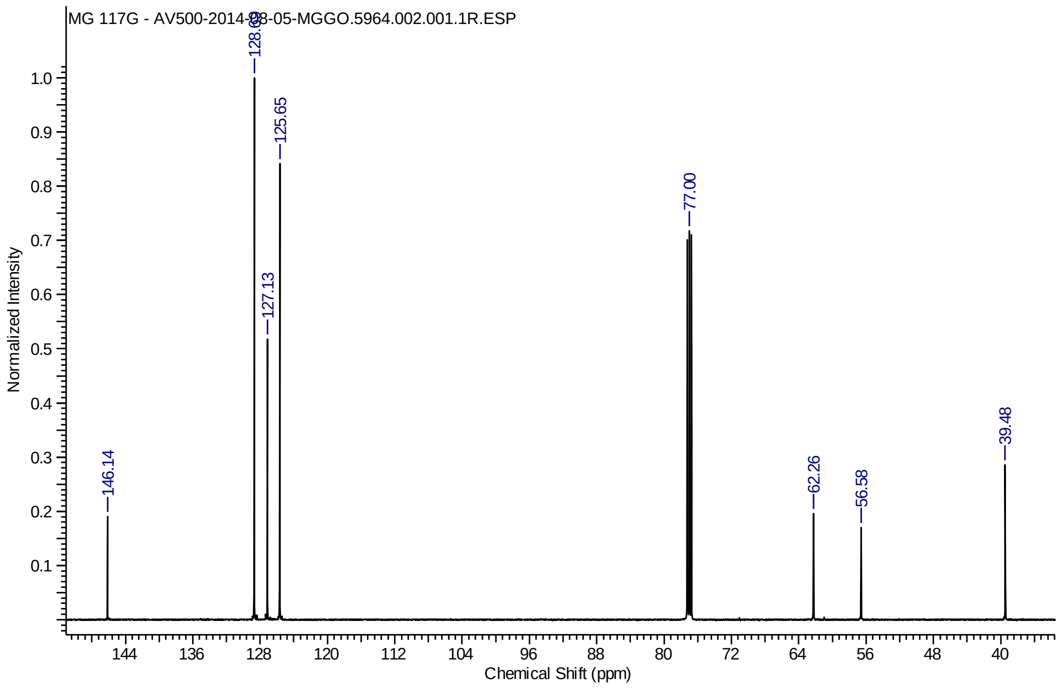

MG 117G - AV500-2014-08-05-MGGO.5964.002.001.1R.ESP
Normalized Intensity
Chemical Shift (ppm)
146.14
127.13
125.65
77.00
62.26
56.58
39.48

Compound **14**:

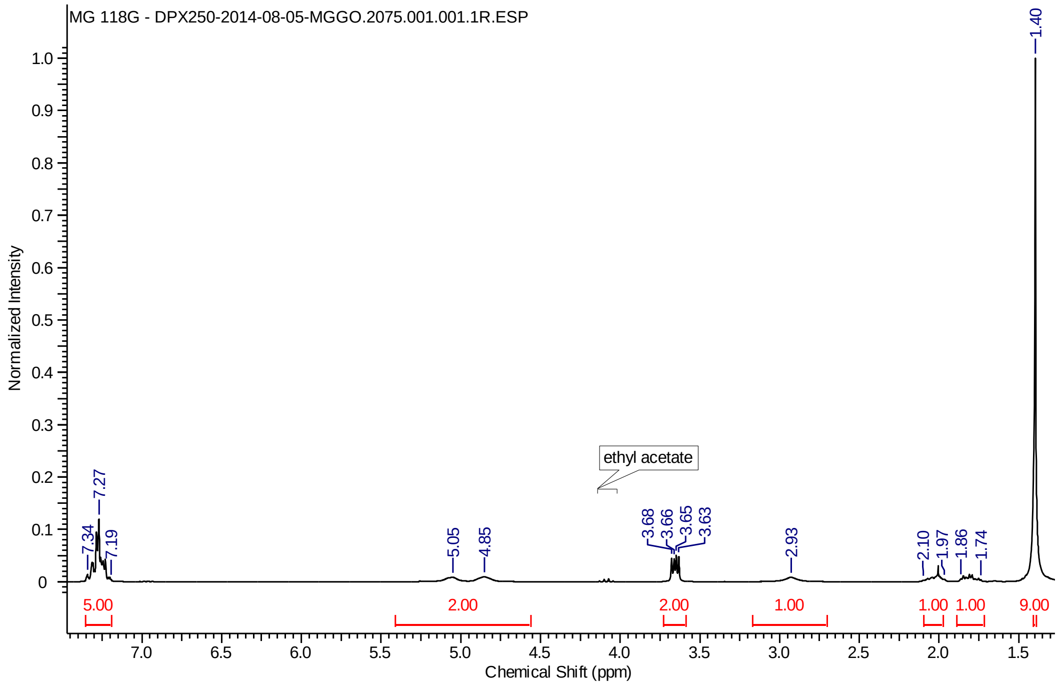
MG 118G - DPX250-2014-08-05-MGGO.2075.001.001.1R.ESP
Normalized Intensity
Chemical Shift (ppm)
ethyl acetate
7.34
7.27
7.19
5.05
4.85
3.68
3.66
3.65
3.63
2.93
2.10
1.97
1.86
1.74
1.40
5.00
2.00
2.00
1.00
1.00
1.00
9.00

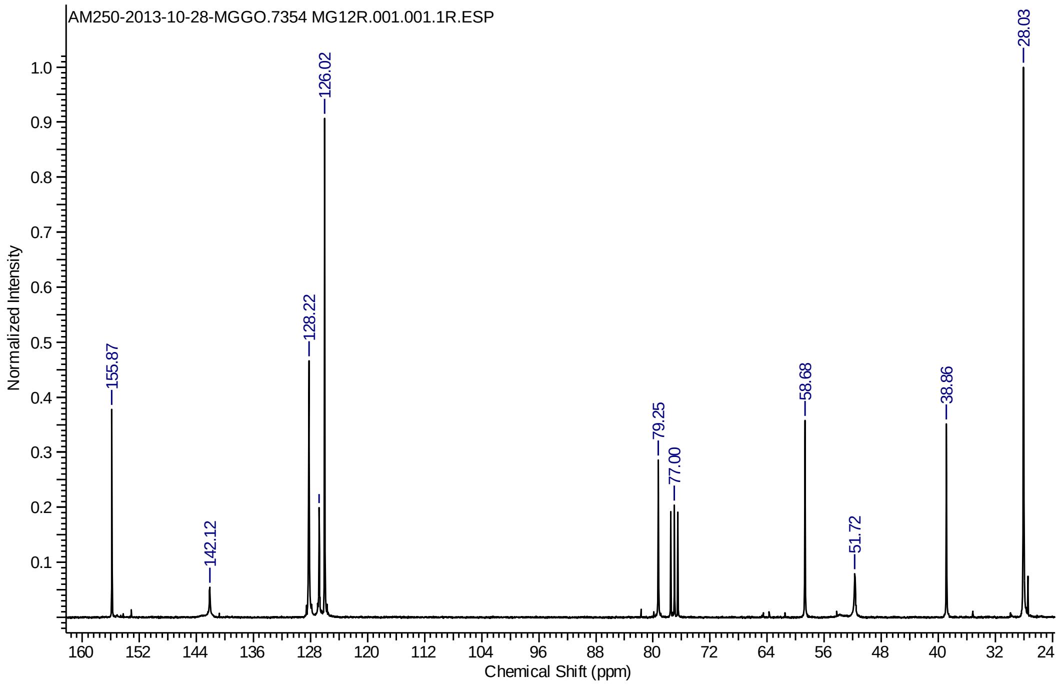
AM250-2013-10-28-MGGO.7354 MG12R.001.001.1R.ESP
Normalized Intensity
Chemical Shift (ppm)
155.87
142.12
128.22
126.02
79.25
77.00
58.68
51.72
38.86
28.03

Compound **35**:

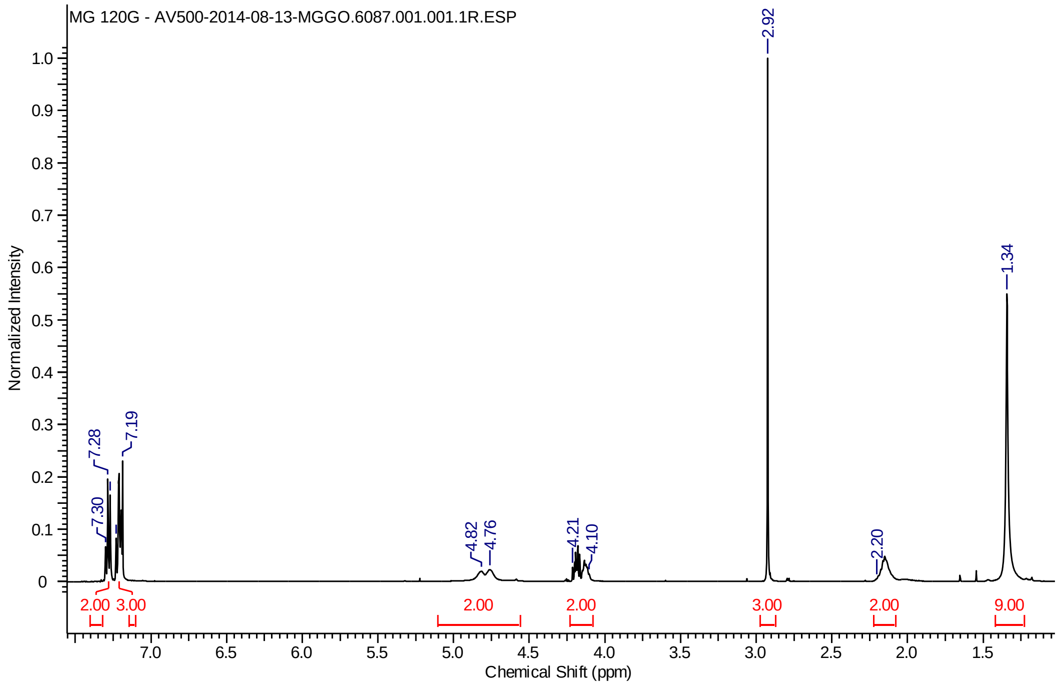
MG 120G - AV500-2014-08-13-MGGO.6087.001.001.1R.ESP
Normalized Intensity
Chemical Shift (ppm)
2.92
1.34
7.28
7.19
7.30
4.82
4.76
4.21
4.10
2.20
2.00
3.00
2.00
2.00
3.00
2.00
9.00

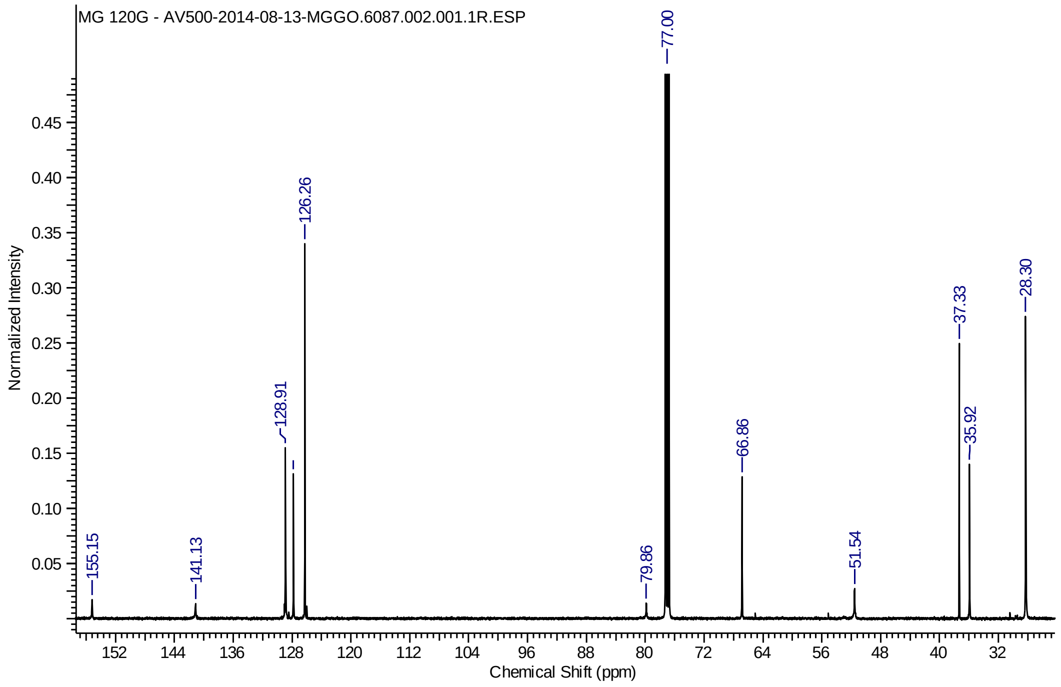
MG 120G - AV500-2014-08-13-MGGO.6087.002.001.1R.ESP
Normalized Intensity
Chemical Shift (ppm)
77.00
126.26
128.91
155.15
141.13
79.86
66.86
51.54
37.33
35.92
28.30

Compound **15**:

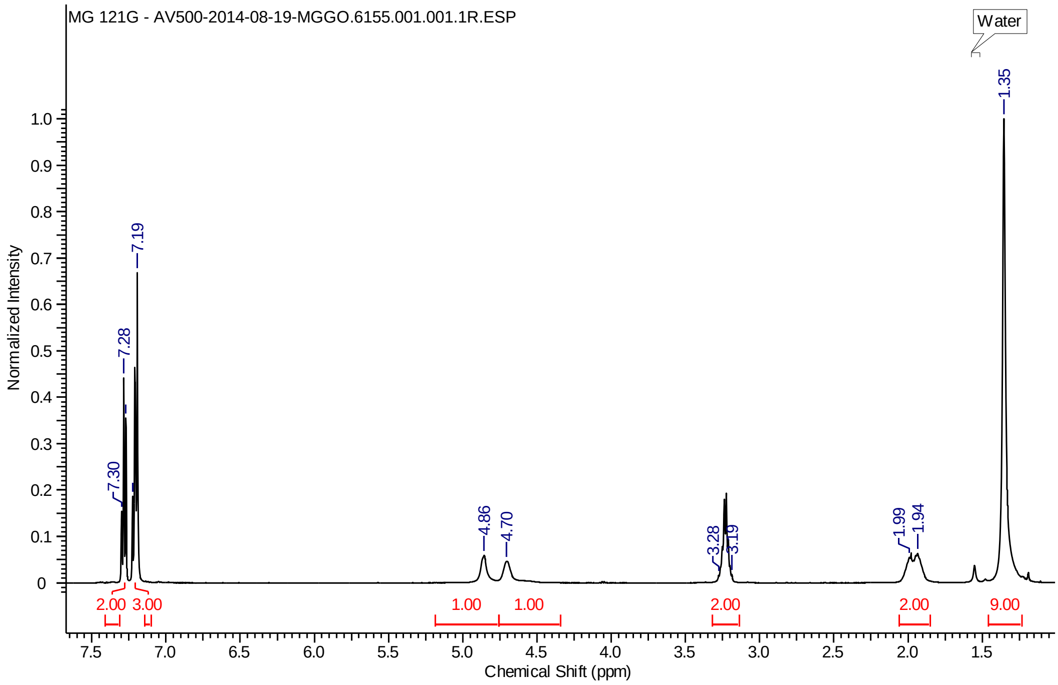
MG 121G - AV500-2014-08-19-MGGO.6155.001.001.1R.ESP
Water
Normalized Intensity
Chemical Shift (ppm)
7.30
7.28
7.19
4.86
4.70
3.28
3.19
1.99
1.94
1.35
2.00
3.00
1.00
1.00
2.00
2.00
9.00

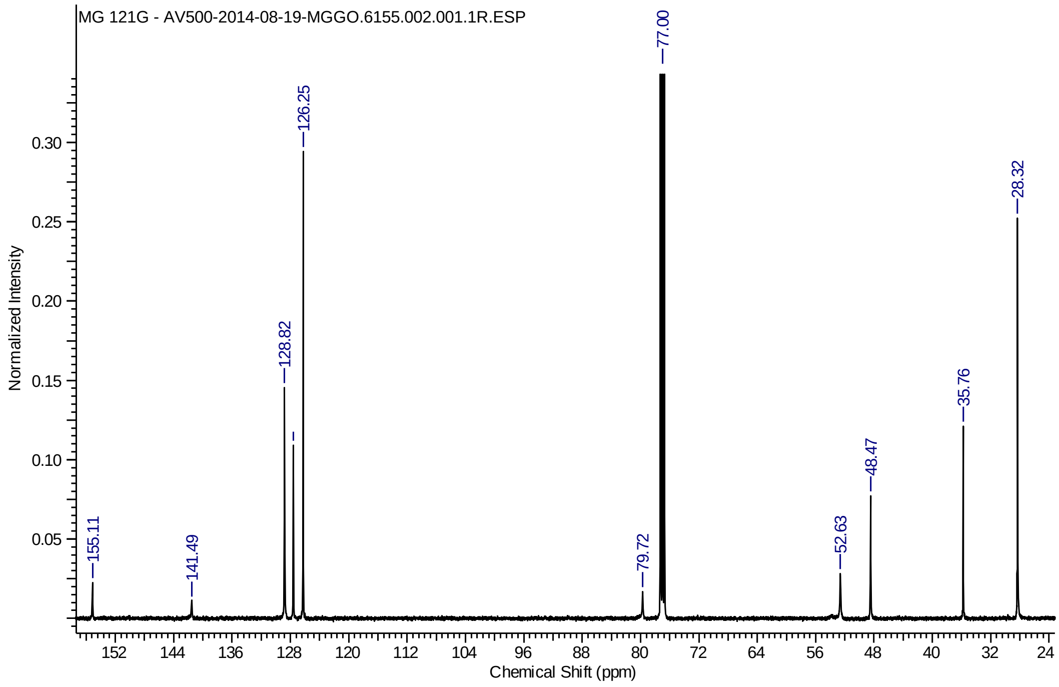
MG 121G - AV500-2014-08-19-MGGO.6155.002.001.1R.ESP
Normalized Intensity
Chemical Shift (ppm)
155.11
141.49
128.82
126.25
79.72
77.00
52.63
48.47
35.76
28.32

Compound **16**:

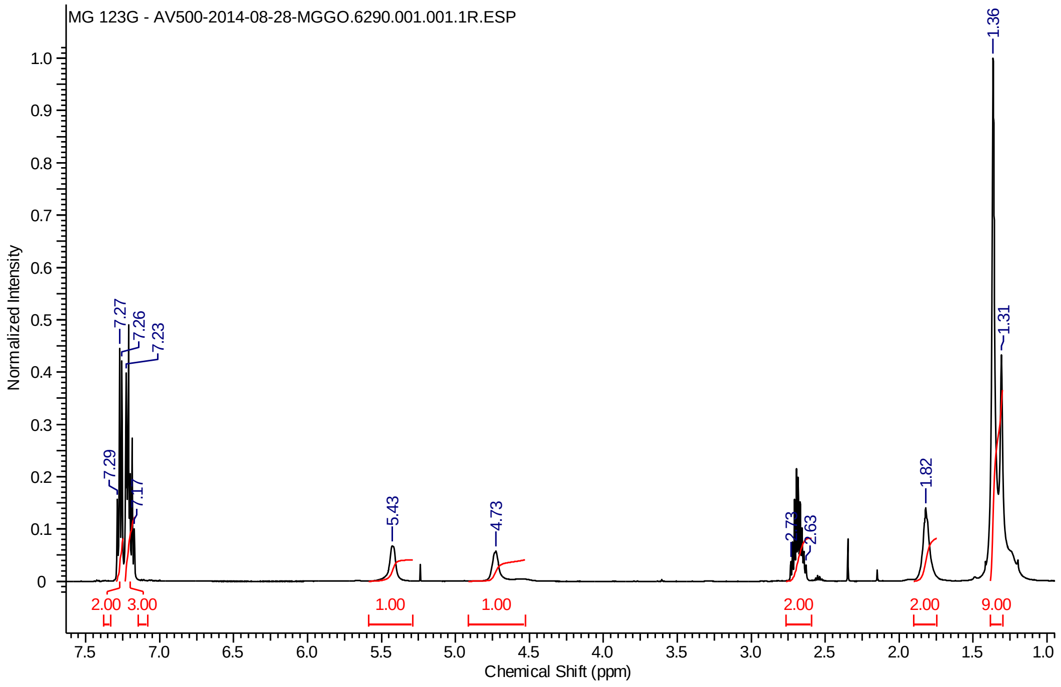
MG 123G - AV500-2014-08-28-MGGO.6290.001.001.1R.ESP
Normalized Intensity
Chemical Shift (ppm)
7.29
7.27
7.26
7.23
7.17
5.43
4.73
2.73
2.63
1.82
1.36
1.31
2.00
3.00
1.00
1.00
2.00
2.00
9.00

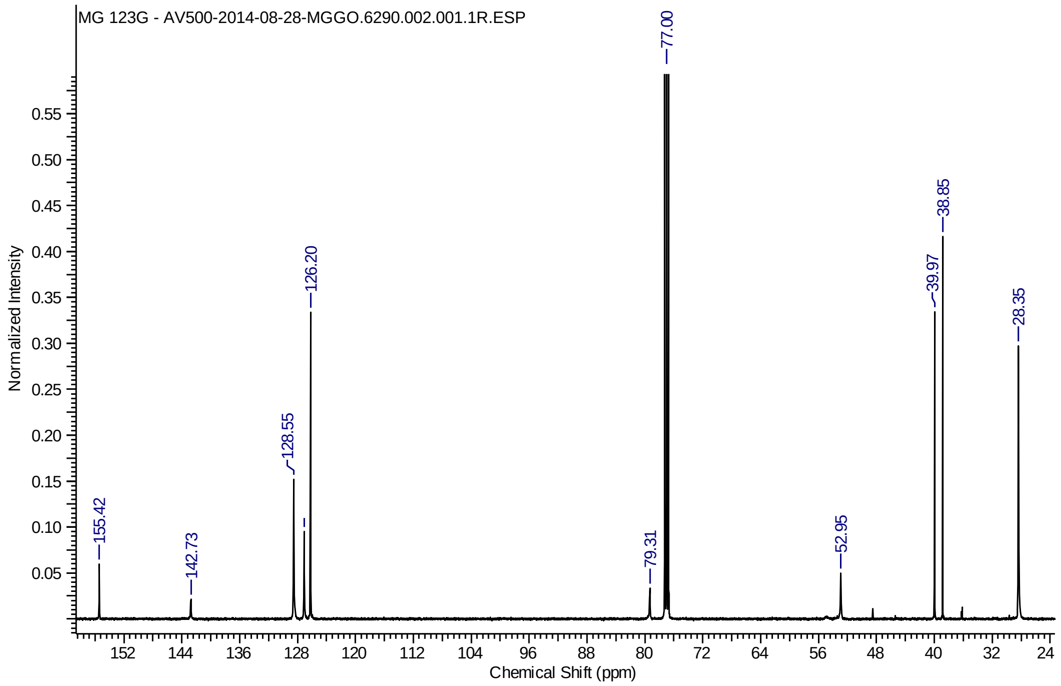
MG 123G - AV500-2014-08-28-MGGO.6290.002.001.1R.ESP
Normalized Intensity
Chemical Shift (ppm)
155.42
142.73
128.55
126.20
79.31
77.00
52.95
39.97
38.85
28.35

Compound **17**:

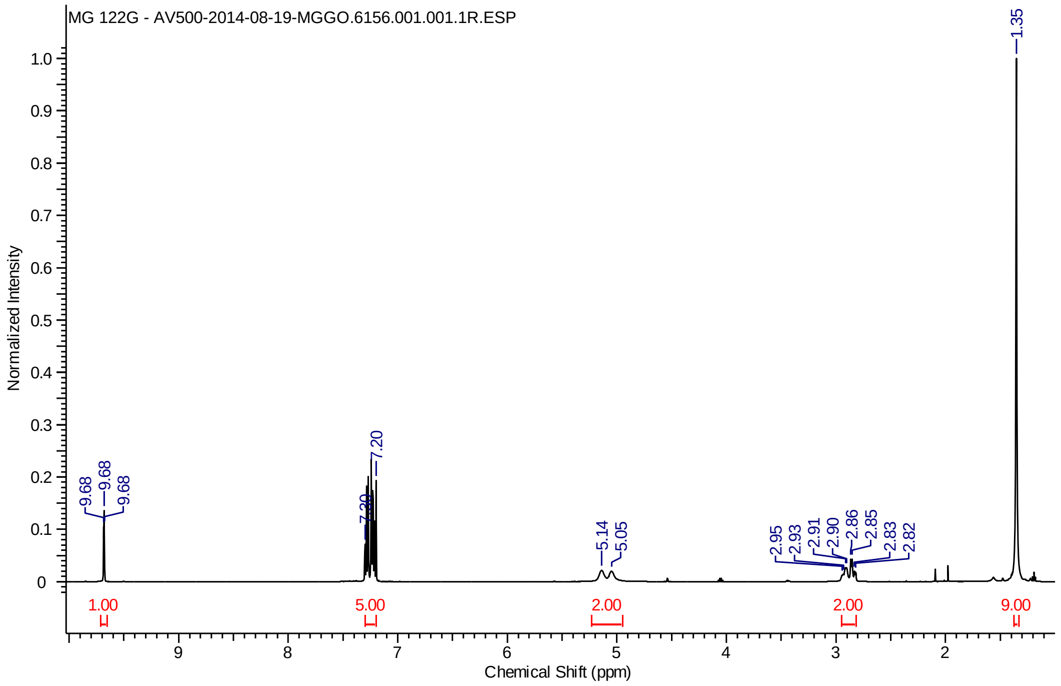

MG 122G - AV500-2014-08-19-MGGO.6156.001.001.1R.ESP
Normalized Intensity
Chemical Shift (ppm)
9.68
9.68
9.68
7.20
7.20
5.14
5.05
2.95
2.93
2.91
2.90
2.86
2.85
2.83
2.82
1.35
1.00
5.00
2.00
2.00
9.00

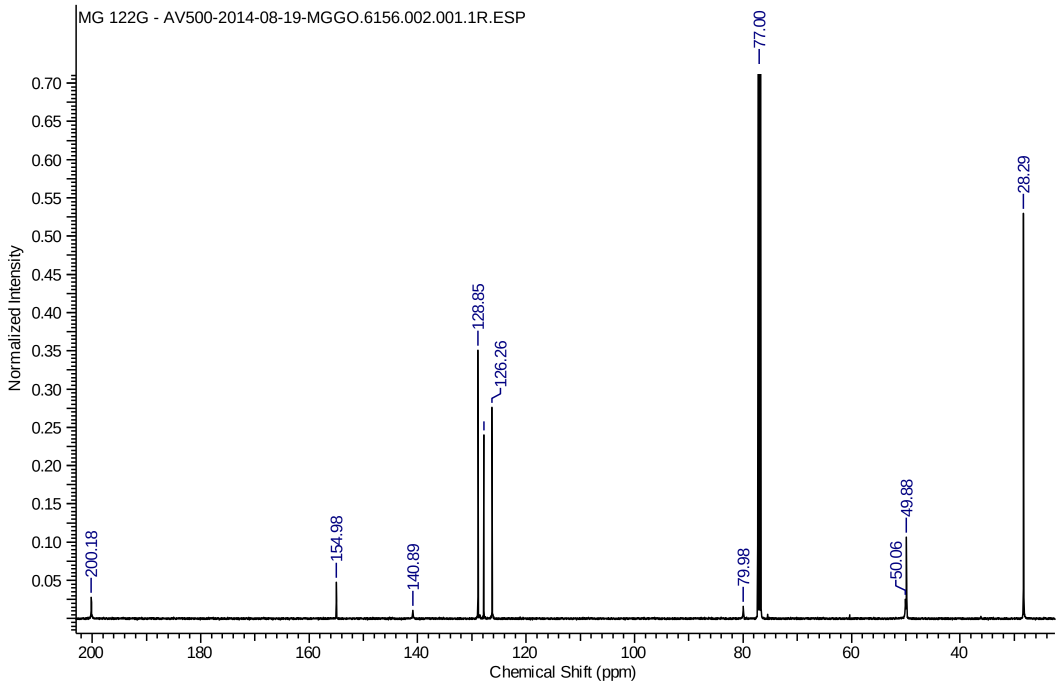

MG 122G - AV500-2014-08-19-MGGO.6156.002.001.1R.ESP
Normalized Intensity
Chemical Shift (ppm)
200.18
154.98
140.89
128.85
126.26
79.98
77.00
50.06
49.88
28.29

Compound **18**:

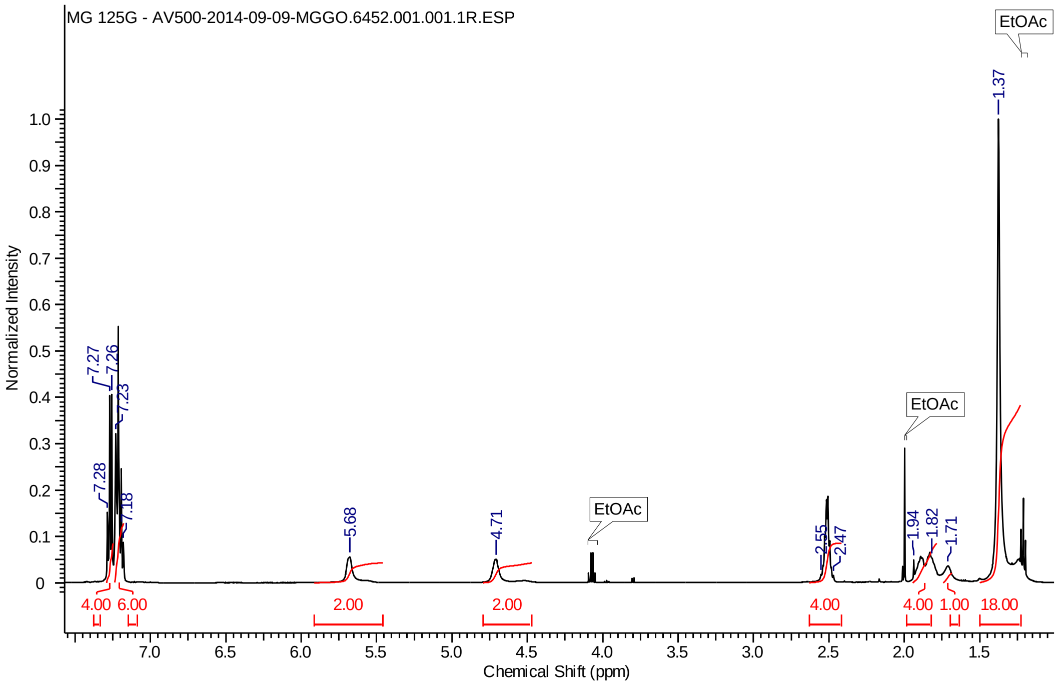
MG 125G - AV500-2014-09-09-MGGO.6452.001.001.1R.ESP
EtOAc
EtOAc
EtOAc
7.27
7.26
7.23
7.28
7.18
5.68
4.71
2.55
2.47
1.94
1.82
1.71
1.37
4.00
6.00
2.00
2.00
4.00
4.00
1.00
18.00
Normalized Intensity
Chemical Shift (ppm)

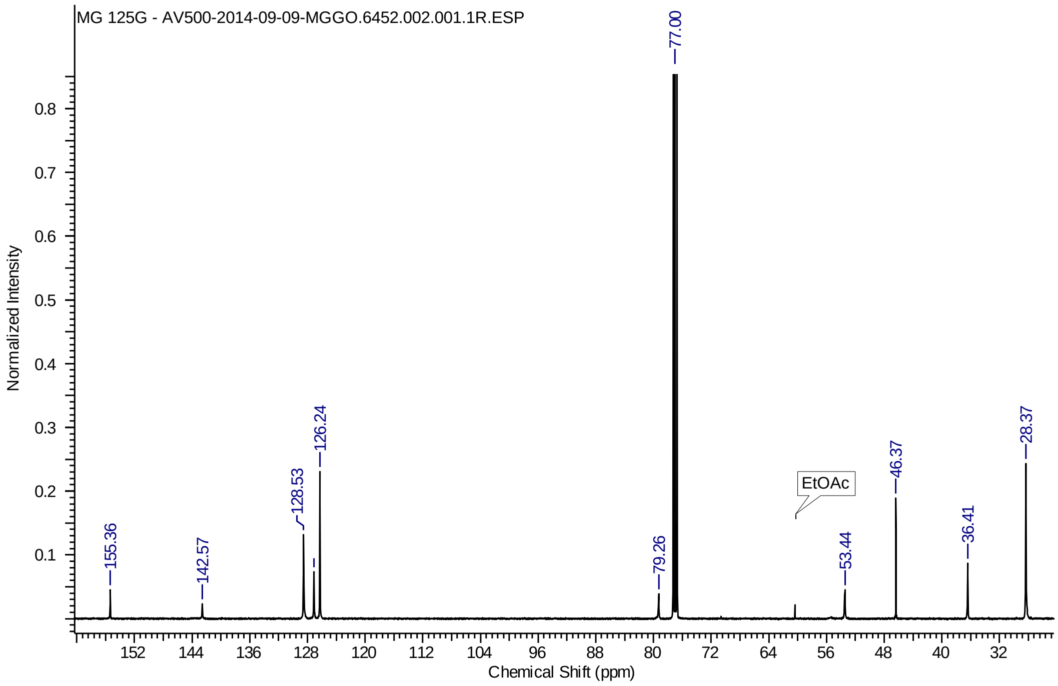
MG 125G - AV500-2014-09-09-MGGO.6452.002.001.1R.ESP
77.00
EtOAc
155.36
142.57
128.53
126.24
79.26
53.44
46.37
36.41
28.37
Normalized Intensity
Chemical Shift (ppm)

Compound **19**:

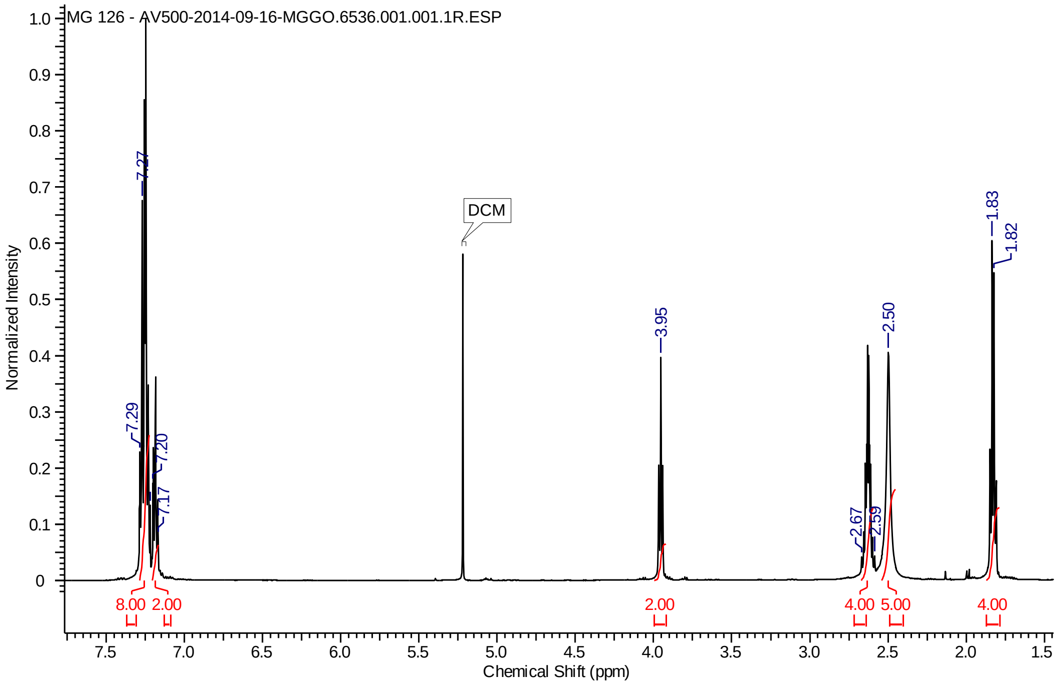
MG 126 - AV500-2014-09-16-MGGO.6536.001.001.1R.ESP
DCM
Normalized Intensity
Chemical Shift (ppm)
7.29
7.27
7.20
7.17
3.95
2.67
2.59
2.50
1.83
1.82
8.00
2.00
2.00
4.00
5.00
4.00

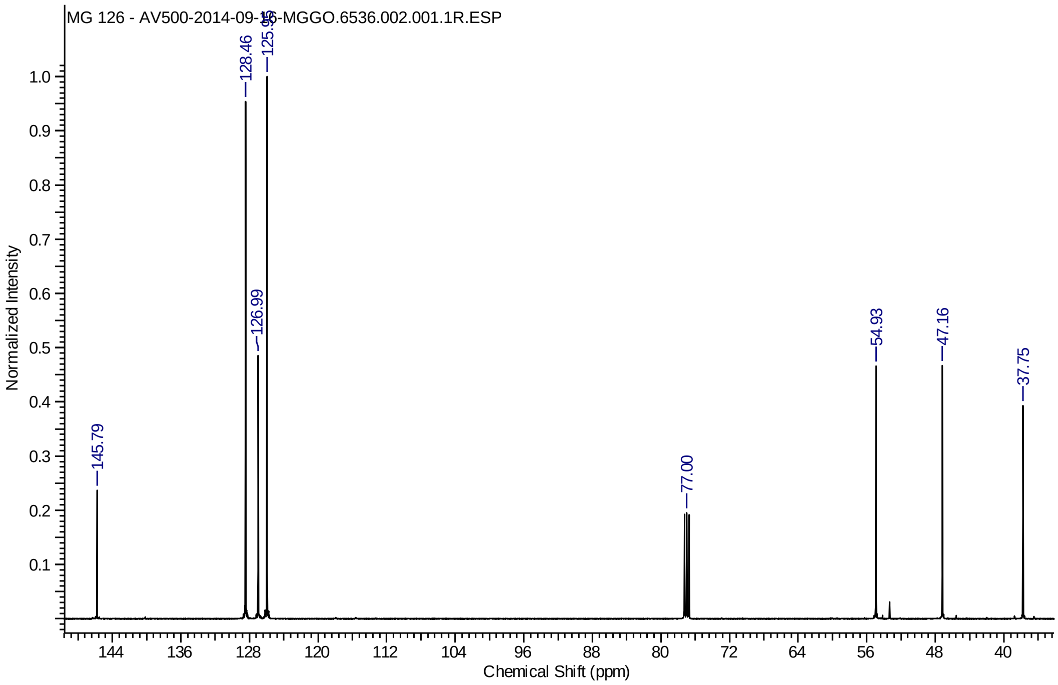
MG 126 - AV500-2014-09-16-MGGO.6536.002.001.1R.ESP
Normalized Intensity
Chemical Shift (ppm)
145.79
128.46
126.99
54.93
47.16
37.75
77.00

Compound **10a**:

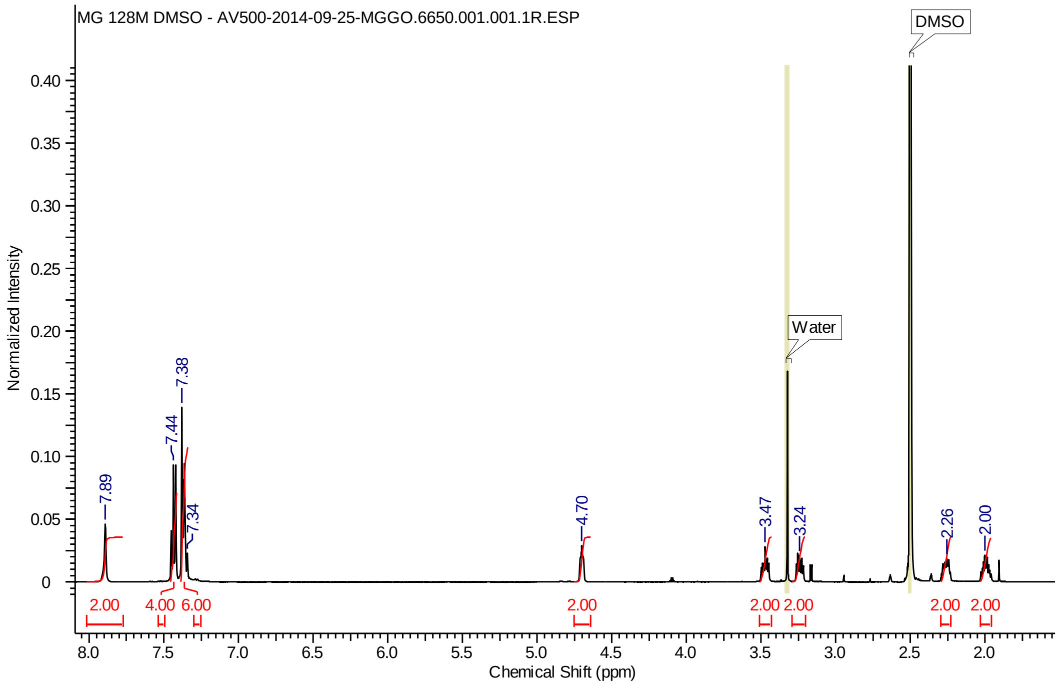

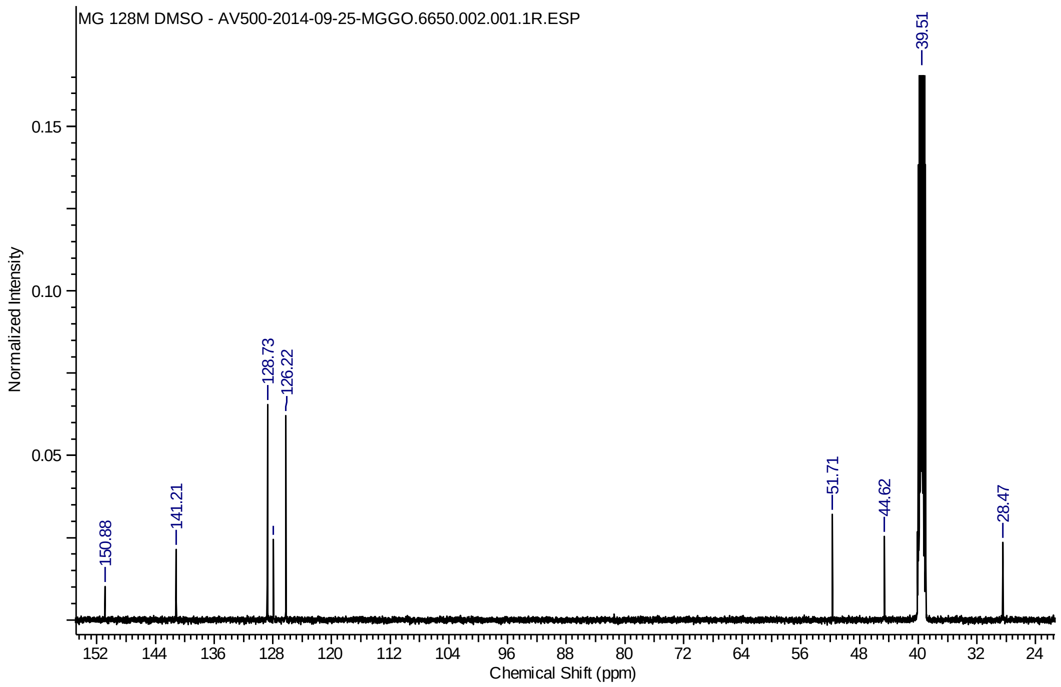

Compound **10**:

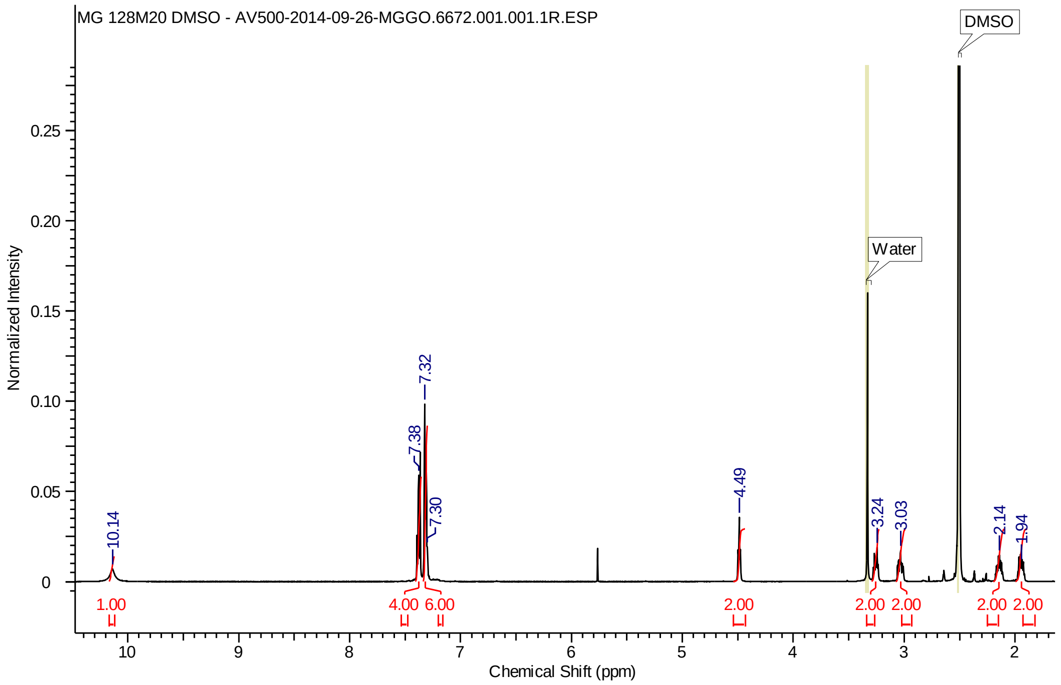

MG 128M20 DMSO - AV500-2014-09-26-MGGO.6672.001.001.1R.ESP
DMSO
Water
Normalized Intensity
0.25
0.20
0.15
0.10
0.05
0
10.14
7.38
7.32
7.30
4.49
3.24
3.03
2.14
1.94
1.00
4.00
6.00
2.00
2.00
2.00
2.00
2.00
10
9
8
7
6
5
4
3
2
Chemical Shift (ppm)

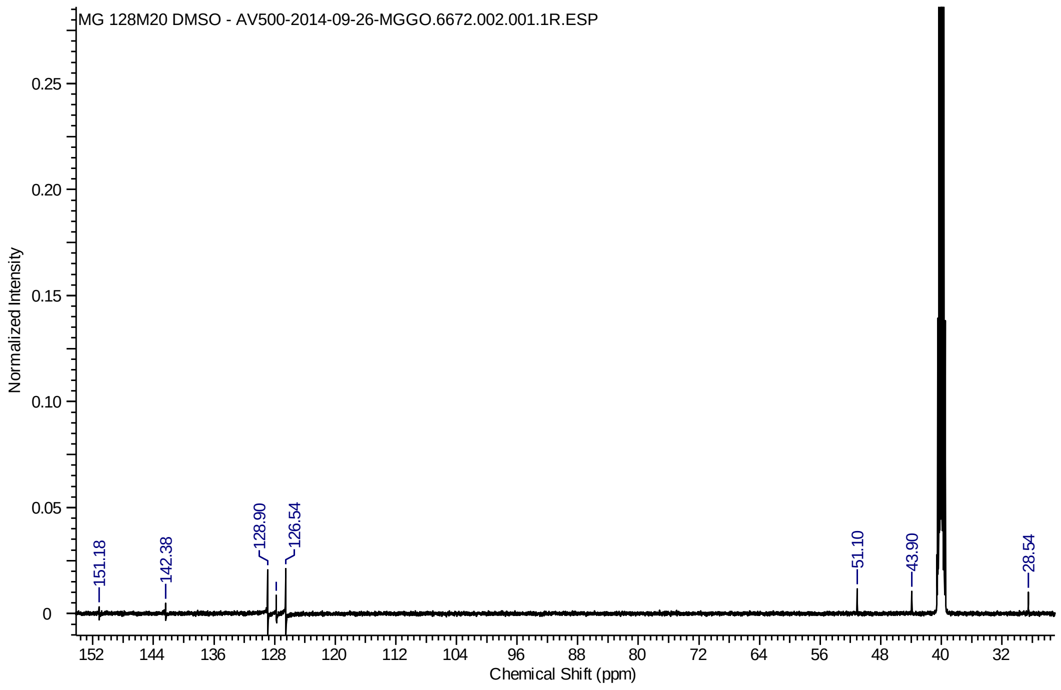

MG 128M20 DMSO - AV500-2014-09-26-MGGO.6672.002.001.1R.ESP
Normalized Intensity
0.25
0.20
0.15
0.10
0.05
0
151.18
142.38
128.90
126.54
51.10
43.90
28.54
152
144
136
128
120
112
104
96
88
80
72
64
56
48
40
32
Chemical Shift (ppm)

Compound *rac*-**25**:

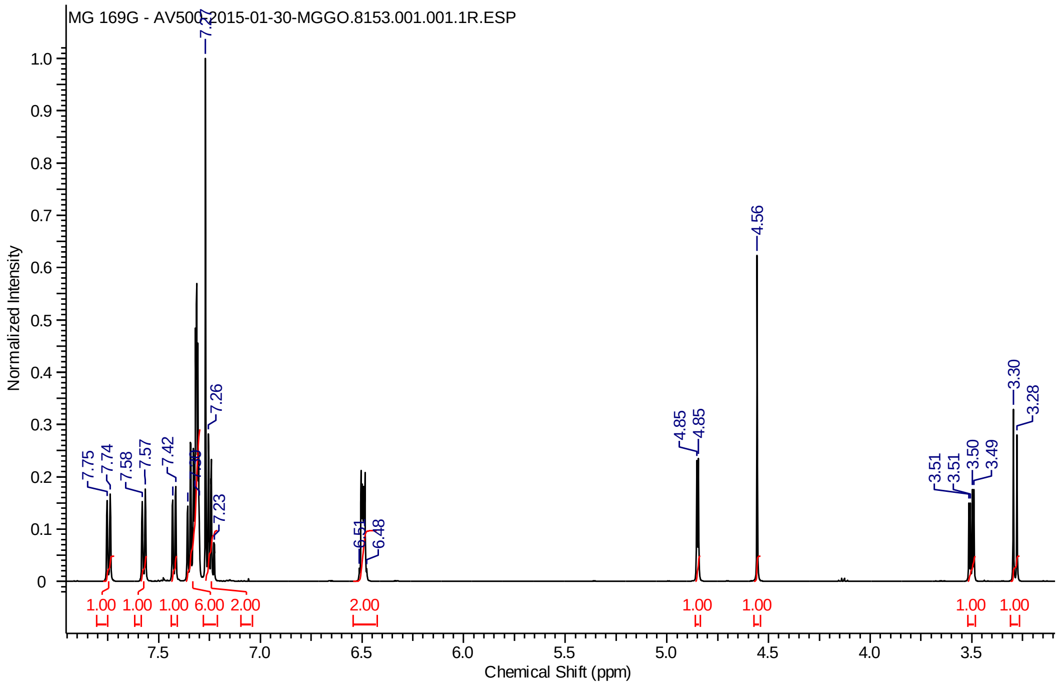

Compound *rac*-**26**:

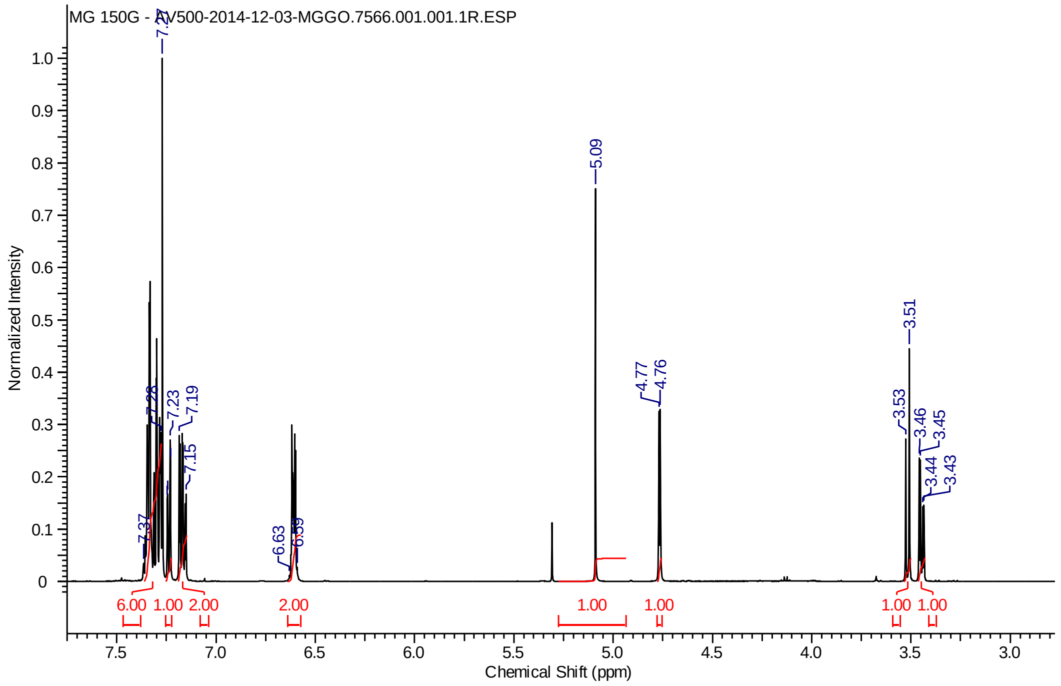

Compound *rac*-**27**:

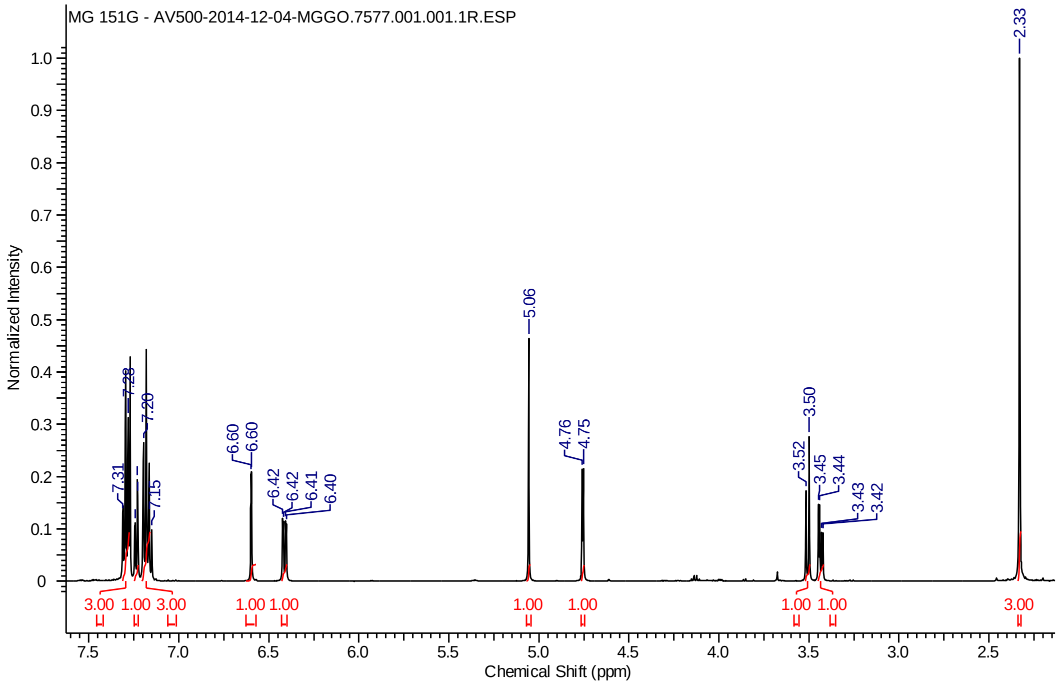

Compound *rac*-**28**:

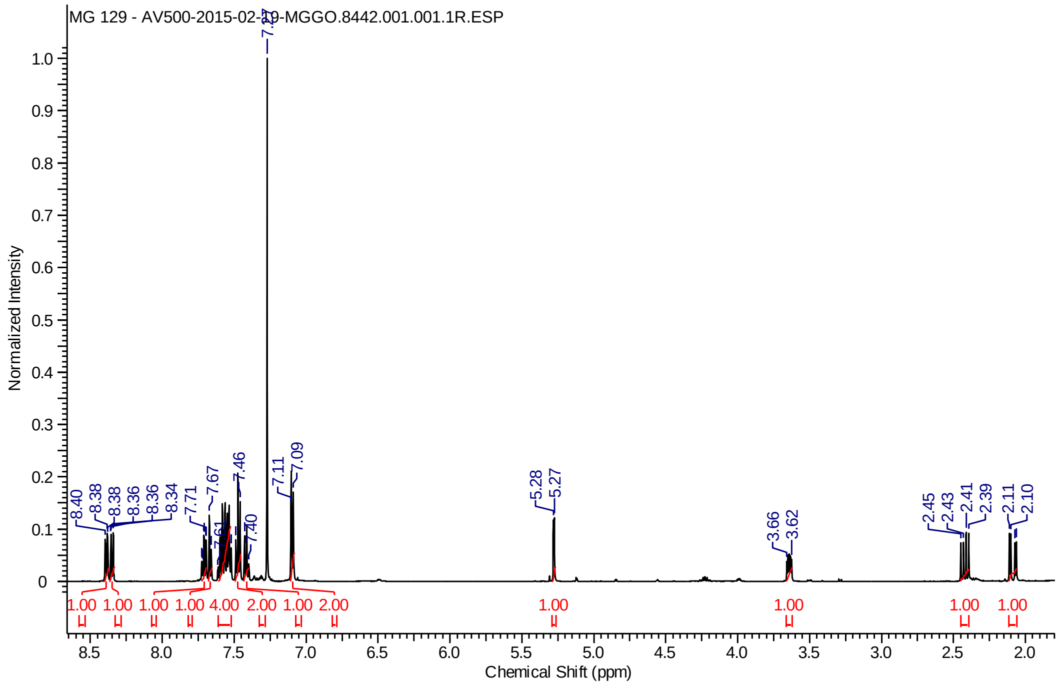

Compound *rac*-**29**:

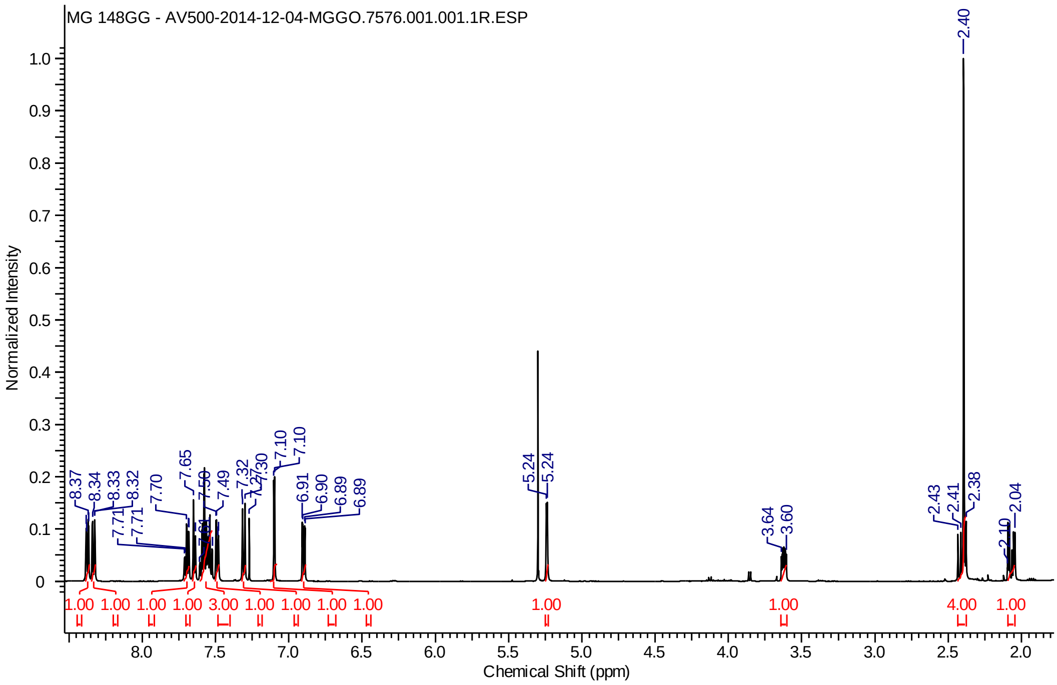

Compound *rac*-**30**:

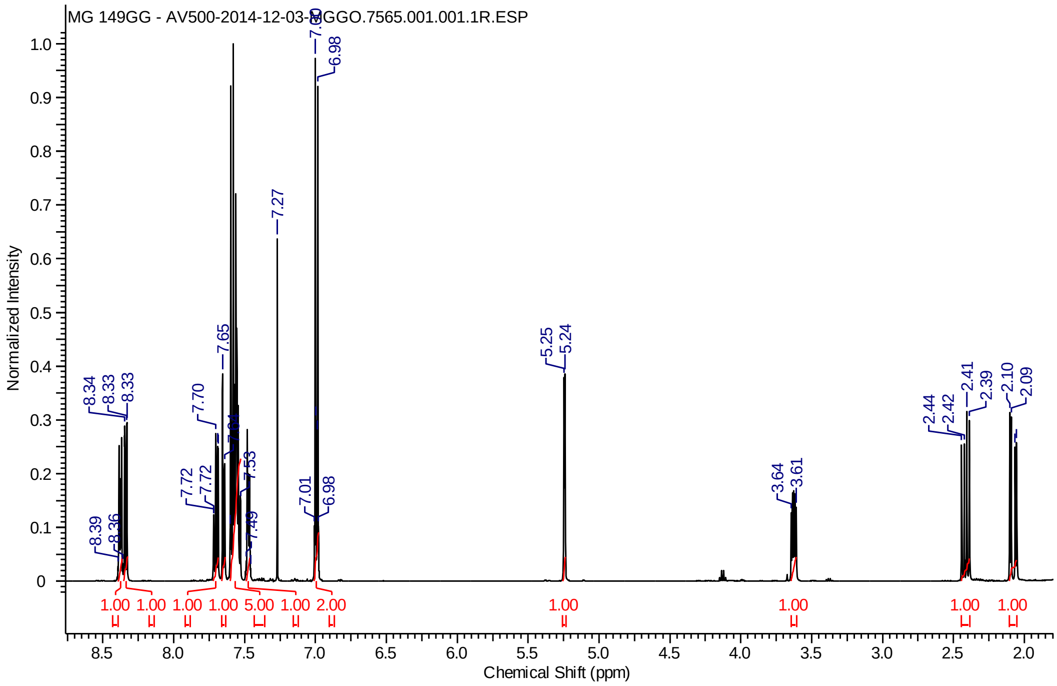

Compound *rac*-**31**:

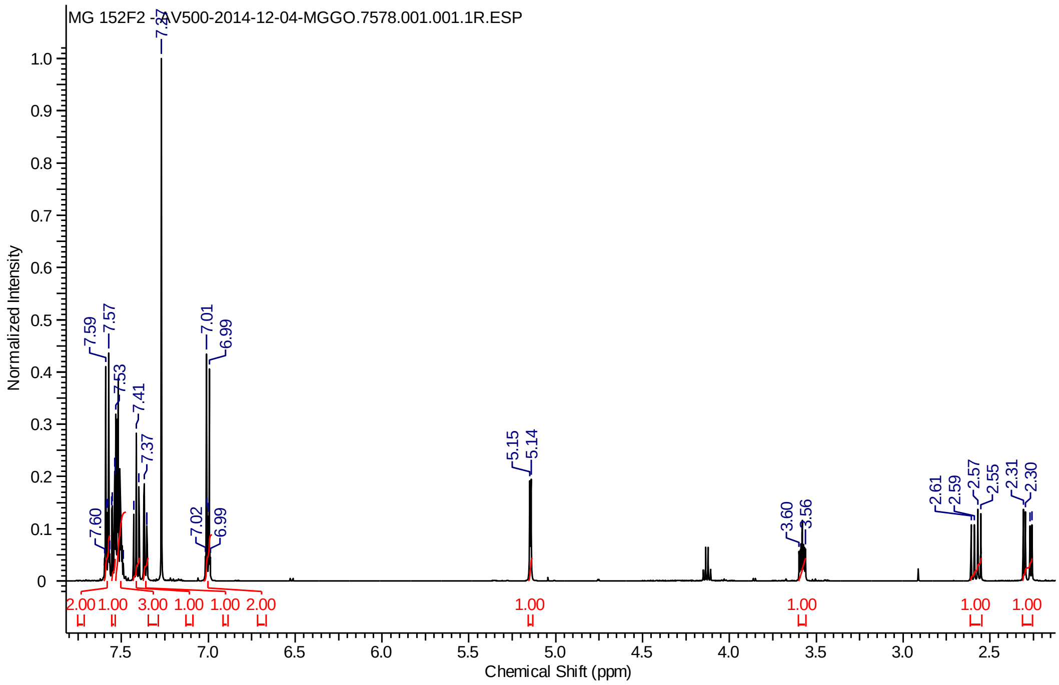

Supplement: File 1 — Synthetic procedures, characterization data, copies of chromatograms on chiral columns and of 1H and 13C NMR spectra. [file Beilstein_J_Org_Chem-12-1870-s001.pdf]
